# Methyltrimethoxysilane (MTM) as a Reagent for Direct Amidation of Carboxylic Acids

**DOI:** 10.1021/acs.orglett.1c04265

**Published:** 2022-01-27

**Authors:** D. Christopher Braddock, Joshua J. Davies, Paul D. Lickiss

**Affiliations:** Department of Chemistry, Molecular Sciences Research Hub, Imperial College London, White City Campus, 82 Wood Lane, London W12 0BZ, U.K.

## Abstract

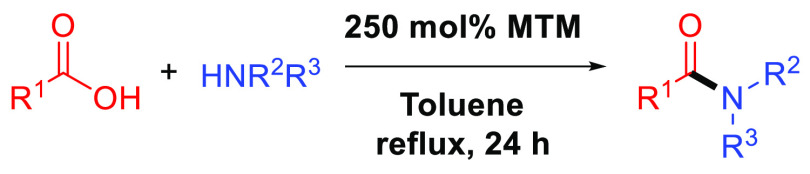

Methyltrimethoxysilane [MTM, CH_3_Si(OMe)_3_] has been demonstrated to be an effective, inexpensive, and safe reagent for the direct amidation of carboxylic acids with amines. Two simple workup procedures that provide the pure amide product without the need for further purification have been developed. The first employs an aqueous base-mediated annihilation of MTM. The second involves simple product crystallization from the reaction mixture providing a low process mass intensity direct amidation protocol.

The direct amidation of carboxylic acids with amines is a topic of much ongoing interest,^1^ due to the importance of the amide bond in medicinal chemistry^2^ and in the pharmaceutical industry.^3^ State-of-the-art protocols include thermal amidations,^4^ boron-based catalysts^5^ and reagents,^6^ oxophilic transition metal catalysts,^7^ silicon-based reagents,^8^ and others.^9^ However, the search for a sustainable direct amidation reagent that is nontoxic, inexpensive, and widely available affording amide products in high yields with all acid–amine combinations and proceeds with an overall low process mass intensity (PMI) that avoids chromatography continues.^10^ Toward that end, we have recently reported the use of tetramethylorthosilicate [TMOS, Si(OMe)_4_] (**1**) as a reagent for direct amidation.^11^ TMOS is inexpensive and widely available, successfully mediates direct amidation of aromatic and aliphatic carboxylic acids with primary amines, secondary amines, and anilines in an ideal 1:1 stoichiometry, and is annihilated to silica in a simple aqueous workup procedure that delivers the amide product in pure form without the need for chromatographic purification. However, because hydrolysis of TMOS to silica in the lung induces silicosis, TMOS is considered fatal if inhaled (GHS H330), thereby reducing its attractiveness. Accordingly, we envisioned employing an alternative silicon-based reagent that retains the inherent reactivity of TMOS but cannot undergo hydrolysis to silica and is still amenable to removal in a workup procedure. Herein, we present methyltrimethoxysilane [MTM, MeSi(OMe)_3_] (**2**) as a safer (and, in fact, cheaper) alternative to TMOS for the sustainable direct amidation of carboxylic acids with amines (Figure 1).

**Figure 1:**
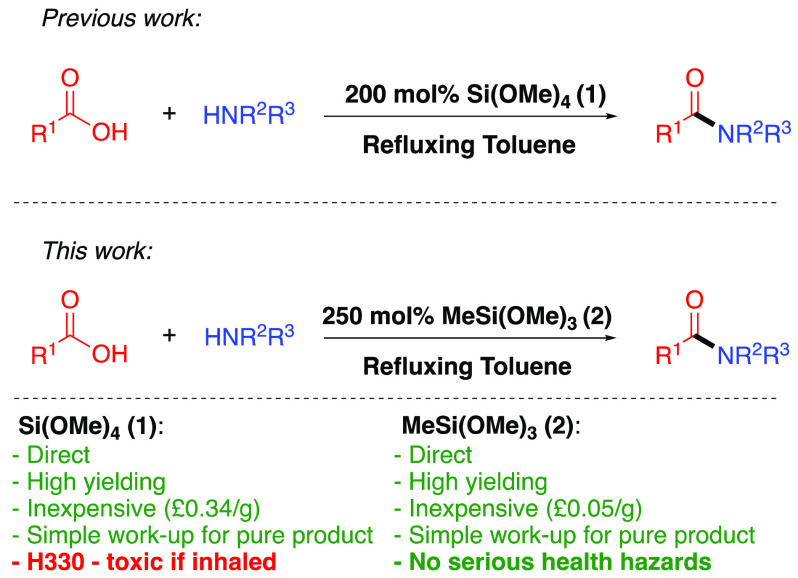
Previous work developed by Braddock et al. utilizing Si(OMe)_4_ (**1**) as a reagent for direct amidation. This work utilizes MeSi(OMe)_3_ (**2**).

Phenylacetic acid and benzoic acid were chosen as representative acids to be amidated using MTM (**2**) with a representative primary amine, a secondary cyclic amine, a secondary acyclic amine, and an aniline to enable a direct comparison with the use of TMOS (**1**).^11^ While we were hopeful that this single “methoxy-to-methyl” switch would not be too deleterious to reactivity, we anticipated that the workup procedure would require significant modification to deal with the complex mixture of linear and cyclic polysiloxanes known to result in hydrolysis of MTM (**2**).^12^ In the event, the use of 250 mol % MTM (for optimization of MTM loading, see the Supporting Information) in refluxing toluene provided pure amide products **3–10** directly after a suitably modified workup (for development of the workup procedure, see the Supporting Information). Specifically, evaporation of the reaction mixture postreaction removes the solvent as well as siloxane (MeO)_2_MeSi-O-SiMe(OMe)_2_ and methanol as the expected stoichiometric byproducts of the amidation process.^13^ Nonvolatile oligomeric polysiloxanes were found to be completely removed after subsequent stirring of the residue in a homogeneous THF/aqueous NaOH solution for 1 h, where any unwanted methyl ester side product also undergoes hydrolysis. Any unreacted carboxylic acid is also removed in this step, and any unreacted amine is removed in a subsequent aqueous acid wash. This workup procedure thereby provides the amide products in pure form without the need for any further purification regardless of the extent of amidation reaction conversion.

Inspection of the isolated yields for amides **3–10** shows that MTM is as effective as TMOS (**1**) as a reagent for amidation of the representative aliphatic carboxylic acid with all of the main amine classes (Figure 2). The use of benzoic acid as a representative, less reactive, aromatic carboxylic acid was a high yield with a primary amine but less successful with secondary amines, and in contrast to the case of TMOS,^11^ the attempted use of 4 Å molecular sieves for these amidations in the reaction mixture or suspended in the headspace proved to be detrimental. Pleasingly, the use of both aliphatic and aromatic carboxylic acids with aniline provided the amide products in good yields.^14^

**Figure 2:**
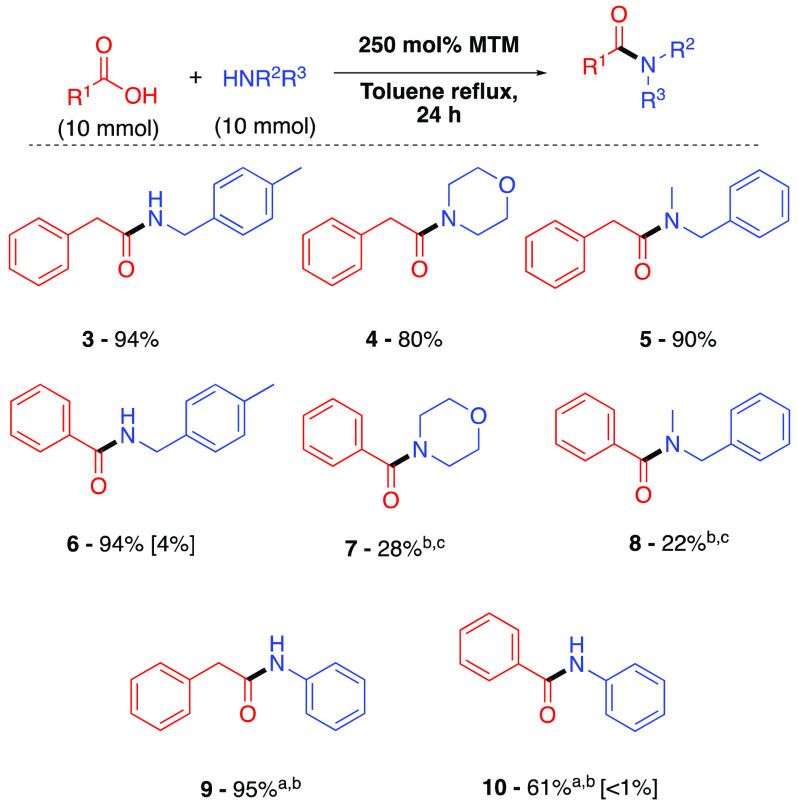
MeSi(OMe)_3_ (**2**)-mediated direct amidation of representative carboxylic acids and amines with 1 M acid and 1 M amine. ^a^With 2 equiv of acid. ^b^With 2 M amine. The isolated yield from a background reaction (i.e., without MTM) is given in brackets. ^c^With fractional distillation of MeOH.

Further exemplification of the MTM direct amidation method gave amides **11–26** (Figure 3A). These include examples of amide formation using branched carboxylic acids and amines, heteroaromatic and ferrocenyl-containing entities, halogenated substrates, and unsaturated carboxylic acids. Notably, both *N*-Cbz- and *N*-Boc-protected amino acids underwent successful amidation^15^ to give amides **22** and **23**, respectively, without racemization. It is important to emphasize that all of these amidations were conducted on a gram scale, where the devised workup procedure gave the pure amide product without the requirement for chromatography. However, attempts to (doubly) amidate malonic acid, or an α-hydroxy acid,^16^ to form a Weinreb amide^17^ or to use a low-boiling point amine^18^ under these conditions gave amides **27–30** (Figure 3B) in only low yield, albeit pure directly after workup, and these experiments show the current limits of the method.

**Figure 3:**
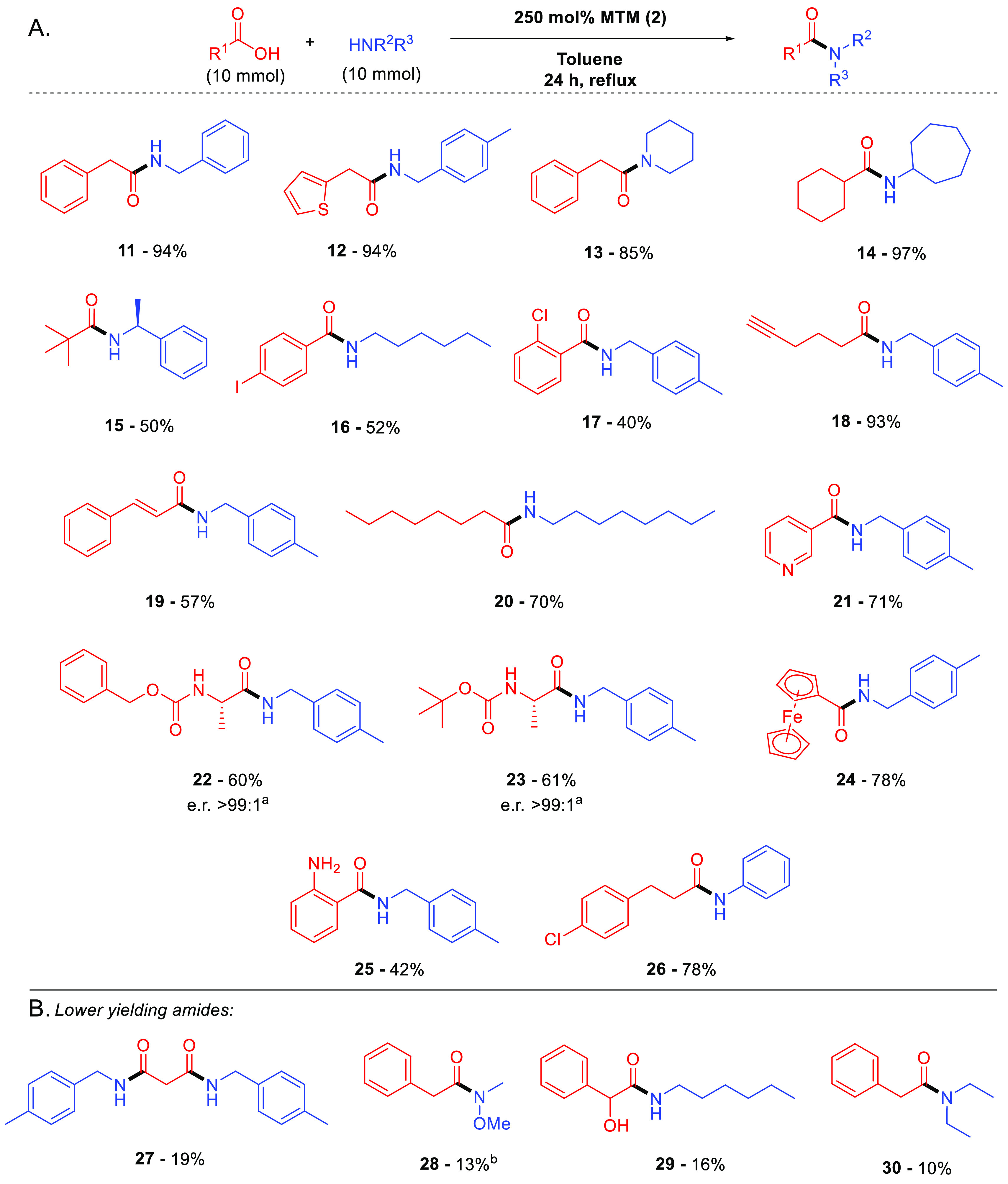
(A) Expanded scope of MeSi(OMe)_3_ (**2**)-mediated amidation of carboxylic acids and amines with 1 M acid and 1 M amine. (B) Amides formed in lower yields. ^a^The er was determined by HPLC analysis on a chiral stationary phase by reference to an authentic racemic sample. ^b^One equivalent of NEt_3_ was added to liberate amine from HCl salt.

As part of these investigations, we discovered that several secondary amide products crystallized from their reaction mixtures on cooling where residual MTM and its byproducts remained in solution. This allowed isolation of the pure amide product directly by filtration (and a hexane wash) without the need for any further workup, thereby resulting in low PMI values as exemplified for amides **31** and **32** (Figure 4).^19^ To the best of our knowledge, this is the first demonstration of insolubility of secondary amides in toluene being utilized for product isolation in an amidation protocol, and we anticipate that it would be widely applicable to other secondary amide products.

**Figure 4:**
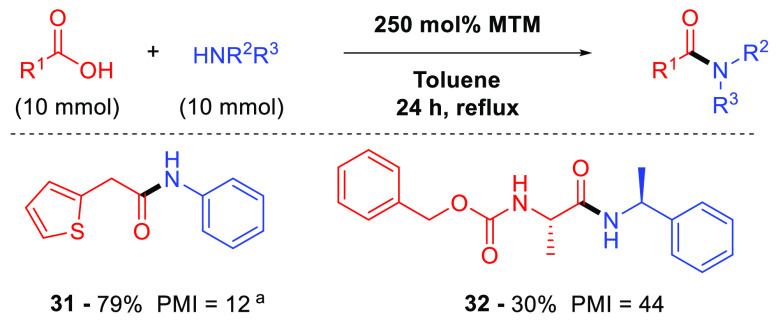
Low-PMI MeSi(OMe)_3_ (**2**)-mediated direct amidation of carboxylic acids and amines with 1 M acid and 1 M amine. ^a^On a 45 mmol scale with fractional distillation of MeOH.

Mechanistically, it has been proposed that amidations promoted by stoichiometric silicon reagents form silyl esters as activated intermediates.^8^ We therefore propose that these amidations take place by reversible reaction of the carboxylic acid with MTM to produce a silyl ester of type **A** with loss of methanol, followed by subsequent irreversible attack by amine to form the amide product (Figure 5). The liberated silanol **B** evidently must undergo favorable condensation with a second equivalent of MTM to form siloxane **C** and a second equivalent of methanol. The observation of small quantities of methyl esters (which may themselves undergo amidation) in crude reaction mixtures implicates some competitive direct attack of silyl ester **A** by methanol. In support of this mechanistic proposal, reaction of phenylacetic acid with MTM (**2**) showed the formation of an intermediate with a ^1^H NMR shift at 0.42 ppm, which is consistent with assignment to the Si-CH_3_ of a silyl ester. The silyl ester was found to be completely consumed upon addition of amine with concomitant amide formation.^20^

**Figure 5:**
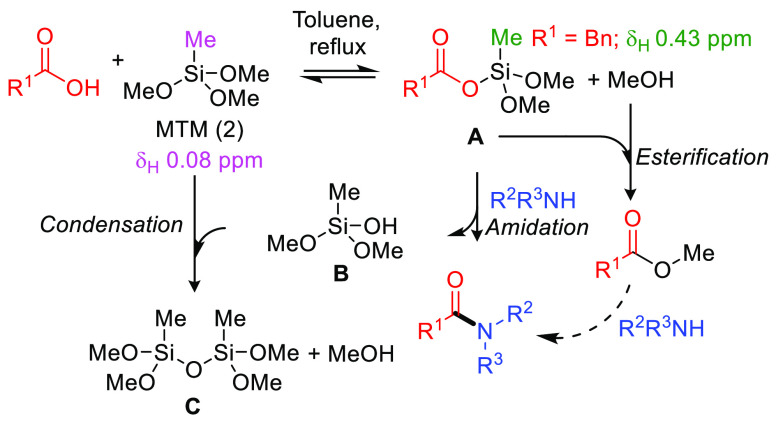
Postulated mechanism for MTM direct amidations.

In conclusion, we have reported the use of MTM (**2**) as an effective, inexpensive reagent for the direct amidation of carboxylic acids with amines, providing a safe alternative to the previously published protocol using TMOS (**1**). The amide products can be isolated in pure form either via a workup procedure that removes residual MTM and any linear and cyclic polysiloxane reaction byproducts or (in the case of secondary amides) by simple crystallization from the reaction mixture. We expect that the latter finding will be generally applicable to provide secondary amides by this method with low process mass intensities.^21^

## Supporting Information Available

General experimental section, optimization of MTM loading, optimization of the workup procedure, experimental details and characterization data for compounds, copies of ^1^H and ^13^C spectra for all compounds, and chiral HPLC analysis of amides **22** and **23** (PDF)
