# Supplementary material for: Methyltrimethoxysilane (MTM) as a Reagent for Direct Amidation of Carboxylic Acids
Source: Org Lett. 2022 Jan 27;24(5):1175–9. doi: 10.1021/acs.orglett.1c04265 (PMC9007566; doi:10.1021/acs.orglett.1c04265)
Supplement: Supplementary file 1 — ol1c04265_si_001.pdf [file ol1c04265_si_001.pdf]

Electronic Supporting Information

Methyltrimethoxysilane (MTM) as a Reagent for Direct Amidation of Carboxylic Acids

D. Christopher Braddock,*† Joshua, J. Davies, † Paul D. Lickiss*†

† Department of Chemistry, Molecular Sciences Research Hub, Imperial College London, White City Campus, 82 Wood Lane, London W12 0BZ, UK

Email Address: *c.braddock@imperial.ac.uk*, *p.lickiss@imperial.ac.uk*

Cover Page and Contents

pESI 1	Cover page and contents;
pESI 2	General experimental;
pESI 3	Optimization of MTM loading;
pESI 4	Optimization of work-up procedure;
pESI 5-18	Experimental details and characterizing data for compounds;
pESI 19-78	Copies of ^{1}H and ^{13}C spectra for all compounds;
pESI 79-80	HPLC analysis of enantiomeric purity of amides (*S*)-**22** and (*S*)-**23**;
pESI 81	References.

General Experimental

Reagents: Amine and carboxylic acids were purchased from commercial sources and used without further purification. Methyltrimethoxysilane (MTM) was purchased from commercial sources and used without further purification.

Solvents: Toluene (≥99.5% AnalaR NORMAPUR®), THF (≥99.5% AnalaR NORMAPUR®) and petroleum ether (≥95% GPR RECTAPUR®) were used as received.

Experimental Techniques: Oven-dried glassware was utilized for all reactions. All reactions were carried out under a positive pressure of nitrogen was unless stated otherwise. A dry-syn block and heating mantle were utilized for any reaction carried out at temperatures other than room temperature. 'Concentrated' refers to the concentrating of a solvent/product mixture by removing solvent and other unwanted volatile components *in vacuo* with the use of a rotary evaporator.

Characterisation: ^{1}H NMR and ^{13}C NMR spectra were recorded on a Bruker AV-400 or Bruker DRX-400. ^{1}H NMR were recorded at 400 MHz. ^{13}C NMR were recorded at 101 MHz. All chemical shifts (δ) expressed in ppm (parts per million) relative to the residual solvent peak (deuterated chloroform unless stated otherwise). Abbreviations for multiplicities are; s, singlet; d, doublet; t, triplet; q, quartet; m, multiplet. Fourier transform infra-red (IR) spectra were recorded neat using an ATR-IR spectrometer. Mass spectra are recorded by the Imperial College Department of Chemistry Mass Spectroscopy service. Melting points were recorded on a Stuart Melting Point Apparatus (SMP10).

Optimisation of MTM loading:

Table 1: Screen of reagent loading of MTM for amide bond formation.

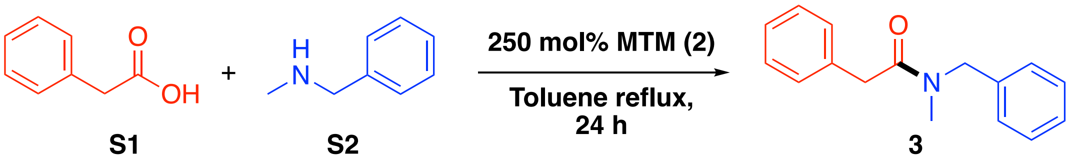

S1 (10 mmol) **S2** (10 mmol) **3**

Entry[a]	Mol % MTM	Yield of amide (%)[b]
1	200	80
2	250	90
3	300	91

Reaction conditions: [a] toluene, reflux, 24 h, N_2 (g); [**S1**] = 1 M, [**S2**] = 1 M. [b] Isolated yield after work-up.

Optimisation of work-up conditions:

Table 2: Development of work-up procedure for MTM mediated amidation

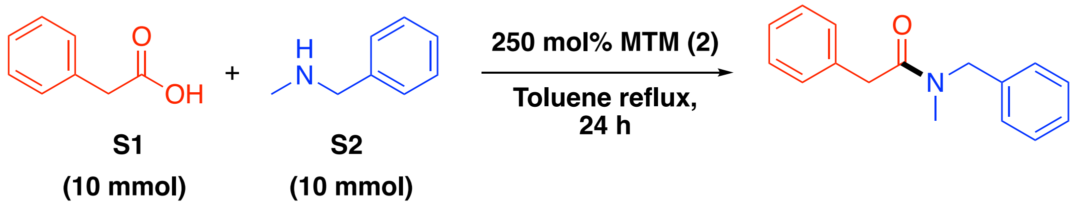

Entry	Volume of THF for dilution	Volume of base (0.3 M) wash	Base wash time (min)	Acid	Volume of acid wash	Extraction solvent	Yield (%)	PMI
1	50 mL	K_2CO_3 (150 mL)	1	HCl 1M	50 mL	THF	Impure [b,c]	-
2[a]	40 mL	NaOH (40 mL)	1	Sat. aq. NH_4Cl	40 mL	THF	67%	529
3	40 mL	NaOH (100 mL)	1	Sat. aq. NH_4Cl	100 mL	THF	Impure [b]	-
4	40 mL	NaOH (80 mL)	1	Sat. aq. NH_4Cl	100 mL	THF	Impure [b,c]	-
5	40 mL	NaOH (100 mL)	60	Sat. aq. NH_4Cl	100 mL	THF	78%	172
6	40 mL	NaOH (100 mL)	60	Sat. aq. NH_4Cl	50 mL	THF	75%	150
7	40 mL	NaOH (100 mL)	60	Sat. aq. NH_4Cl	10 mL	THF	75%	127
8	40 mL	NaOH (100 mL)	60	HCl 1M	10 mL	THF	78%	123
9	40 mL	NaOH (100 mL)	60	HCl 1M	10 mL	EtOAc	Impure [d]	-
10	40 mL	NaOH (100 mL)	60	HCl 1M	10 mL	Et_2O	79%	122
11	20 mL	NaOH (100 mL)	60	HCl 1M	10 mL	Et_2O	84%	105
12	10 mL	NaOH (100 mL)	60	HCl 1M	10 mL	Et_2O	90%	94

[a] 2 mmol of acid and amine is used, hence the higher PMI; [b] Ester ($PhCH_2COOMe$) side product present after work-up; [c] polymeric siloxane impurities observed by ^1H NMR (0.05 - 0.40 ppm). [d] Acetic acid present after work-up. Attempted dilution with just 5 mL of THF resulted in impure product.

Experimental Procedures and Characterizing Data for Compounds

General Procedure A

Acid (10 mmol, 1 equiv.), amine (10 mmol, 1 equiv.) and MTM **2** (25 mmol, 2.5 equiv.) were heated to reflux in toluene (1 M). After 24 h, the solution was allowed to cool and concentrated *in vacuo*. The residue was diluted with THF (10 mL) and aqueous NaOH solution (100 mL, 0.3 M) was added with stirring for 1 h. Et_2O (10 mL) was added, the aqueous phase was saturated with NaCl (s) and the layers were separated. The aqueous layer was extracted with Et_2O (35 mL), and the combined organics were washed with aqueous HCl (10 mL, 1 M). The aqueous layer was extracted with Et_2O (25 mL) and the combined organics were dried over $MgSO_4$, filtered, and concentrated *in vacuo*.

General Procedure B

Acid (10 mmol, 1 equiv.), amine (10 mmol, 1 equiv.) and MTM **2** (25 mmol, 2.5 equiv.) were heated to reflux in toluene (1 M). After 24 h, The solution was allowed to cool and the resulting amide precipitate was filtered under vacuum and washed with petroleum ether.

N-(4-Methylbenzyl)-2-phenylacetamide (3)[1]

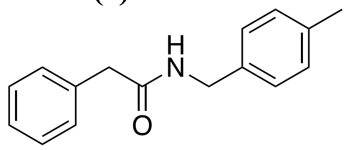

Following General Procedure A gave amide **3** (2.25 g, 9.4 mmol, 94%) as a white solid: m.p. 138.8 – 140.2 ˚C (lit.[1] m.p. 139.0 - 139.3°C); ATR-FTIR ν_{max}/cm^{-1} (neat) 3226, 1627; ^{1}H NMR (400 MHz, $CDCl_3$) δ 7.39-7.08 (m, 9H), 5.67 (br s, 1H), 4.40 (d, 2H, J = 5.7 Hz), 3.64 (s, 2H), 2.34 (s, 3H); ^{13}C NMR (101 MHz, $CDCl_3$) δ 170.8, 137.2, 135.1, 134.8, 129.5, 129.3, 129.1, 127.5, 127.4, 43.9, 43.4, 21.1; HRMS (APCI, orbitrap) *m/z*: $(M+H)^+$ calcd. for $C_{16}H_{18}ON$ 240.1383; Found 240.1385. Following General Procedure B gave amide **3** (2.30 g, 9.6 mmol, 96%) as a white solid.

1-Morpholino-2-phenylethan-1-one (4)[1]

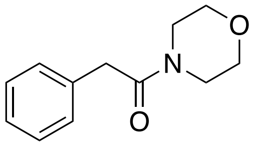

Following General Procedure A gave amide **4** (1.64 g, 8.0 mmol, 80%) as a white solid: m.p. 66.8 – 67.9 ˚C (lit.[1] m.p. 65 – 67 ˚C); ATR-FTIR ν_{max}/cm^{-1} (neat) 1638; ^{1}H NMR (400 MHz, $CDCl_3$) δ 7.37 - 7.32 (m, 2H), 7.29 - 7.25 (m, 3H), 3.75 (s, 2H), 3.66 (s, 4H), 3.50 - 3.45 (m, 4H); ^{13}C NMR (101 MHz, $CDCl_3$) δ 169.7, 134.8, 128.8, 128.5, 126.9, 66.8, 66.5, 46.5, 42.2, 40.9; HRMS (ES^+, TOF) *m/z*: $(M+H)^+$calcd. for $C_{12}H_{16}O_2N$ 206.1181; Found 206.1186.

N-Benzyl-N-methyl-2-phenylacetamide (5)[1]

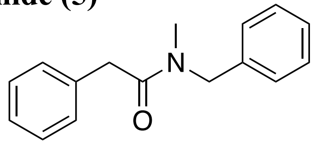

Following General Procedure A gave amide **5** (2.17 g, 9.0 mmol, 90%) as a white solid: m.p. 61.3 ˚C - 63.9 ˚C (lit.[1] m.p. 63 - 64 ˚C); ATR-FTIR ν_{max}/cm^{-1} (neat) 1624; ^{1}H NMR (400 MHz, $CDCl_3$, mixture of

two rotamers 1.4:1) δ (Major rotamer) 7.38-7.12 (m, 10H), 4.64 (s, 2H), 3.82 (s, 2H), 2.93 (s, 3H). (Minor rotamer) 7.38-7.12 (m, 10H), 4.56 (s, 2H), 3.79 (s, 2H), 2.98 (s, 3H); ^{13}C NMR (101 MHz, $CDCl_3$) δ 171.5, 171.2, 137.3, 136.5, 135.1, 135.0, 128.9, 128.9, 128.8, 128.7, 128.6, 128.1, 127.7, 127.4, 126.9, 126.8, 126.4, 53.7, 51.0, 41.2, 40.9, 35.2, 34.1; HRMS (APCI, orbitrap) *m/z*: $(M+H)^+$ calcd. for $C_{16}H_{18}ON$ 240.1383; Found 240.1381.

***N*-(4-Methylbenzyl)benzamide (6)**[1]

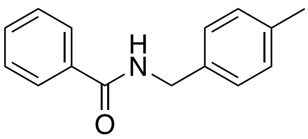

Following General Procedure A using 2 equivalents of carboxylic acid gave amide **6** (2.12 g, 9.4 mmol, 94%) as a white solid: m.p. 139.6 ˚C - 140.0 ˚C (lit.[1] m.p. 139.7 - 140.1 ˚C); ATR-FTIR ν_{max}/cm^{-1} (neat) 3287, 1634; ^{1}H NMR (400 MHz, $CDCl_3$) δ 7.82 - 7.80 (m, 2H), 7.54 – 7.50 (m, 1H), 7.45 – 7.43 (m, 2H), 7.29 - 7.27 (d, 2H, J = 7.8 Hz), 7.20 - 7.18 (d, 2H, J = 7.8 Hz), 6.43 (br s, 1H), 4.64 - 4.63 (d, 2H, J = 4.7 Hz), 2.38 (s, 3H); ^{13}C NMR (101 MHz, $CDCl_3$) δ 167.3, 137.4, 135.1, 134.5, 131.5, 129.5, 128.6, 128.0, 127.0, 44.0, 21.1; HRMS (ES$^+$, TOF) *m/z*: $(M+H)^+$ calcd for $C_{15}H_{15}ON$ 226.1232; Found 226.1240**.**

Morpholino(phenyl)methanone (7)[1]

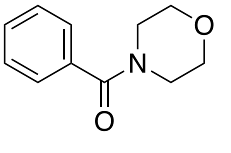

Following General Procedure A at 2 M concentration with fractional distillation of MeOH gave amide **7** (534 mg, 2.8 mmol, 28%) as a white solid: m.p. 71.4 - 72.8 ˚C (lit.[1] m.p. 73 - 75 ˚C); ATR-FTIR ν_{max}/cm^{-1} (neat) 1619; ^{1}H NMR (400 MHz, $CDCl_3$) δ 7.45 - 7.41 (m, 5H), 3.79 - 3.47 (m, 8H); ^{13}C NMR (101 MHz, $CDCl_3$) δ 170.5, 135.3, 129.9, 128.6, 127.1, 66.9, 48.3, 42.6; HRMS (ES$^+$, TOF) *m/z*: $(M+H)^+$ calcd for

$C_{11}H_{14}O_2N$ 192.1025; Found 192.1019.

***N*-Methyl-*N*-benzylbenzamide (8)**[1]

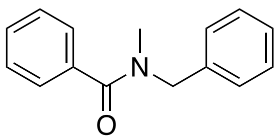

Following General Procedure A at 2 M concentration with fractional distillation of MeOH gave amide **8** (495 mg, 2.2 mmol, 22%) as a yellow oil: ATR-FTIR ν_{max}/cm^{-1} (neat) 1626; ^1H NMR (400 MHz, $CDCl_3$, mixture of two rotamers 1:1) δ 7.50 - 7.20 (m, 10H), 4.79 & 4.54 (2 singlets, rotamers, 2H), 3.06 & 2.89 (2 singlets, rotamers, 3H); ^{13}C NMR (101 MHz, $CDCl_3$) δ 172.4, 171.6, 137.1, 136.6, 136.2, 129.7, 128.9, 128.8, 128.5, 128.2, 127.6, 127.0, 126.8, 55.2, 50.8, 37.1, 33.2; HRMS (ES^+, TOF) *m/z*: $(M+H)^+$ calcd. for $C_{15}H_{16}ON$ 226.1232; Found 226.1226.

***N*,2-Diphenylacetamide (9)**[2]

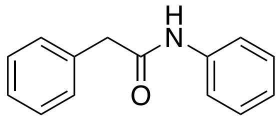

Following General Procedure A using 2 equivalents of acid, and at 2 M concentration followed by trituration with petroleum ether (3 x 10 mL) gave amide **9** (2.00 g, 9.5 mmol, 95%) as a white solid: m.p. 114.1 - 116.8 ˚C (lit.[2] m.p. 113 - 115 ˚C); ATR-FTIR ν_{max}/cm^{-1} (neat) 3248, 1652; ^1H NMR (400 MHz, $CDCl_3$) δ 7.45 - 7.41 (m, 3H), 7.38 - 7.36 (m, 3H), 7.33 - 7.29 (m, 2H), 7.21 (br s, 1H), 7.13 - 7.10 (m, 1H), 3.77 (s, 2H); ^{13}C NMR (101 MHz, $CDCl_3$) δ 169.1, 137.6, 134.4, 129.6, 129.3, 129.0, 127.7, 124.5, 119.9, 44.9; HRMS (ES^+, TOF) *m/z*: $(M+H)^+$ calcd. for $C_{14}H_{13}ON$ 212.1075; Found 212.1075.

***N*-Phenylbenzamide (10)**[3]

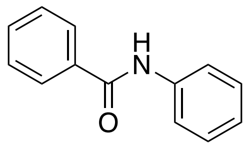

Following General Procedure A using 2 equivalents of acid, and at 2 M concentration followed by trituration with petroleum ether (3 x 10 mL) gave amide **10** (1.20 g, 6.1 mmol, 61%) as a white solid: m.p. 163.2 – 166.3 ˚C (lit.[3] m.p. 165-167 ˚C); ATR-FTIR ν_{max}/cm^{-1} (neat) 3337, 1651; ^{1}H NMR (400 MHz, $CDCl_3$) δ 7.90 - 7.88 (m, 3H), 7.68 - 7.66 (m, 2H), 7.59 - 7.55 (m, 1H), 7.52 - 7.48 (m, 2H), 7.41 - 7.37 (m, 2H), 7.20 - 7.16 (m, 1H); ^{13}C NMR (101 MHz, $CDCl_3$) δ 165.8, 137.9, 135.0, 131.9, 129.1, 128.8, 127.1, 124.6, 120.2; HRMS (ES$^+$, TOF) *m/z*: (M+H)$^+$ calcd. for $C_{13}H_{12}ON$ 198.0919; Found 198.0923.

***N*-Benzyl-2-phenylacetamide (11)**[4]

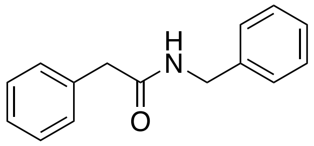

Following General Procedure A gave amide **11** (2.12 g, 9.4 mmol, 94%) as a white solid: m.p. 122.0 – 123.3 ˚C (lit.[4] m.p. 118-120 ˚C); ATR-FTIR ν_{max}/cm^{-1} (neat) 3275, 1633; ^{1}H NMR (400 MHz, $CDCl_3$) δ 7.40 - 7.25 (m, 8H), 7.21 - 7.19 (m, 2H), 5.72 (br s, 1H), 4.44 (d, 2H, J = 6.1 Hz), 3.66 (s, 3H); ^{13}C NMR (101 MHz, $CDCl_3$) δ 170.9, 138.1, 134.8, 129.5, 129.1, 128.7, 127.5, 127.6, 43.9, 43.6; HRMS (ES$^+$, TOF) *m/z*: (M+H)$^+$ calcd. for $C_{15}H_{15}ON$ 226.1232; Found 226.1234**.** Following General Procedure B gave amide **11** (2.16 g, 9.6 mmol, 96%) as a white solid.

N-(4-Methylbenzyl)-2-(thiophen-2-yl)acetamide (12)

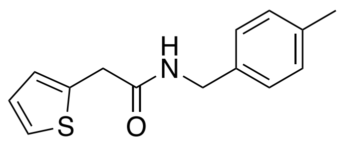

Following General Procedure B gave amide **12** (2.30 g, 9.4 mmol, 94%) as a white solid: m.p. 122.2 – 123.2; ˚C ATR-FTIR ν_{max}/cm^{-1} (neat) 3268, 1631; ^{1}H NMR (400 MHz, $CDCl_3$) δ 7.29 - 7.25 (m, 1H), 7.16 - 7.11 (m, 4H), 7.01 - 6.96 (m, 2H), 5.89 (br s, 1H), 4.42 (d, 2H, *J* = 6.0 Hz), 3.85 (s, 2H), 2.35 (s, 3H); ^{13}C NMR (101 MHz, $CDCl_3$) δ 169.7, 137.2, 136.1, 134.9, 129.4, 127.6, 127.5, 127.4, 125.7, 43.5, 37.6, 21.1; HRMS (ES$^+$, TOF) *m/z*: $(M+H)^+$ calcd. for $C_{14}H_{16}ONS$ 246.0953; Found 246.0956.

2-Phenyl-1-(piperidin-1-yl)ethan-1-one (13)[5]

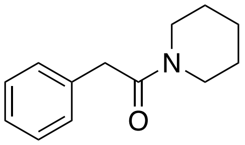

Following General Procedure A gave amide **13** (1.73 g, 8.5 mmol, 85%) as a yellow oil: ATR-FTIR ν_{max}/cm^{-1} (neat) 1625; ^{1}H NMR (400 MHz, $CDCl_3$) δ 7.36 - 7.32 (m, 2H), 7.29 - 7.24 (m, 3 H), 3.76 (s, 2H), 3.61 - 3.57 (m, 2H), 3.41 - 3.38 (m, 2H), 1.63 - 1.54 (m, 4H), 1.37 (m, 2H); ^{13}C NMR (101 MHz, $CDCl_3$) δ 169.3, 135.4, 128.7, 128.6, 126.7, 47.3, 42.9, 41.2, 26.2, 25.5, 24.4; HRMS (ES$^+$, TOF) *m/z*: $(M+H)^+$ calcd. for $C_{13}H_{18}ON$ 204.1388; Found 204.1388.

N-Cycloheptylcyclohexanecarboxamide (14)[6]

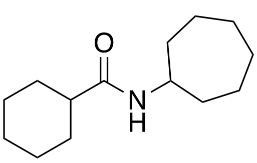

Following General Procedure A gave amide **14** (2.16 g, 9.7 mmol, 97%) as a white solid: m.p. 174.3 –

176.3 ˚C (lit.[6] m.p. 177.1-177.1 ˚C); ATR-FTIR ν_{max}/cm^{-1} (neat) 3250, 1627; ^{1}H NMR (400 MHz, $CDCl_3$) δ 5.41 (br s, 1H), 3.97 - 3.93 (m, 1H), 2.05 - 1.99 (tt, 1H, *J* = 11.6, 3.3 Hz) 1.92 - 1.78 (m, 6H), 1.69 - 1.37 (m, 14H), 1.32 - 1.20 (m, 3H); ^{13}C NMR (101 MHz, $CDCl_3$) δ 174.9, 50.0, 45.6, 35.2, 29.7, 28.0, 25.8, 24.1; HRMS (ES$^+$, TOF) *m/z*: $(M+H)^+$ calcd. for $C_{14}H_{26}ON$ 224.2014; Found 224.2020.

(*S*)-*N*-(1-Phenylethyl)pivalamide (15)[7]

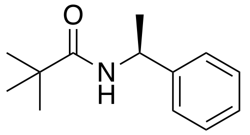

Following General Procedure A gave amide **15** (1.03 g, 5.0 mmol, 50%) as a white solid: m.p. 118.4-120.0 ˚C (lit.[6] m.p. 117-118˚C); $[\alpha]_D^{25.9}$= -72 (*c* 0.26, $CHCl_3$); ATR-FTIR ν_{max}/cm^{-1} (neat) 3329, 1632; ^{1}H NMR (400 MHz, $CDCl_3$) δ 7.39 - 7.27 (m, 5H), 5.83 (br s, 1H), 5.17 – 5.10 (m, 1H), 1.51 (d, 3H, *J* = 6.8 Hz), 1.22 (s, 9H); ^{13}C NMR (101 MHz, $CDCl_3$) δ 177.5, 143.5, 128.7, 127.3, 126.1, 48.5, 38.6, 27.6, 21.7; HRMS (ES$^+$, TOF) *m/z*: $(M+H)^+$ calcd. for $C_{13}H_{20}ON$ 206.1545; Found 206.1549.

***N*-Hexyl-4-iodobenzamide (16)**

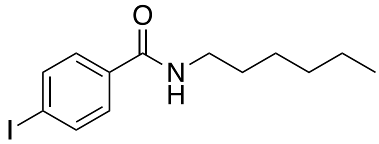

Following General Procedure A gave amide **16** (1.72 g, 5.2 mmol, 52%) as a yellow solid; m.p. 106.8-108.2 ˚C; ATR-FTIR ν_{max}/cm^{-1} (neat) 3324, 1624; ^{1}H NMR (400 MHz, $CDCl_3$) δ 7.78 (d, 2H, *J* = 8.5 Hz), 7.50 (d, 2H, *J* = 8.5 Hz), 6.27 (br s, 1H), 3.44 (q, 2H, *J* = 7.2 Hz), 1.65 - 1.57 (m, 2H), 1.41 - 1.30 (m, 6H), 0.92 - 0.89 (m, 3H); ^{13}C NMR (101 MHz, $CDCl_3$) δ 166.8, 137.7, 134.2, 128.5, 98.2, 40.2, 31.5, 29.6, 26.7, 22.6, 14.1; HRMS (ES$^+$, TOF) *m/z*: $(M+H)^+$ calcd. for $C_{13}H_{19}ONI$ 332.0511; Found 332.0512.

2-Chloro-*N*-(4-methylbenzyl)benzamide (17)[8]

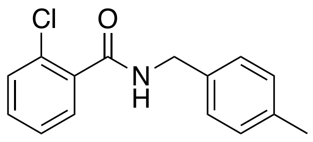

Following General Procedure A gave amide **17** (1.04 g, 4.0 mmol, 40%) as a white solid: m.p. 119.8–122.0 ˚C (lit.[8] 124-125 ˚C); ATR-FTIR ν_{max}/cm^{-1} (neat) 3248, 1634; ^{1}H NMR (400 MHz, $CDCl_3$) δ 7.70 (d, 1H, *J* = 7.42 Hz) , 7.43 - 7.18 (m, 7 H), 6.49 (br s, 1H), 4.65 (d, 1H, *J* = 5.3 Hz), 2.38 (s, 3H); ^{13}C NMR (101 MHz, $CDCl_3$) δ 166.4, 137.4, 135.0, 134.6, 131.4, 130.7, 130.2, 129.5, 127.9, 127.1, 44.1, 21.2; HRMS (ES$^+$, TOF) *m/z*: $(M+H)^+$ calcd. for $C_{15}H_{15}ONCl$ 260.0842; Found 260.0839**.**

***N*-(4-Methylbenzyl)hex-5-ynamide (18)**

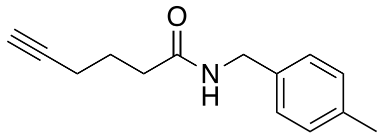

Following General Procedure A gave amide **18** (2.00 g, 9.3 mmol, 93%) as a white solid: m.p. 77.8 – 80.0 ˚C; ATR-FTIR ν_{max}/cm^{-1} (neat) 3287, 1629; ^{1}H NMR (400 MHz, $CDCl_3$) δ 7.21 - 7.15 (m, 4H), 5.91 (br s, 1H), 4.42 (d, 2H, *J* = 5.4 Hz*),* 2.39 - 2.35 (m, 5H), 2.29 (td, 2H, *J* = 6.9, 2.6 Hz), 1.98 (t, 1H, *J* = 2.6 Hz), 1.94 - 1.87 (m, 2H); ^{13}C NMR (101 MHz, $CDCl_3$) δ 172.1, 137.3, 135.2, 129.4, 127.9, 83.5, 69.2, 43.4, 35.0, 24.2, 21.1, 17.9; HRMS (ES$^+$, TOF) *m/z*: $(M+H)^+$ calcd. for $C_{14}H_{18}ON$ 216.1388; Found 216.1393.

***N*-(4-Methylbenzyl)cinnamamide (19)**[9]

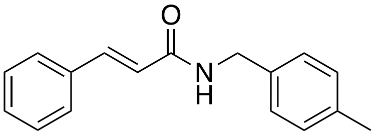

Following General Procedure A gave amide **19** (1.43 g, 5.7 mmol, 57%) as a white solid: 147 – 149 ˚C (lit.[9] m.p. 117-119 ˚C); ATR-FTIR v_{max}/cm^{-1} (neat) 3253, 1654; ^{1}H NMR (400 MHz, $CDCl_3$) δ 7.70 (d, 2H, *J* = 15.9 Hz), 7.53 - 7.51 (m, 2H), 7.39 - 7.38 (m, 3H), 7.25 (d, 2H, *J* = 7.9 Hz), 7.18 (d, 2H, *J* = 7.9 Hz), 6.43 (d, 2H, *J* = 15.9 Hz), 5.95 (br s, 1H), 4.56 (d, 2H, *J* = 5.7 Hz), 2.37 (s, 3H); ^{13}C NMR (101 MHz, $CDCl_3$) δ 165.7, 141.4, 137.4, 135.1, 134.8, 129.7, 129.5, 128.8, 128.0, 127.8, 120.5, 43.7, 21.1; HRMS (ES^+, TOF) *m/z*: $(M+H)^+$ calcd. for $C_{17}H_{18}ON$ 252.1388; Found 252.1396.

***N*-Octyloctanamide (20)**[10]

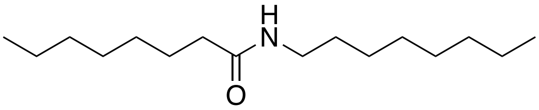

Following General Procedure A gave amide **20** (1.79 g, 7.0 mmol, 70%) as a white solid; m.p. 47.0 – 48.3 ˚C; ATR-FTIR v_{max}/cm^{-1} (neat) 3309, 1632; ^{1}H NMR (400 MHz, $CDCl_3$) δ 5.98 (br s, 1H), 3.22 (q, 2H, *J* = 6.9 Hz), 2.17 (t, 2H, *J* = 7.3 Hz), 1.66 - 1.58 (m, 2H), 1.51 - 1.46 (m, 2H), 1.29 - 1.21 (m, 18H), 0.89 - 0.86 (m, 6H); ^{13}C NMR (101 MHz, $CDCl_3$) δ 173.3, 39.5, 36.8, 31.9, 31.7, 29.6, 29.3, 29.2, 29.0, 27.0, 25.9, 22.6(2), 22.5(9), 14.0; HRMS (ES^+, TOF) *m/z*: $(M+H)^+$ calcd. for $C_{16}H_{34}ON$ 256.2646; Found 256.2640.

***N*-(4-Methylbenzyl)nicotinamide (21)**[1]

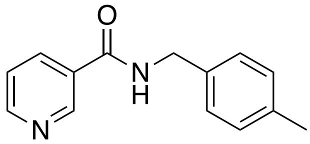

Following General Procedure A gave amide **21** (1.60 g, 7.1 mmol, 71%) as a white solid; m.p. 74.6 – 77.8

˚C (lit.[1] 79 – 81); ATR-FTIR ν_{max}/cm^{-1} (neat) 3298, 1654; ^{1}H NMR (400 MHz, $CDCl_3$) δ 8.54 (d, 1H, *J* = 5.2 Hz), 8.39 (br s, 1H), 8.26 (d, 1H, *J* = 7.7 Hz) , 7.88 (t, 1H, *J* = 7.7 Hz), 7.44 (t, 1H, *J* = 7.7 Hz), 7.29 (d, 2H, *J* = 7.7 Hz), 7.18 (d, 2H, *J* = 7.7 Hz), 4.66 (d, 2H, *J* = 6.1 Hz), 2.36 (s, 3H); ^{13}C NMR (101 MHz, $CDCl_3$) δ 164.1, 149.9, 148.0, 137.4, 137.2, 135.2, 129.4, 127.9, 126.2, 122.4, 43.3, 21.1; HRMS (ES$^+$, TOF) *m/z*: $(M+H)^+$ calcd. for $C_{14}H_{15}ON_2$ 227.1184; Found 227.1183.

Benzyl (*S*)-(1-(benzylamino)-1-oxopropan-2-yl)carbamate (22)

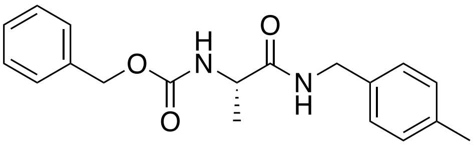

Following General Procedure A gave amide **22** (1.99 g, 6.0 mmol, 60%) as a white solid: m.p. 148 – 150 ˚C; $[\alpha]_D^{25.9}$= -5.1 (*c* 0.26, $CHCl_3$); ATR-FTIR ν_{max}/cm^{-1} (neat) 3279, 1689, 1637; ^{1}H NMR (400 MHz, $CDCl_3$) δ 7.39 - 7.31 (m, 5H), 7.16 - 7.11 (m, 4H), 6.63 (br s, 1H), 5.53 (br s, 1H), 5.06 - 4.99 (m, 2H), 4.40 - 4.29 (m, 3H), 2.33 (s, 3H), 1.41 (d, 3H, *J* = 7.0 Hz); ^{13}C NMR (101 MHz, $CDCl_3$) δ 172.2, 156.0, 137.3, 136.1, 134.8, 129.4, 128.6, 128.2, 128.1, 127.7, 67.0, 50.6, 43.3, 21.1, 18.9; HRMS (ES$^+$, TOF) *m/z*: $(M+H)^+$ calcd. for $C_{19}H_{23}O_3N_2$ 327.1709; Found 327.1695. HPLC (CHIRALPAK AD); 10% IPA in *n*-hexane; (1.0 mL/min) t_R = 13.7 min (major), 17.7 min (minor) – e.r. >99:1.

***tert*-Butyl (*S*)-(1-(benzylamino)-1-oxopropan-2-yl)carbamate (23)**

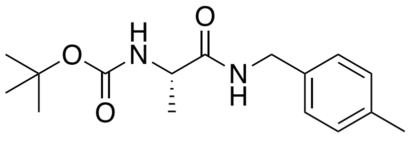

Following General Procedure A gave amide **23** (1.70 g, 6.1 mmol, 61%) as a white solid: m.p. 129.0 – 131.4 ˚C; $[\alpha]_D^{26.4}$= -21.3 (c 0.26, $CHCl_3$); ATR-FTIR ν_{max}/cm^{-1} (neat) 3377, 1688, 1652; ^{1}H NMR (400 MHz, $CDCl_3$) δ 7.15 - 7.14 (m, 4H), 6.69 (br s, 1H), 5.17 (br s, 1H), 4.40 (br s, 2H), 4.22 (br s, 1H), 2.34

(s, 3H), 1.43 (s, 9H), 1.38 (d, 3H, J = 7.0 Hz); ^{13}C NMR (101 MHz, $CDCl_3$) δ 172.6, 155.6, 137.1, 135.1, 129.3, 127.6, 80.1, 50.1, 43.2, 28.3, 21.1, 18.4; HRMS (ES$^+$, TOF) *m/z*: $(M+H)^+$ calcd. for $C_{16}H_{25}O_3N_2$ 293.1865; Found 293.1859. HPLC (CHIRALPAK AD); 10% IPA in *n*-hexane; (0.5 mL/min) t_R = 12.6 min (major), 15.0 min (minor) – e.r. >99:1.

N-(4-Methylbenzyl)ferrocene carboxamide (24)

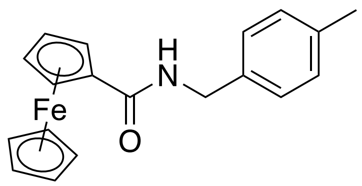

Following General Procedure A gave amide **24** (2.60 g, 7.8 mmol. 78%) an orange solid: m.p. 149.9 – 151.5 ˚C; ATR-FTIR ν_{max}/cm^{-1} (neat) 3280, 1627; ^{1}H NMR (400 MHz, $CDCl_3$) δ 7.29 - 7.20 (m, 4H), 5.95 (br s 1H), 4.71 (s, 2H), 4.57 (s, 2H), 4.38 (s, 2H), 4.22 (s, 5H), 2.38 (s, 3H); ^{13}C NMR (101 MHz, $CDCl_3$) δ 170.1, 137.2, 135.7, 129.4, 127.8, 76.0, 70.4, 69.6, 68.1, 43.3, 21.1.; HRMS (ES$^+$, TOF) *m/z*: $(M+H)^+$ calcd. for $C_{19}H_{20}ONFe$ 334.0894; Found 334.0886.

2-Amino-N-(4-methylbenzyl)benzamide (25)[11]

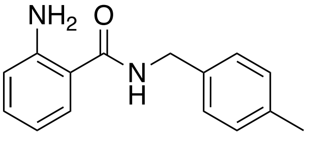

Following General procedure A gave amide **25** (1.01 g, 4.2 mmol, 42%) as a white solid: m.p. 152.8 – 155.0 ˚C; ATR-FTIR ν_{max}/cm^{-1} (neat) 3355, 3302, 1628; ^{1}H NMR (400 MHz, $CDCl_3$) δ 7.35 - 7.18 (m, 6H), 6.75 - 6.65 (m, 2H), 6.33 (br s, 1H), 4.59 (d, 2H, J = 5.5 Hz), 2.38 (s, 3H); ^{13}C NMR (101 MHz, $CDCl_3$) δ 169.0, 148.1, 137.3, 135.1, 132.4, 129.5, 127.9, 127.1, 117.7, 117.1, 116.3, 43.6, 21.1; HRMS (ES$^+$, TOF) *m/z*: $(M+H)^+$ calcd. for $C_{15}H_{17}ON_2$ 241.1341; Found 241.1334.

3-(4-Chlorophenyl)-*N*-phenylpropanamide (26)[12]

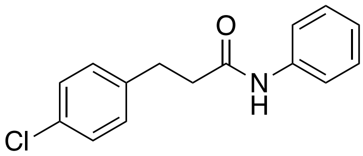

Following General Procedure A gave amide **26** (2.02 g, 7.8 mmol, 78%) as a white solid: m.p. 162-164 ˚C; ATR-FTIR ν_{max}/cm^{-1} (neat) 3271, 1651; ^{1}H NMR (400 MHz, $CDCl_3$) δ 7.47 (d, 2H, J = 7.8 Hz), 7.35 - 7.26 (m, 4H), 7.22 - 7.13 (m, 3H), 3.04 (t, 2H, J = 7.7 Hz) , 2.65 (t, 2H, J = 7.7 Hz); ^{13}C NMR (101 MHz, $CDCl_3$) δ 170.0, 139.1, 137.6, 132.1, 129.8, 129.0, 128.7, 124.5, 120.0, 39.2, 30.8; HRMS (ES$^+$, TOF) *m/z*: (M+H)$^+$ calcd. for $C_{15}H_{15}ONCl$ 260.0842; Found 260.0838. General procedure B gave amide **26** (2.02 g, 7.8 mmol, 78%) as a white solid.

***N*^{1},*N*^{3}-Bis(4-methylbenzyl)malonamide (27)**

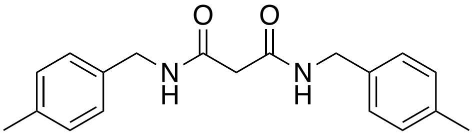

Following General Procedure A with 2 equiv. of amine gave amide **27** (0.59 g, 1.9 mmol, 19%) as a white solid: m.p. 224.4 – 227.0 ˚C; ATR-FTIR ν_{max}/cm^{-1} (neat) 3292, 1621; ^{1}H NMR (400 MHz, $CDCl_3$) δ 7.17 - 7.16 (m, 8H), 4.42 (d, 4H, J = 5.7 Hz), 3.26 (s, 2H), 2.36 (s, 6H); ^{13}C NMR (101 MHz, $CDCl_3$) δ 166.9, 137.3, 134.6, 129.4, 127.8, 43.5, 43.1, 21.1; HRMS (ES$^+$, TOF) *m/z*: (M+H)$^+$ calcd. for $C_{19}H_{23}O_2N_2$ 311.1760; Found 311.1747.

N-Methoxy-N-methyl-2-phenylacetamide (28)[13]

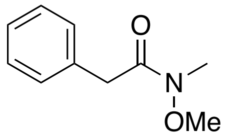

Following General Procedure A with 1 equivalent of triethylamine gave amide **28** (0.23 g, 1.3 mmol, 13%) as a yellow oil: ATR-FTIR ν_{max}/cm^{-1} (neat) 1655; ^{1}H NMR (400 MHz, $CDCl_3$) δ 7.35 - 7.29 (m, 5H), 3.81 (s, 2H), 3.63 (s, 3H), 3.22 (s, 3H); ^{13}C NMR (101 MHz, $CDCl_3$) δ 172.5, 134.9, 129.3, 128.5, 126.8, 61.3, 39.4, 32.3; HRMS (ES$^+$, TOF) *m/z*: $(M+H)^+$ calcd. for $C_{10}H_{14}O_2N$ 180.1025; Found 180.1017.

N-Hexyl-2-hydroxy-2-phenylacetamide (29)[14]

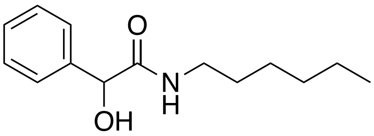

Following General Procedure A gave amide **29** (0.38 g, 1.6 mmol, 16%) as a white solid: m.p. 63.7 – 65.1 ˚C; ATR-FTIR ν_{max}/cm^{-1} (neat) 3295, 3199, 1615; ^{1}H NMR (400 MHz, $CDCl_3$) δ 7.42 - 7.36 (m, 5H), 6.10 (br s, 1H), 5.04 (s, 1H), 3.30 - 3.25 (m, 2H), 1.50 - 1.46 (m, 2H), 1.30 - 1.24 (m, 6H), 0.89 (t, 3H, *J* = 6.6 Hz); ^{13}C NMR (101 MHz, $CDCl_3$) δ 172.1, 139.6, 128.9, 128.7, 126.9, 74.1, 39.7, 31.4, 29.4, 26.4, 22.5, 14.0; HRMS (ES$^+$, TOF) *m/z*: $(M+H)^+$ calcd. for $C_{14}H_{22}O_2N$ 236.1651; Found 236.1657.

N,N-Diethyl-2-phenylacetamide (30)[15]

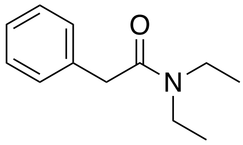

Following General Procedure A gave amide **30** (0.19 g, 1.0 mmol, 10%) as a colourless oil: ATR-FTIR ν_{max}/cm^{-1} (neat) 1630; ^{1}H NMR (400 MHz, $CDCl_3$) δ 7.34 - 7.26 (m, 5H), 3.74 (s, 2H) 3.43 - 3.32 (m, 4H),

1.17 - 1.10 (m, 6H); ^{13}C NMR (101 MHz, $CDCl_3$) δ 170.2, 135.5, 128.7, 128.6, 126.7, 42.4, 40.9, 40.2, 14.2, 12.9; HRMS (ES^+, TOF) *m/z*: $(M+H)^+$ calcd. for $C_{12}H_{18}ON$ 192.1388; Found 192.1393.

_N_-Phenyl-2-(thiophen-2-yl)acetamide (31)[16]

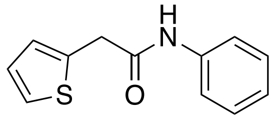

Following General Procedure B on a 45 mmol scale with fractional distillation of MeOH gave amide **31** (7.73 g, 35.6 mmol, 79%) as a white solid: m.p. 113.7 – 115.8 ˚C (lit.[16] 114-116 ˚C); ATR-FTIR ν_{max}/cm^{-1} (neat) 3254, 1656; NMR (400 MHz, $CDCl_3$) δ 7.56 (br s, 1H), 7.48 (d, 2H, J = 7.9 Hz), 7.34 - 7.29 (m, 3H), 7.15 - 7.11 (t, 1H, J = 7.3 Hz), 7.08 - 7.06 (m, 2H), 3.96 (s, 2H); ^{13}C NMR (101 MHz, $CDCl_3$) δ 168.0, 137.5, 135.7, 129.0, 127.8, 127.6, 126.0, 124.7, 120.0, 38.5; HRMS (ES^+, TOF) *m/z*: $(M+H)^+$ calcd. for $C_{12}H_{12}ONS$ 218.0640; Found 218.0634.

Benzyl ((_S_)-1-oxo-1-(((_S_)-1-phenylethyl)amino)propan-2-yl)carbamate (32)[1]

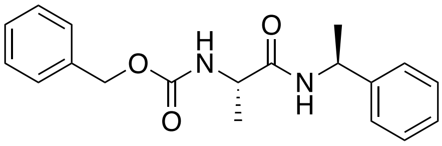

Following General Procedure B gave amide **32** (0.98 g, 3.0 mmol, 30%) as a white solid: m.p. 150.8 – 151.9 ˚C (lit.[1] 150-151 ˚C); $[\alpha]_D^{26.4}$= -54.6 (*c* 0.26, $CHCl_3$); ATR-FTIR ν_{max}/cm^{-1} (neat) 3291, 1682, 1643; ^{1}H NMR (400 MHz, $CDCl_3$) δ 7.36 - 7.29 (m, 10H), 6.52 (br s, 1H), 5.47 (br s, 1H), 5.09 (m, 3H), 4.28 (br s, 1H), 1.48 - 1.47 (d, 3H, J = 6.9 Hz), 1.39 - 1.37 (d, 3H, J = 6.9 Hz); ^{13}C NMR (101 MHz, $CDCl_3$) δ 171.3, 156.0, 142.9, 136.2, 128.7, 128.6, 128.3, 128.1, 127.4, 126.0, 67.0, 50.6, 48.9, 21.8, 18.6; HRMS (ES^+, TOF) *m/z*: $(M+H)^+$ calcd. for $C_{19}H_{23}O_3N_2$ 327.1709; Found 327.1700. Following General Procedure A gave amide **32** (1.01 g, 3.1 mmol, 31%) as a single diastereoisomer.

^{1}H NMR spectrum of *N*-(4-Methylbenzyl)-2-phenylacetamide (**3**) (400 MHz, $CDCl_3$)

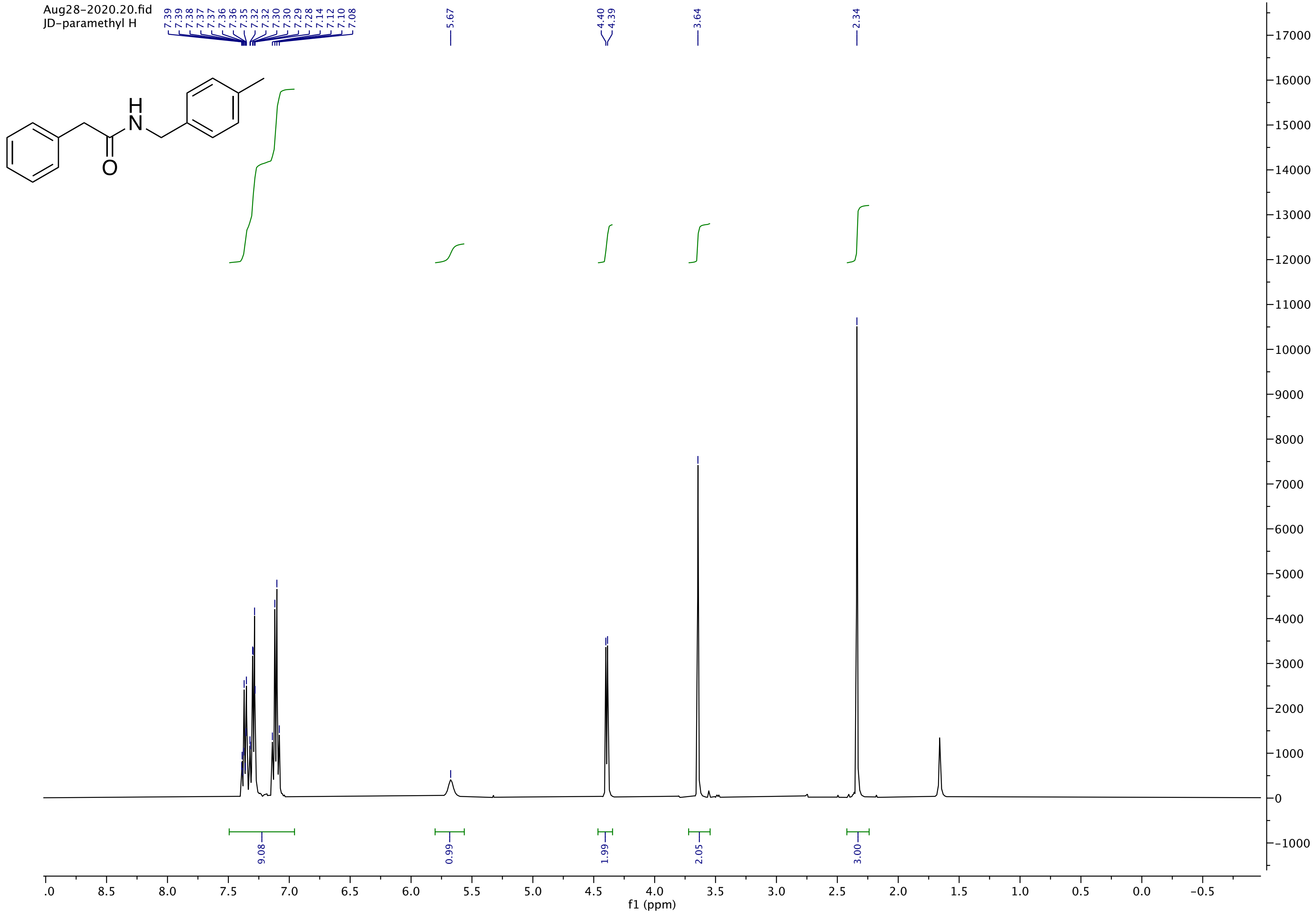

^{13}C NMR spectrum of *N*-(4-Methylbenzyl)-2-phenylacetamide (**3**) (101 MHz, $CDCl_3$)

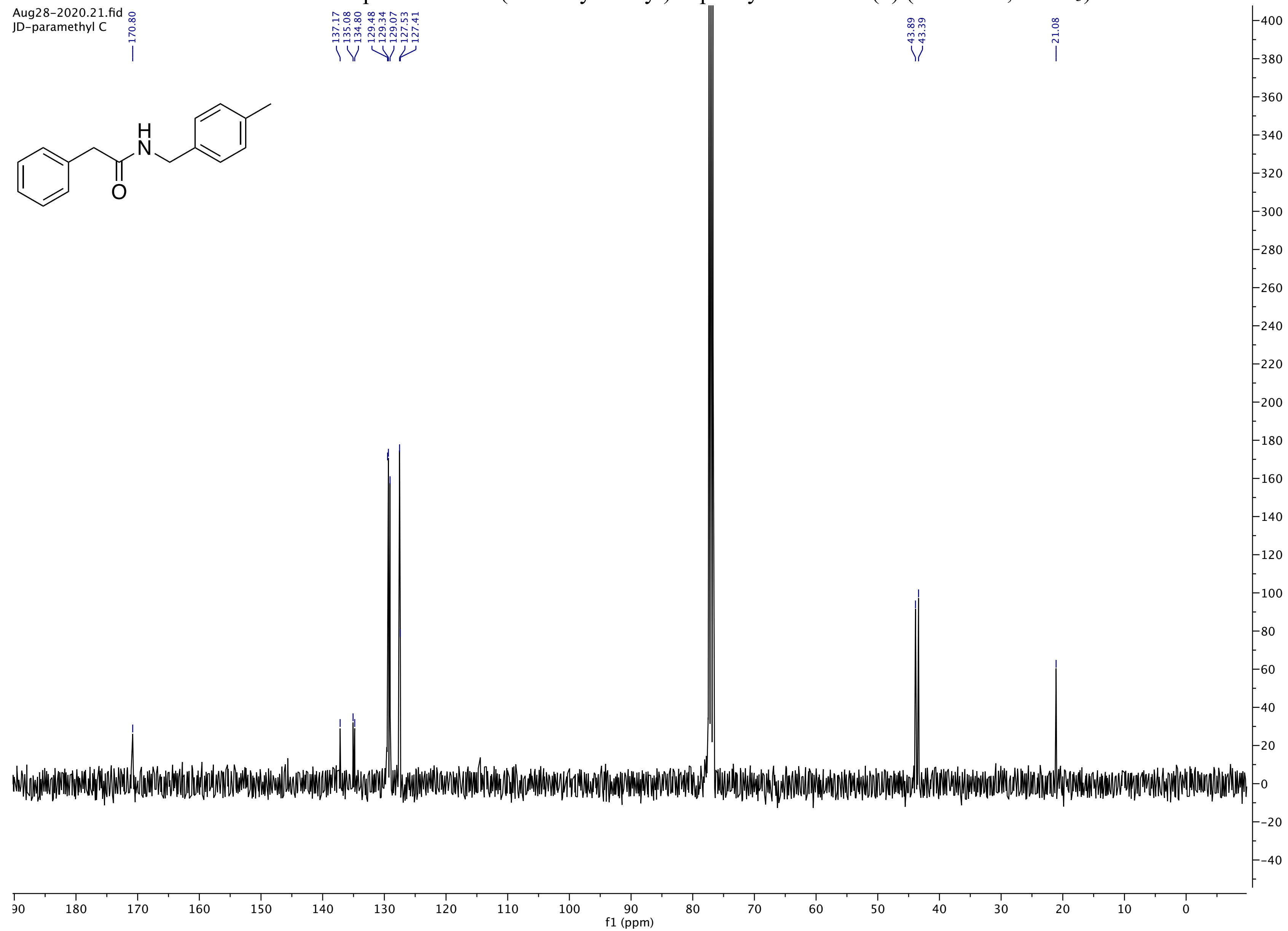

^{1}H NMR spectrum of 1-morpholino-2-phenylethan-1-one (**4**) (400 MHz, $CDCl_3$)

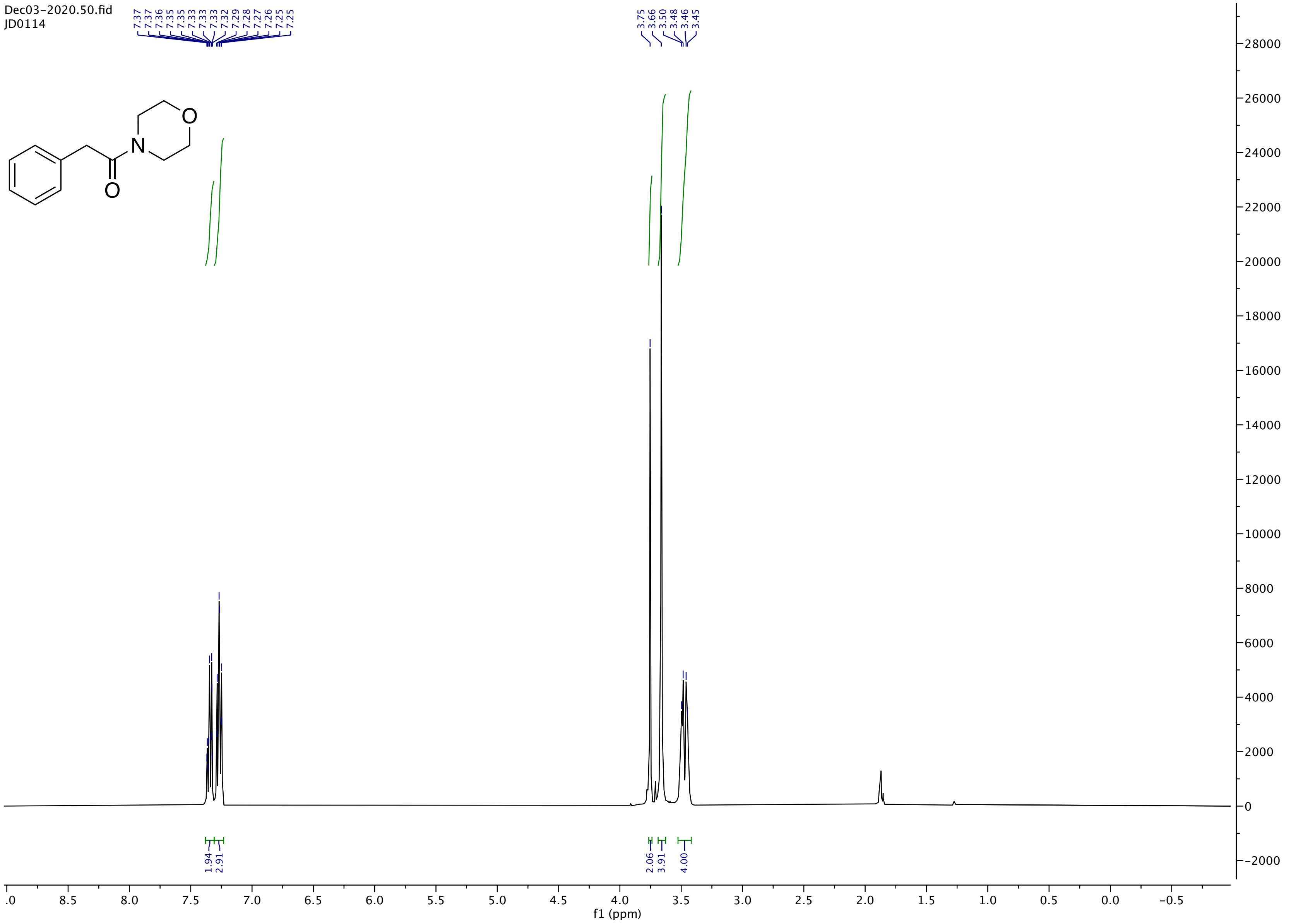

^{13}C NMR spectrum of 1-morpholino-2-phenylethan-1-one (**4**) (101 MHz, $CDCl_3$)

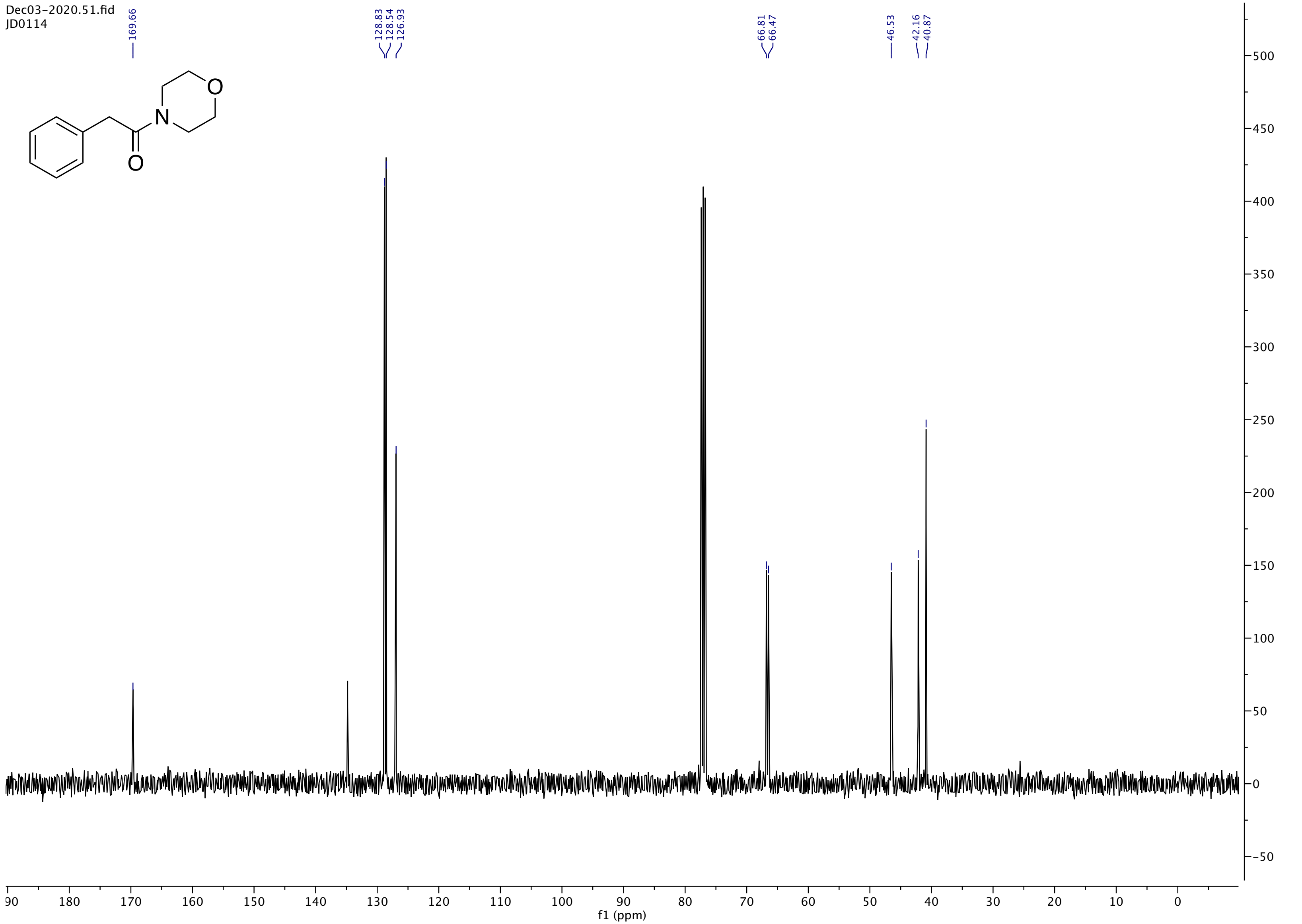

^{1}H NMR spectrum of *N*-benzyl-*N*-methyl-2-phenylacetamide (**5**) (400 MHz, $CDCl_3$)

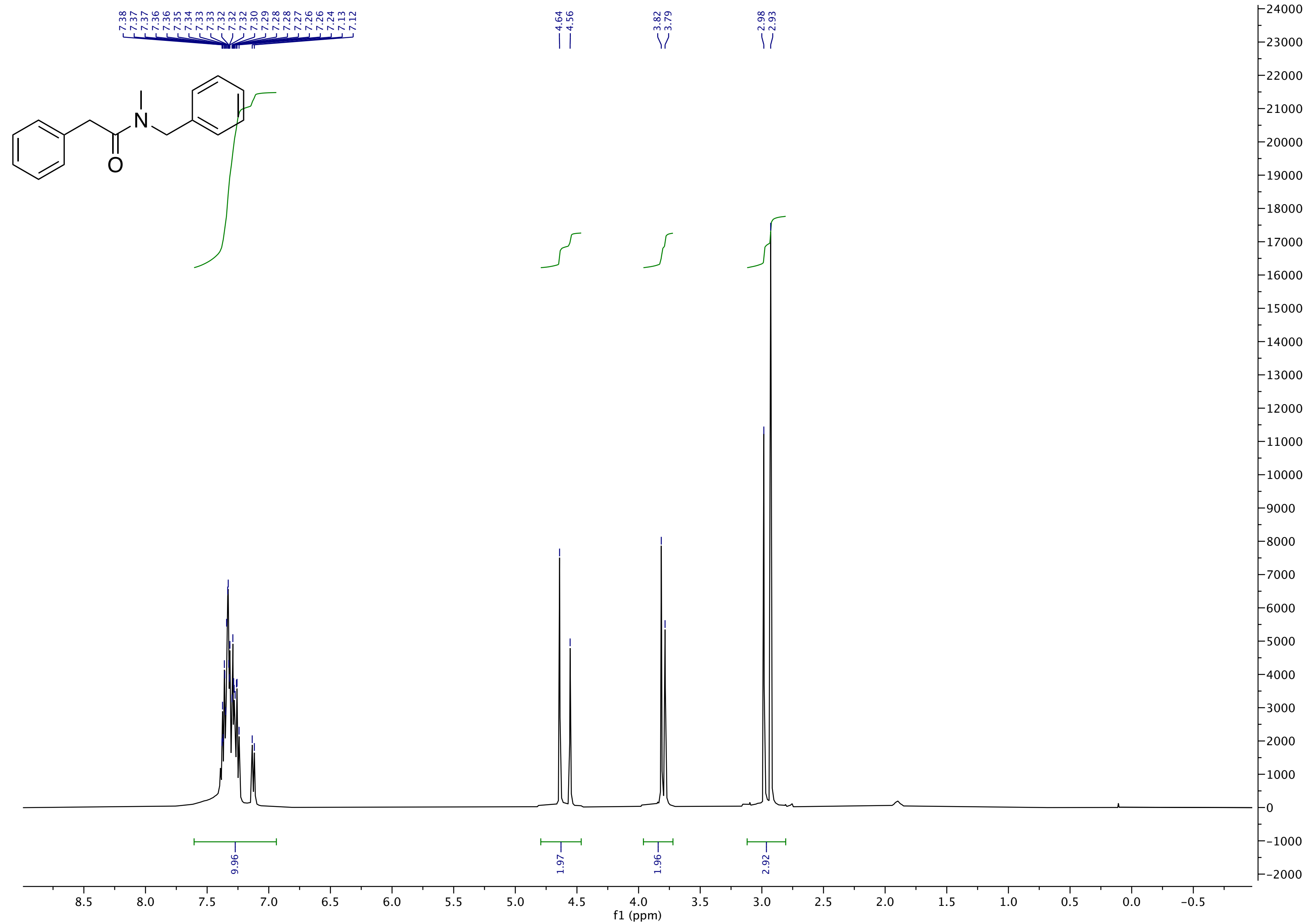

^{13}C NMR spectrum of *N*-benzyl-*N*-methyl-2-phenylacetamide (**5**) (101 MHz, $CDCl_3$)

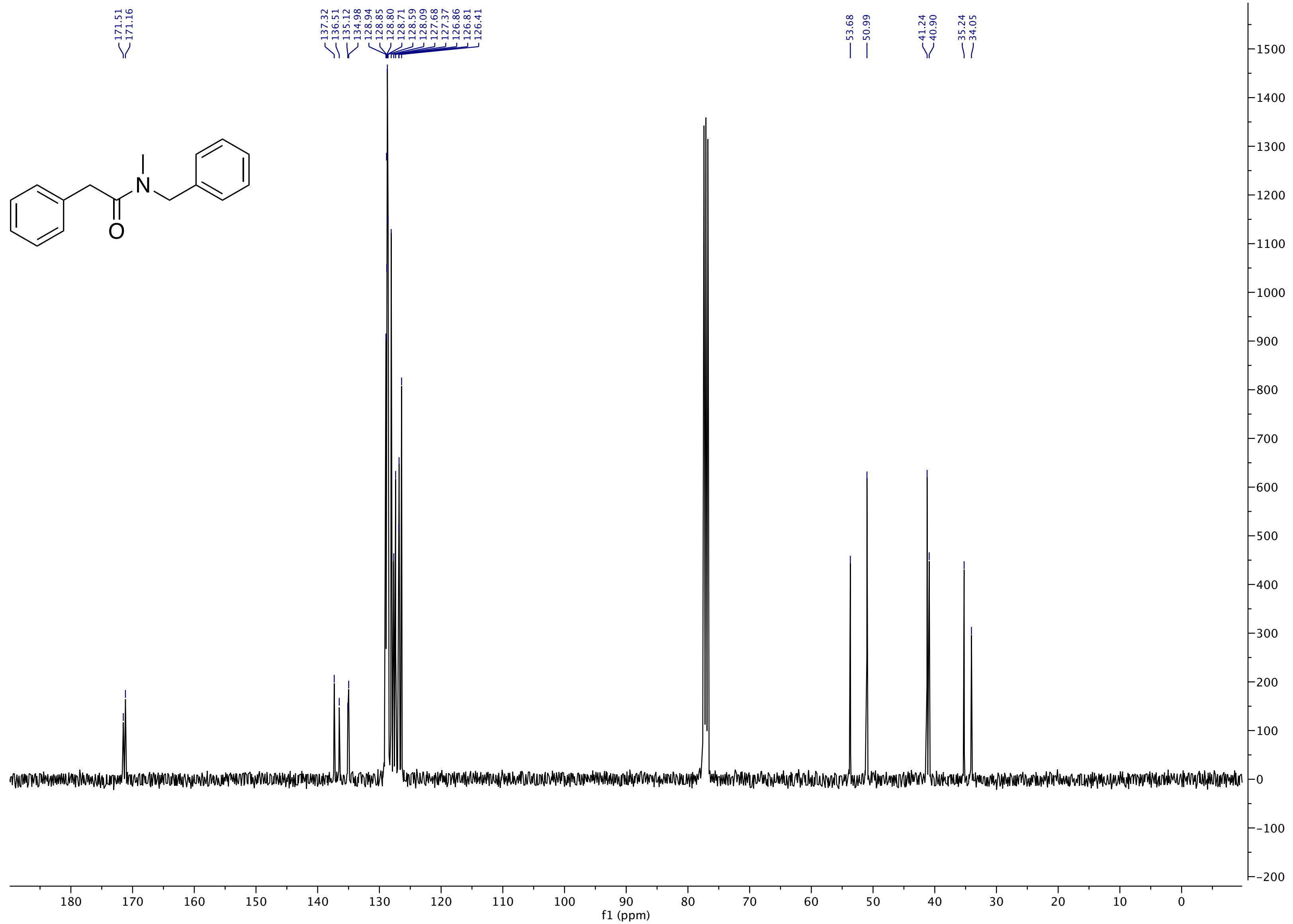

^{1}H NMR spectrum of *N*-(4-methylbenzyl)benzamide (**6**) (400 MHz, $CDCl_3$)

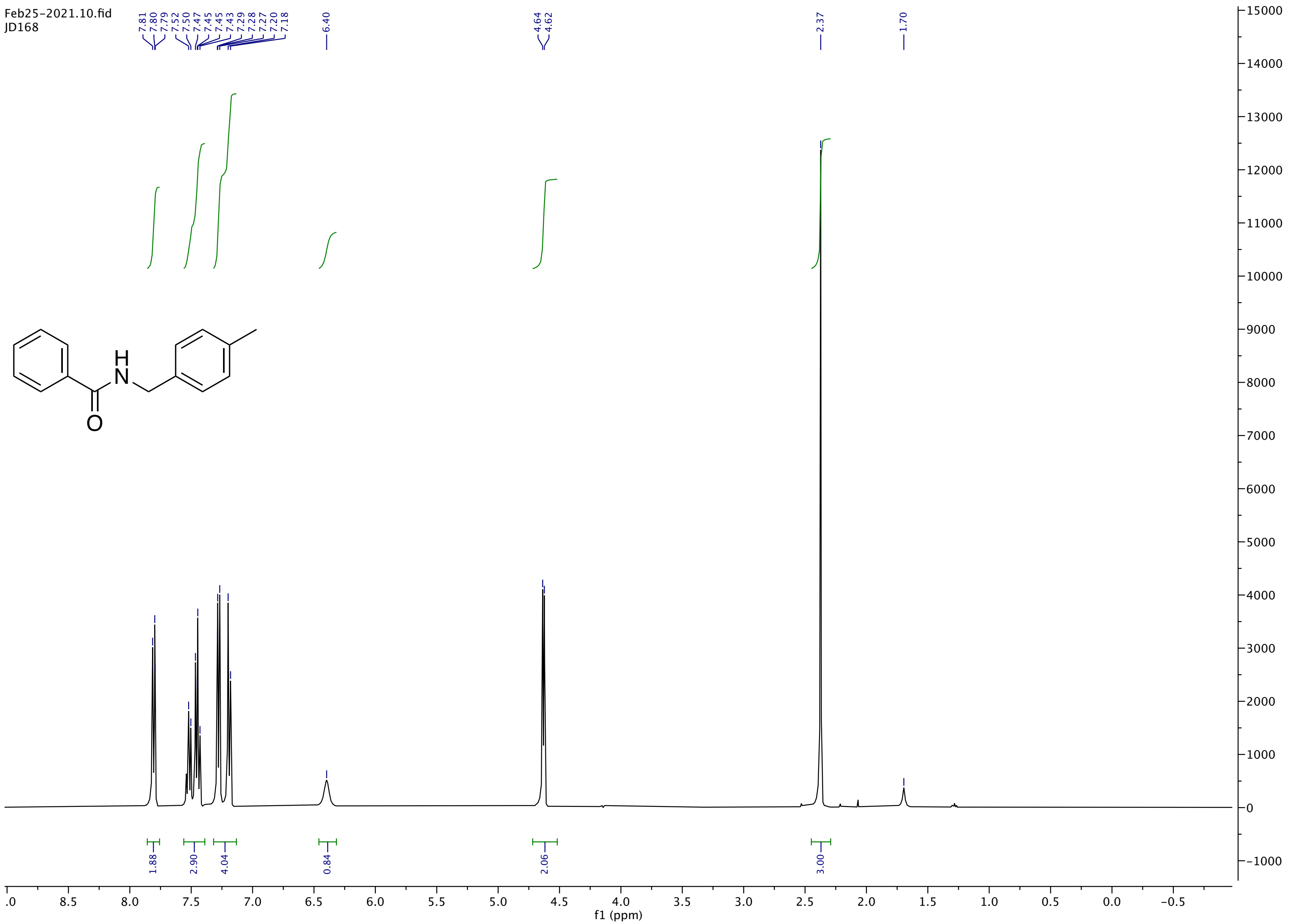

^{13}C NMR spectrum of *N*-(4-methylbenzyl)benzamide (**6**) (101 MHz, $CDCl_3$)

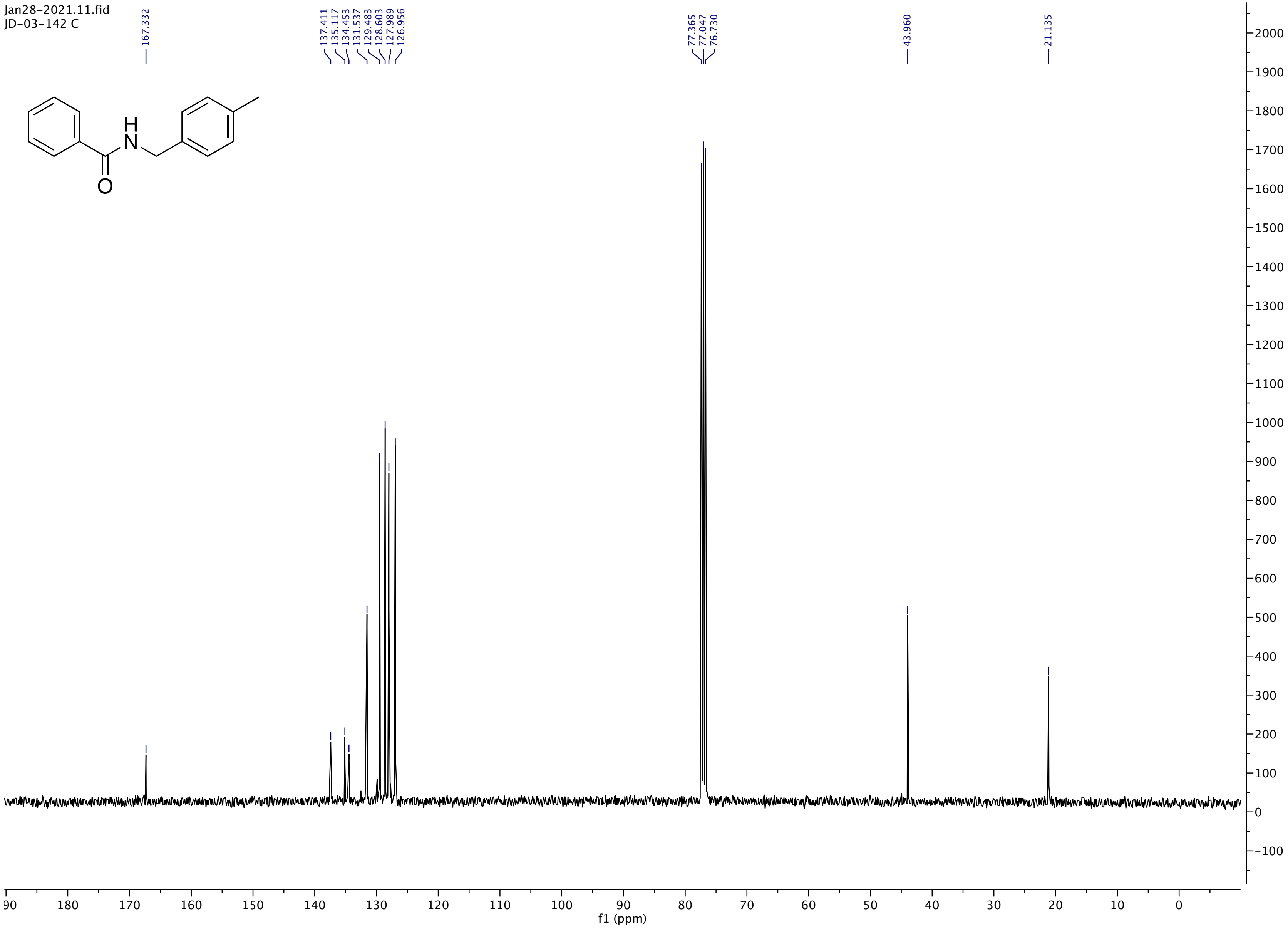

^{1}H NMR spectrum of morpholino(phenyl)methanone (**7**) (400 MHz, $CDCl_3$)

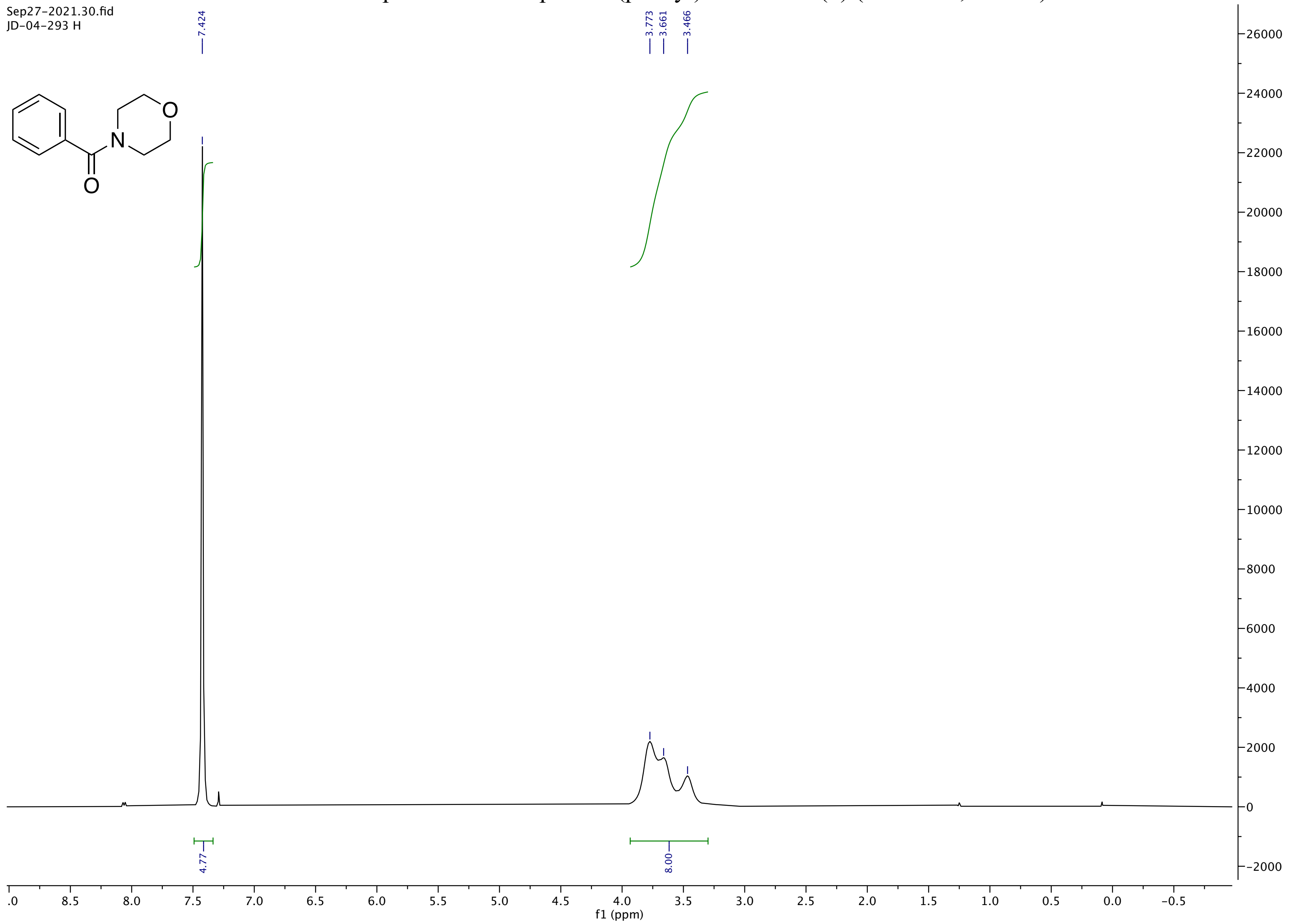

^{13}C NMR spectrum of morpholino(phenyl)methanone (**7**) (101 MHz, $CDCl_3$)

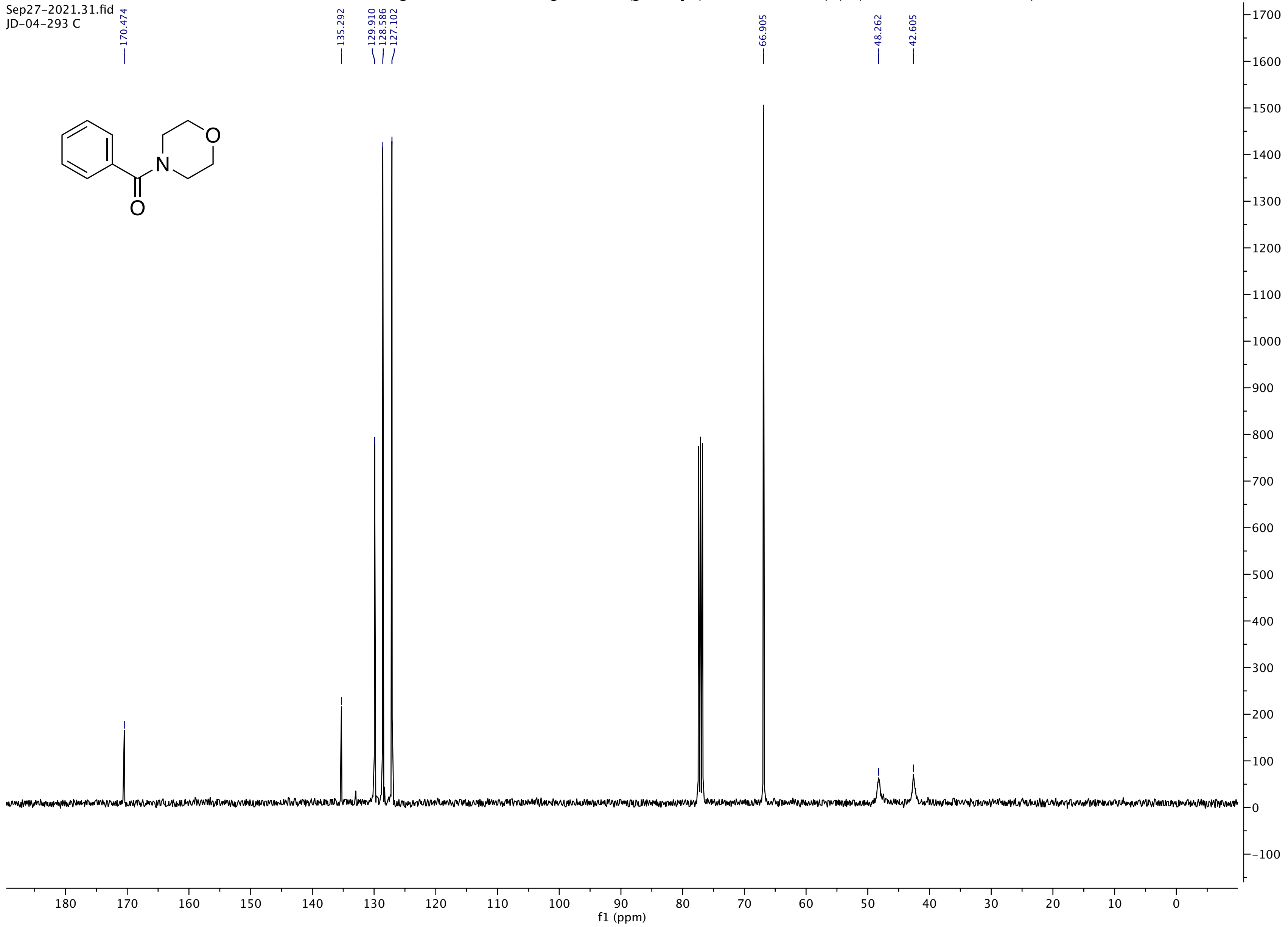

^{1}H NMR spectrum of *N*-(4-methylbenzyl)benzamide (**8**) (400 MHz, $CDCl_3$)

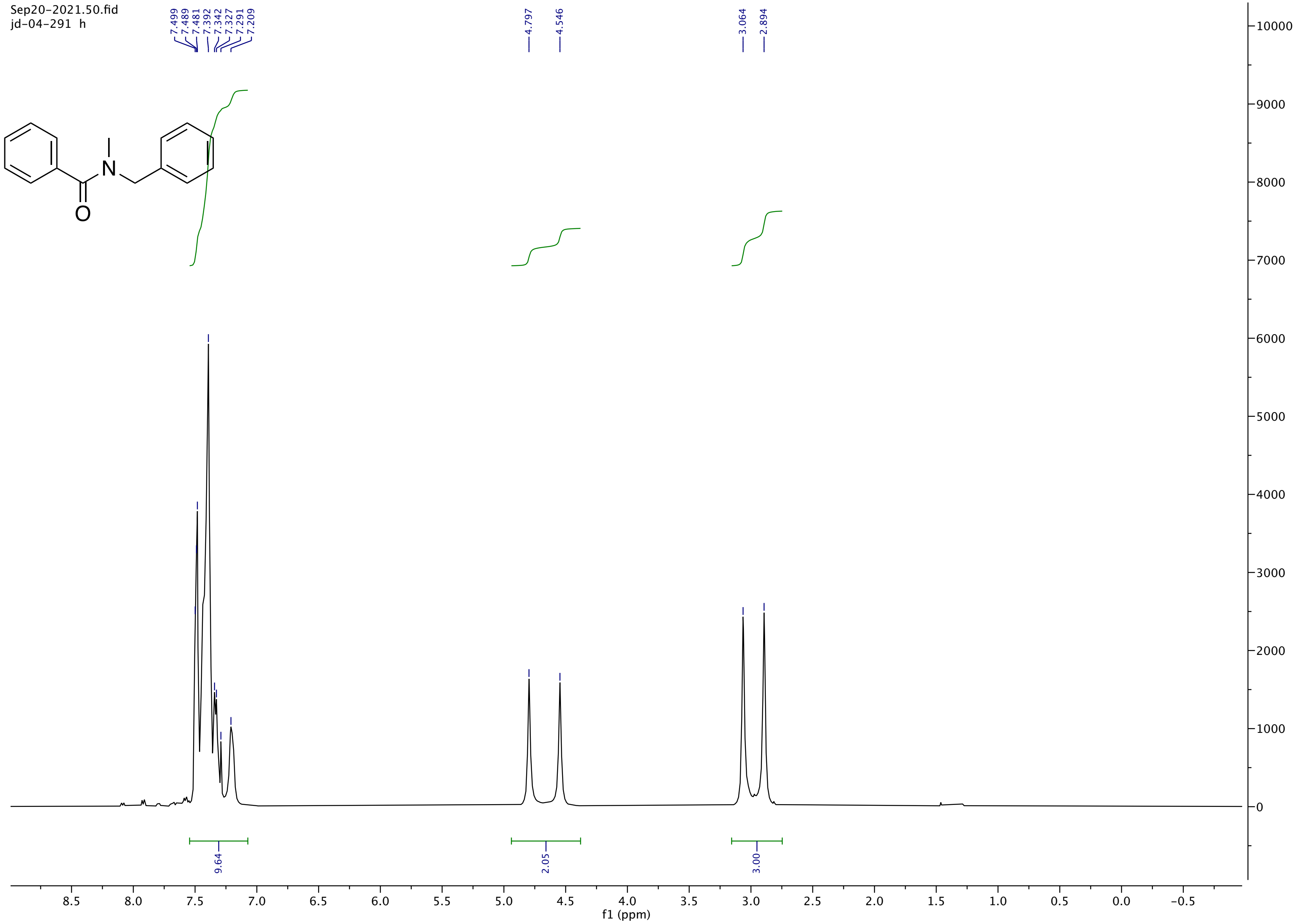

^{13}C NMR spectrum of *N*-(4-methylbenzyl)benzamide (**8**) (101 MHz, $CDCl_3$)

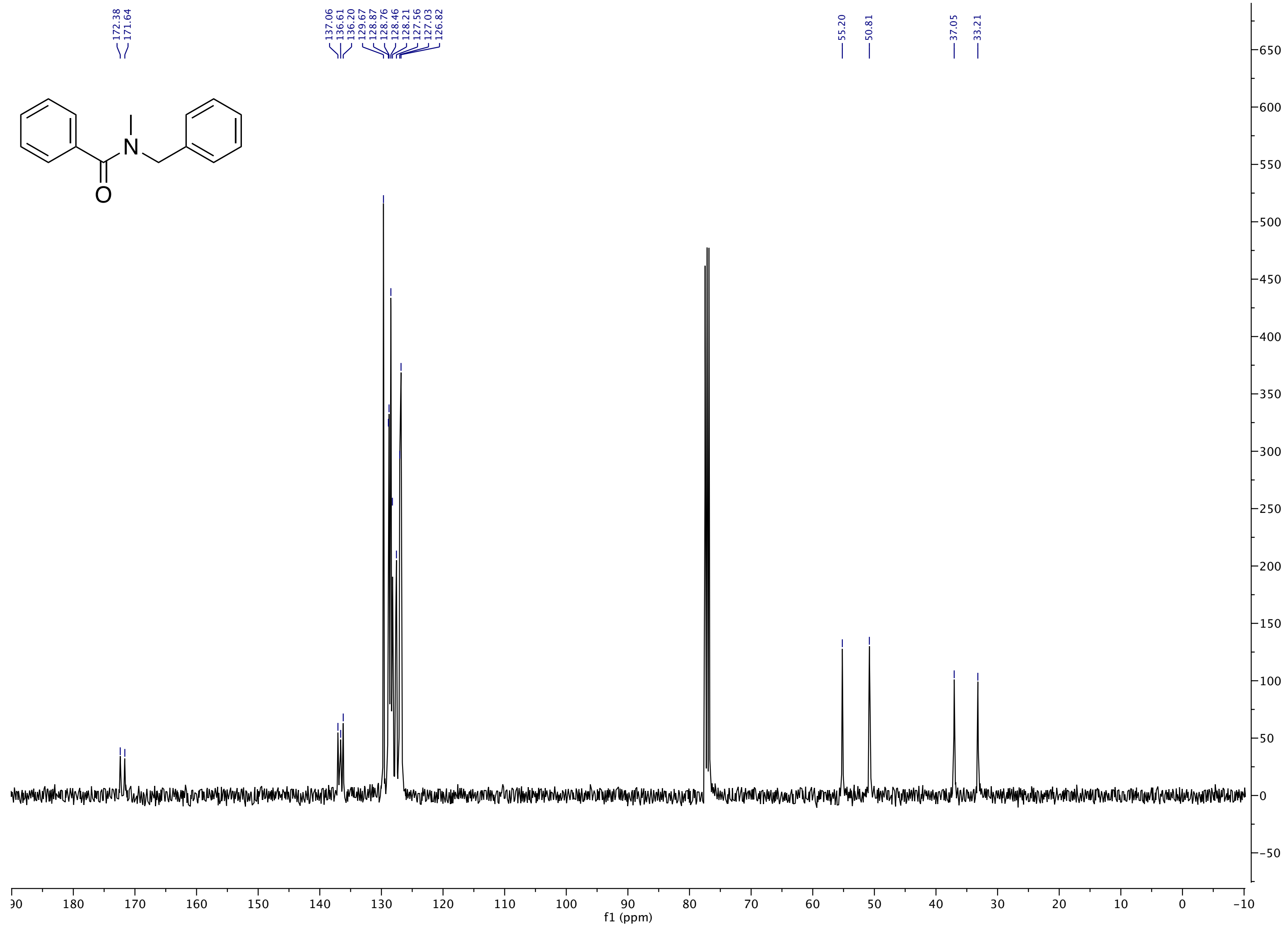

^{1}H NMR spectrum of *N*,2-diphenylacetamide (**9**) (400 MHz, $CDCl_3$)

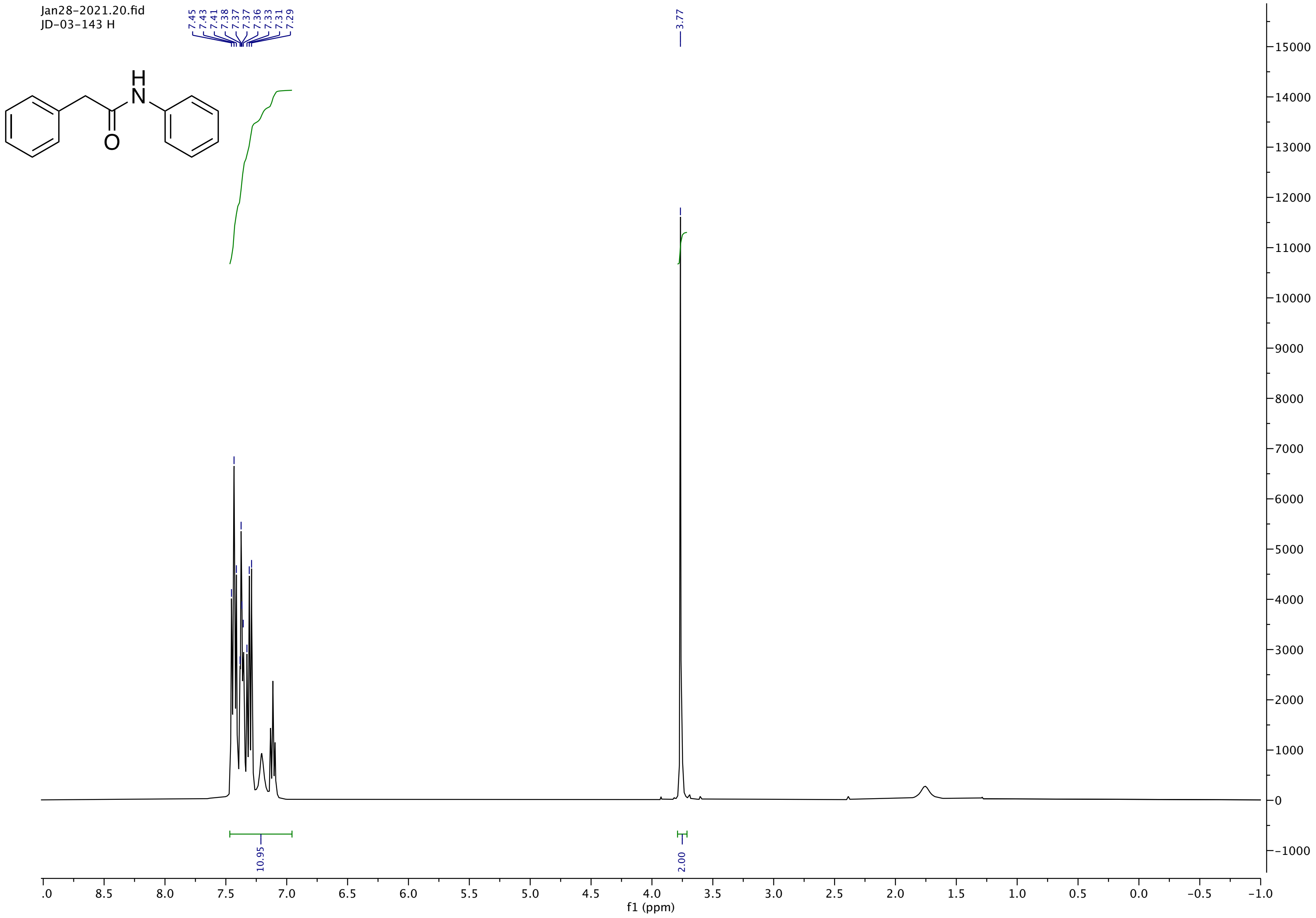

^{13}C NMR spectrum of *N*,2-diphenylacetamide (**9**) (101 MHz, $CDCl_3$)

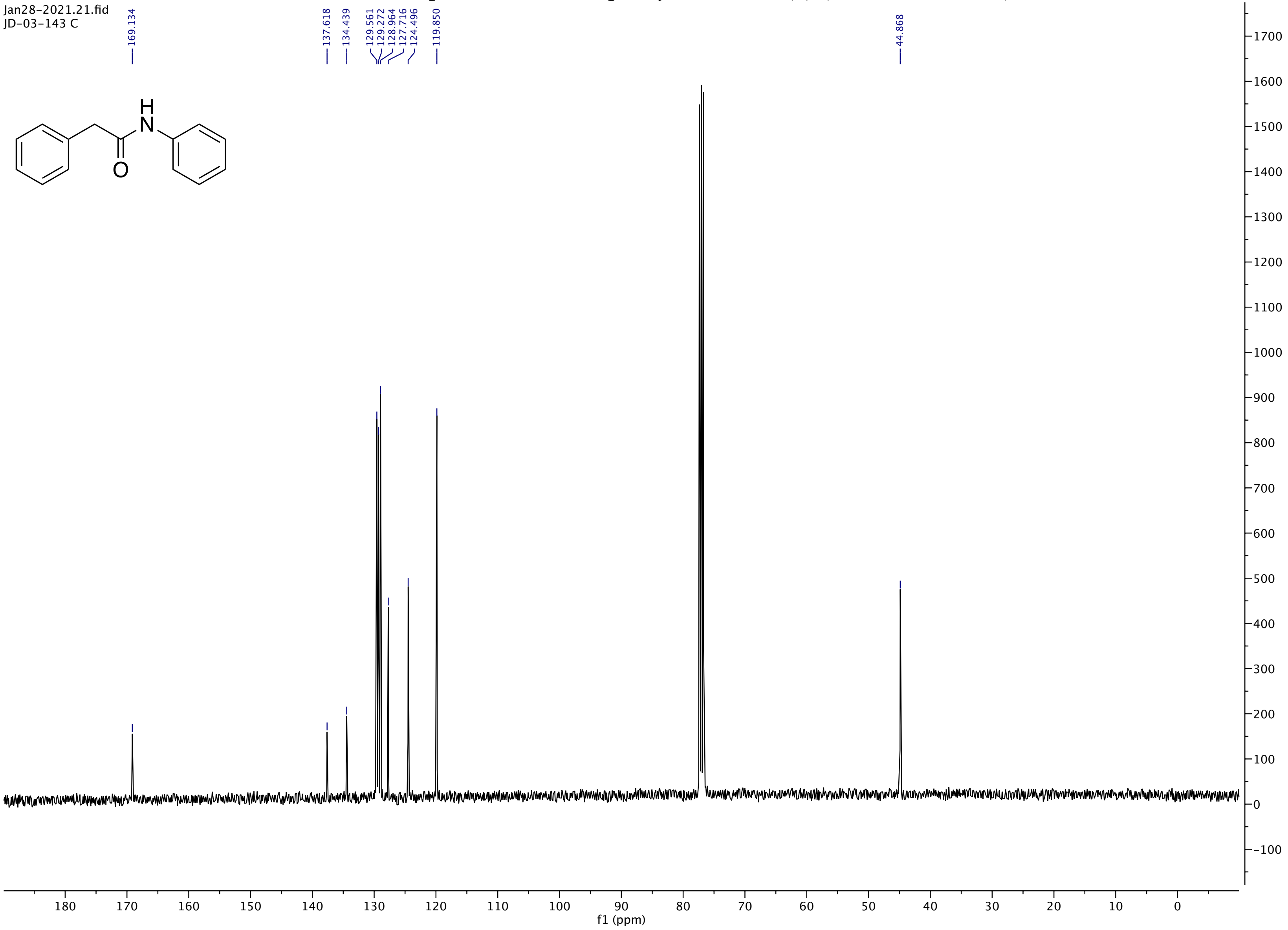

^{1}H NMR spectrum of *N*-phenylbenzamide (**10**) (400 MHz, $CDCl_3$)

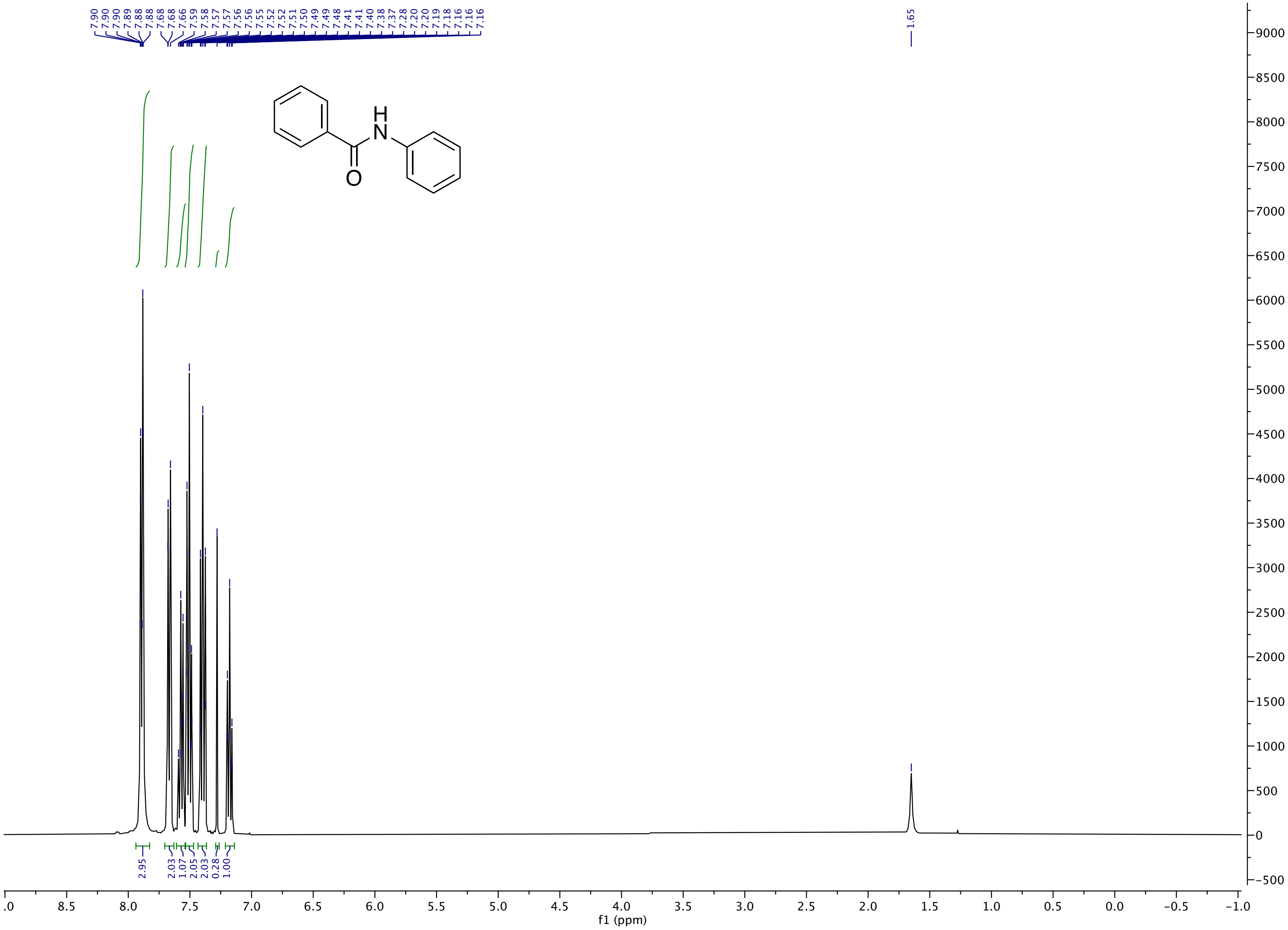

^{13}C NMR spectrum of *N*-phenylbenzamide (**10**) (101 MHz, $CDCl_3$)

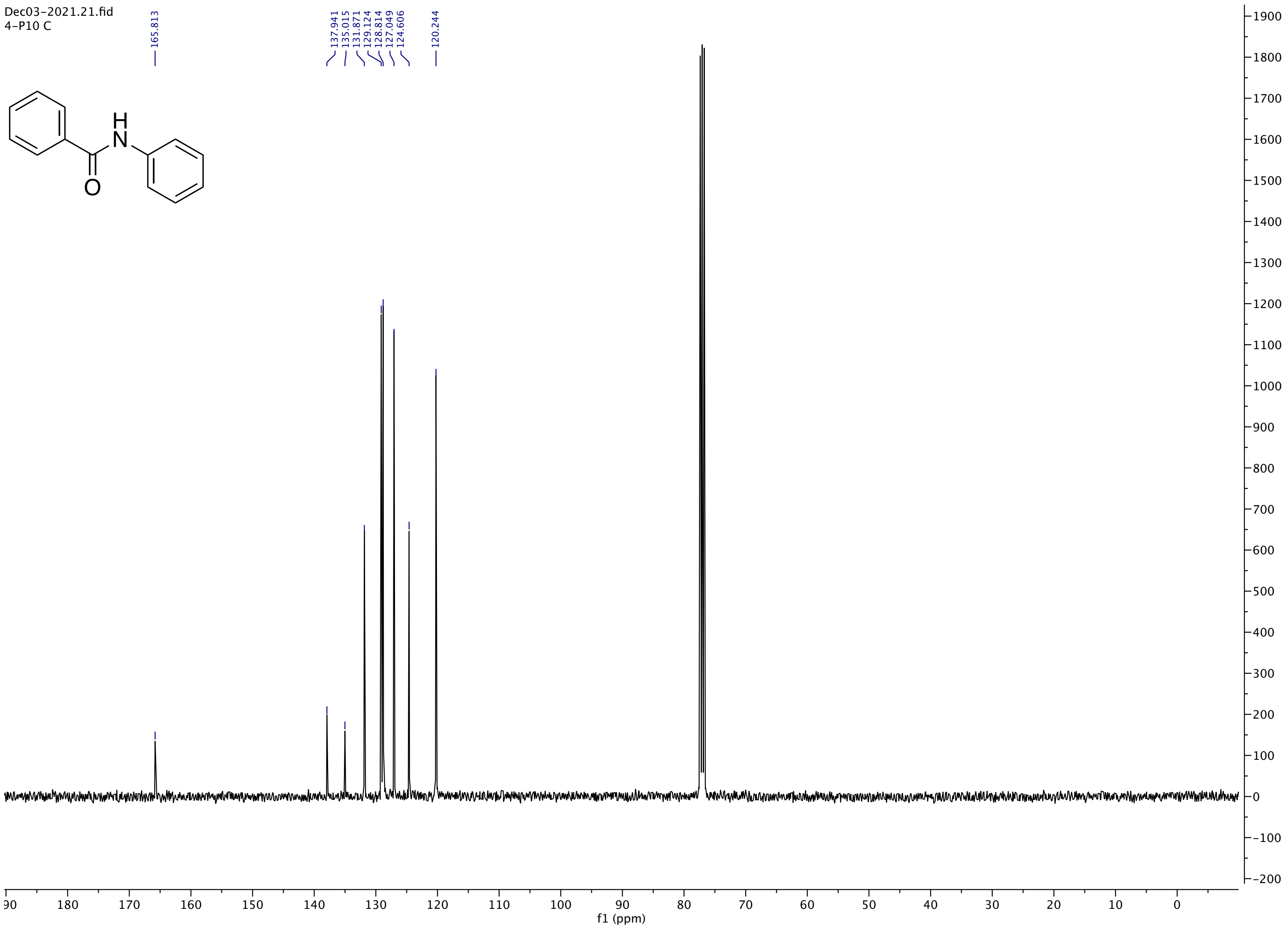

^{1}H NMR spectrum of *N*-benzyl-2-phenylacetamide (**11**) (400 MHz, $CDCl_3$)

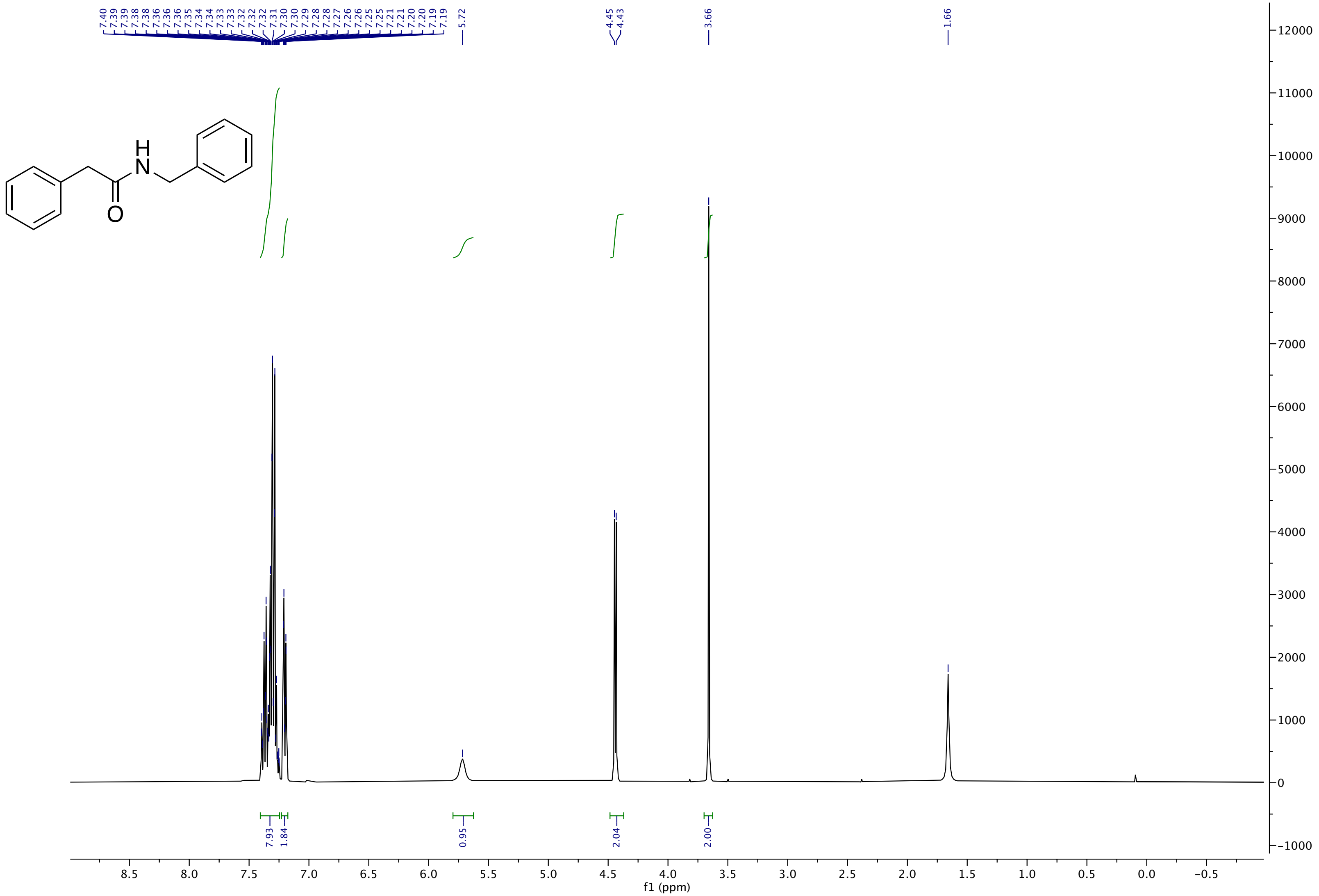

^{13}C NMR spectrum of *N*-benzyl-2-phenylacetamide (**11**) (101 MHz, $CDCl_3$)

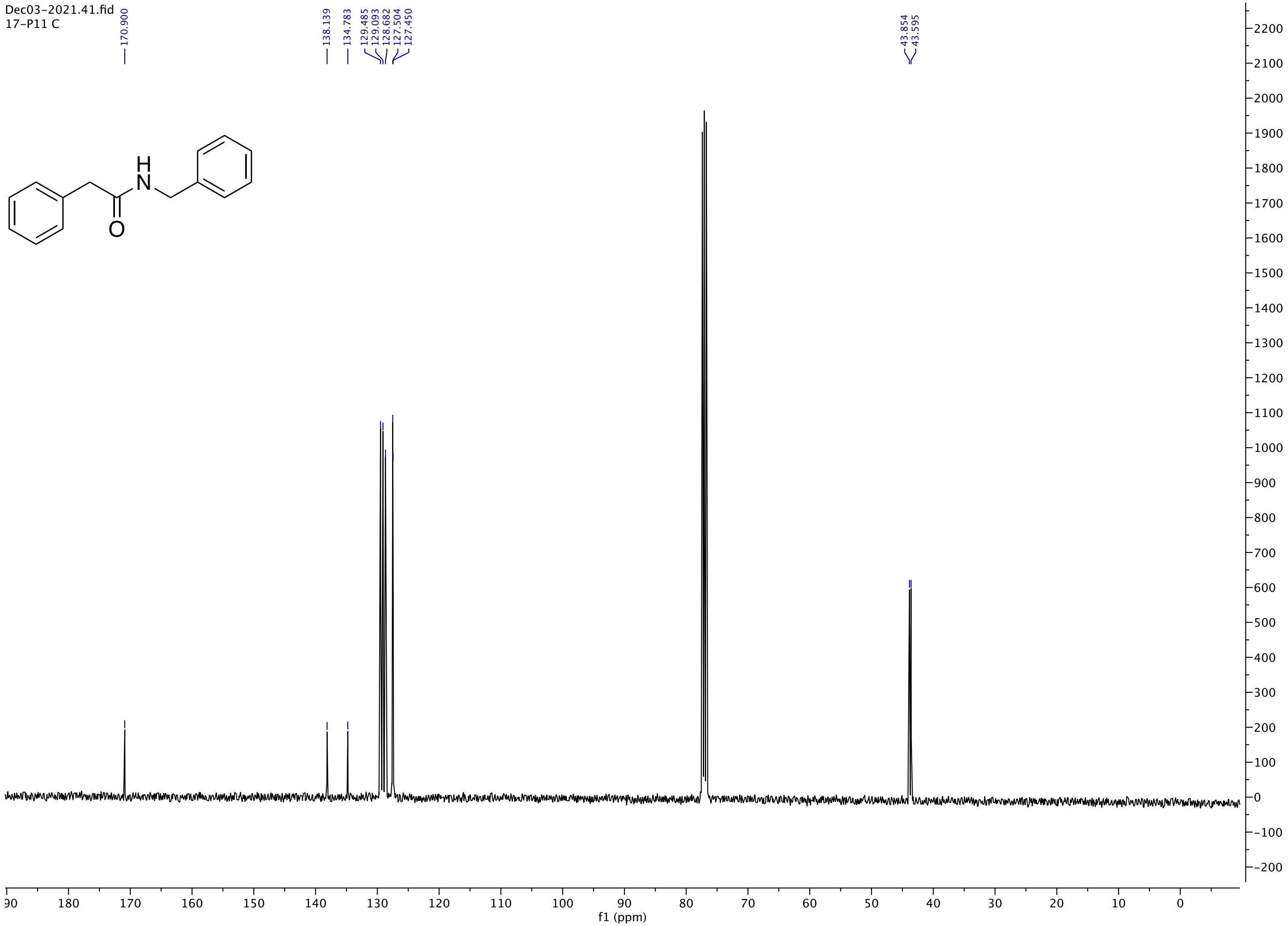

^{1}H NMR spectrum of *N*-(4-methylbenzyl)-2-(thiophen-2-yl)acetamide (**12**) (400 MHz, $CDCl_3$)

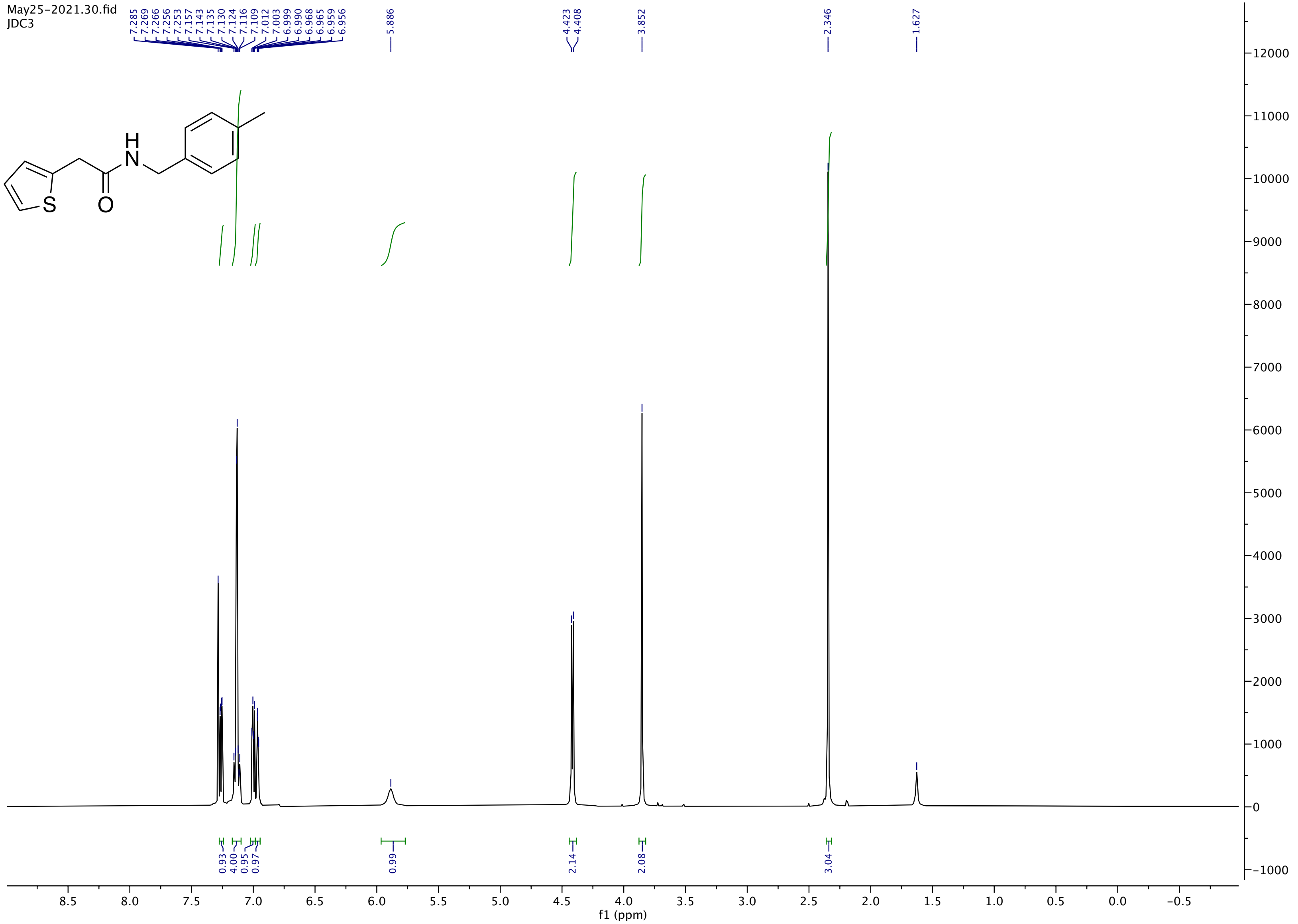

^{13}C NMR spectrum of *N*-(4-methylbenzyl)-2-(thiophen-2-yl)acetamide (**12**) (101 MHz, $CDCl_3$)

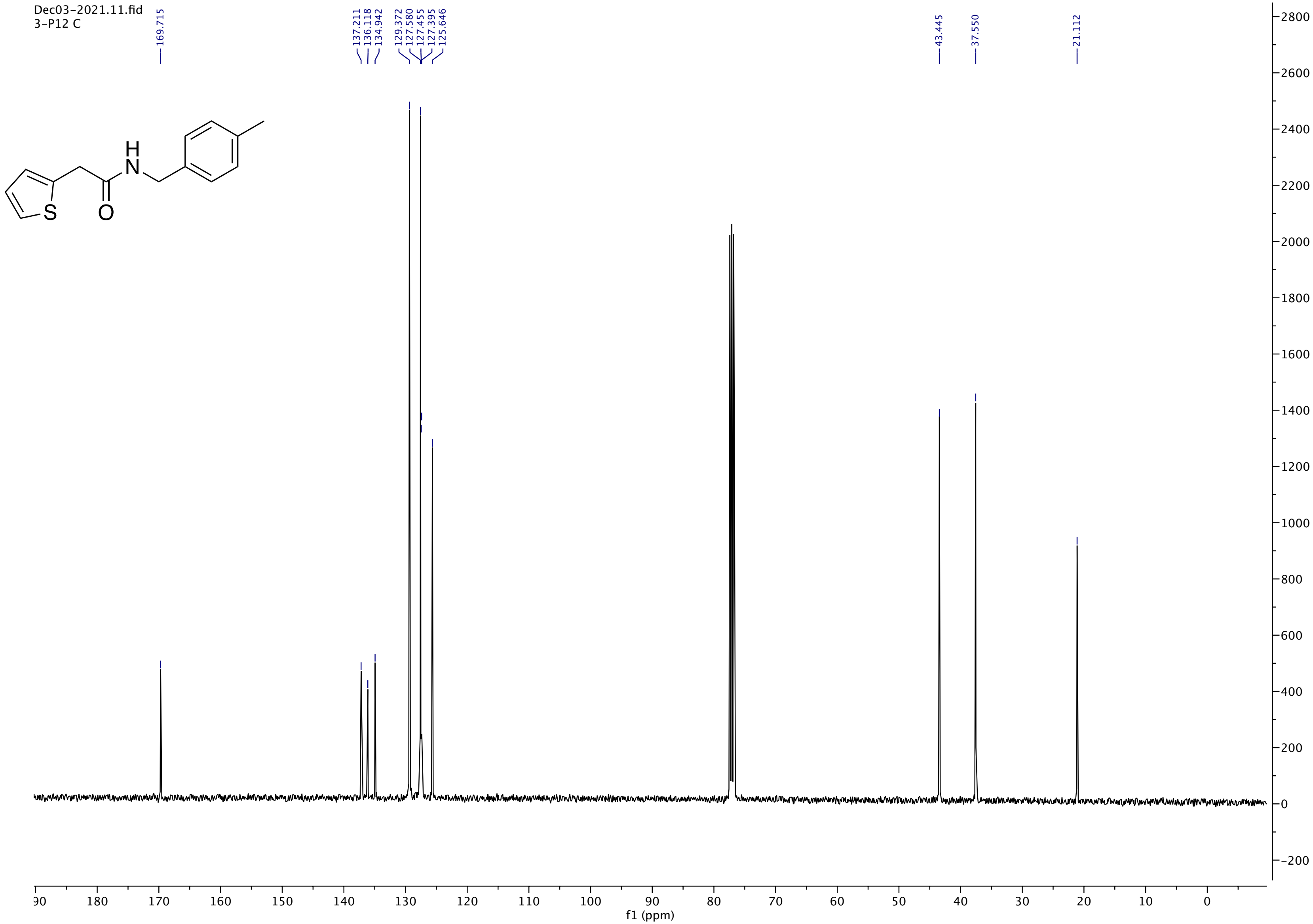

^{1}H NMR spectrum of 2-phenyl-1-(piperidin-1-yl)ethan-1-one (**13**) (400 MHz, $CDCl_3$)

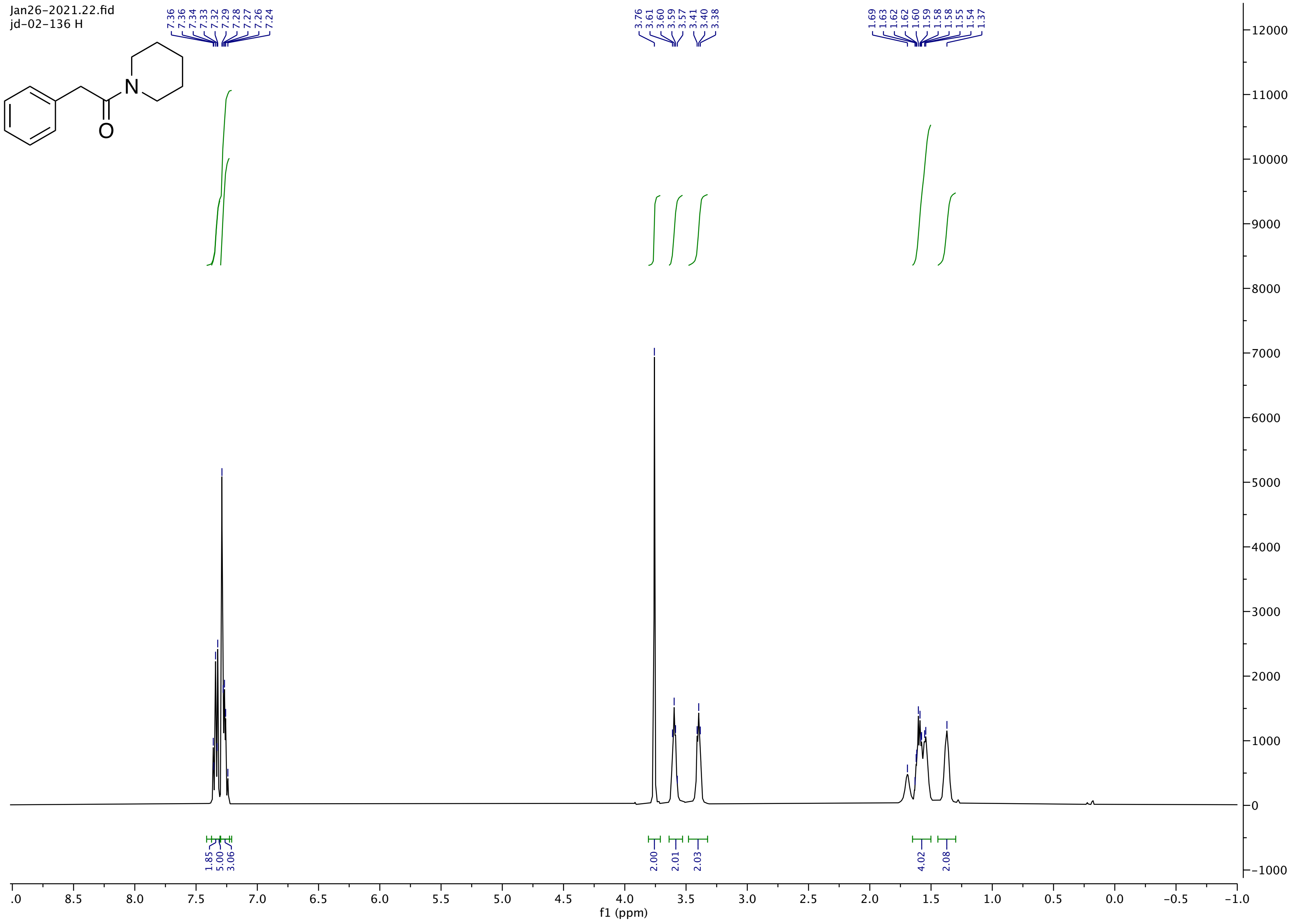

^{13}C NMR spectrum of 2-phenyl-1-(piperidin-1-yl)ethan-1-one (**13**) (101 MHz, $CDCl_3$)

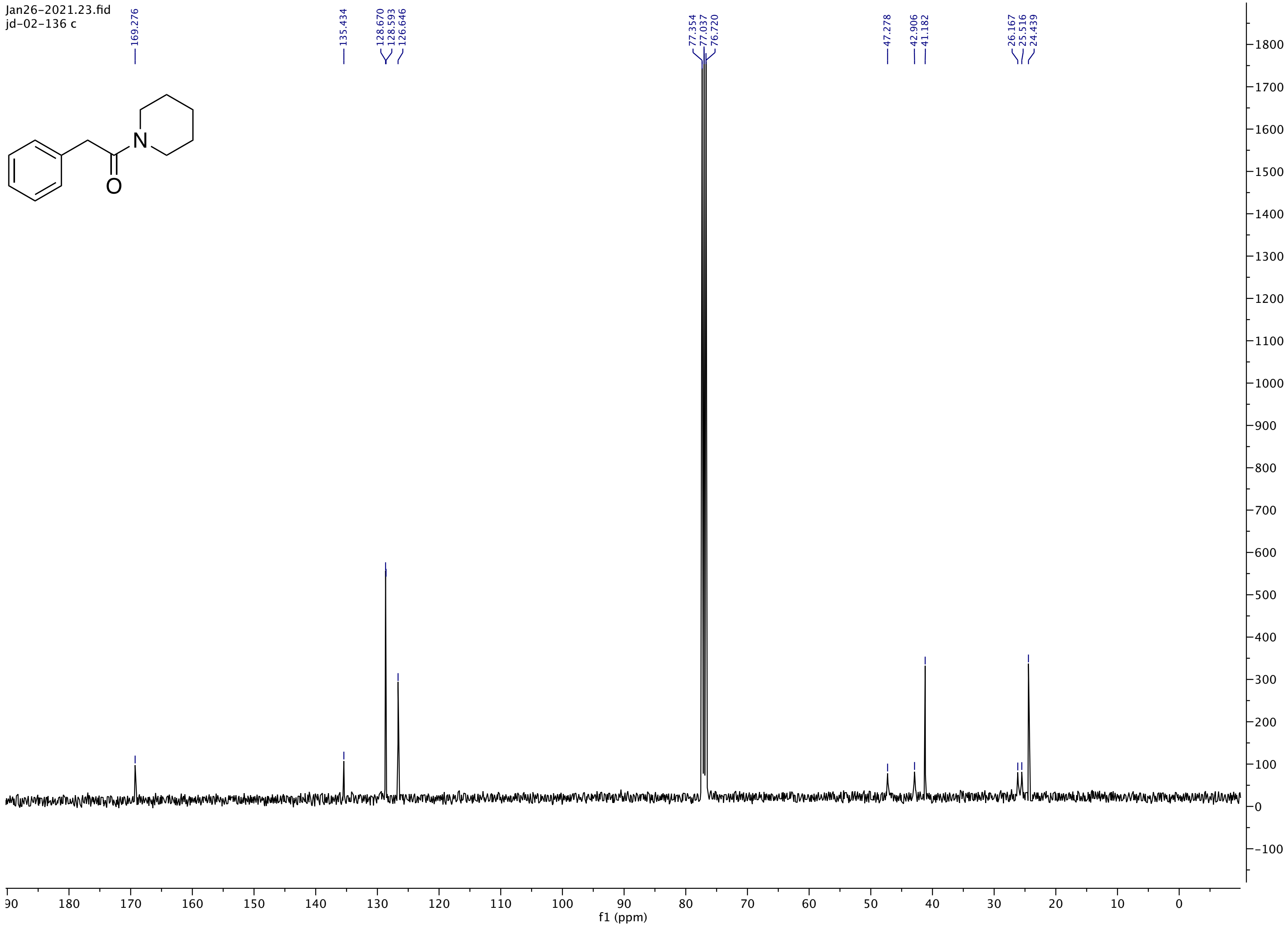

^{1}H NMR spectrum of *N*-cycloheptylcyclohexanecarboxamide (**14**) (400 MHz, $CDCl_3$)

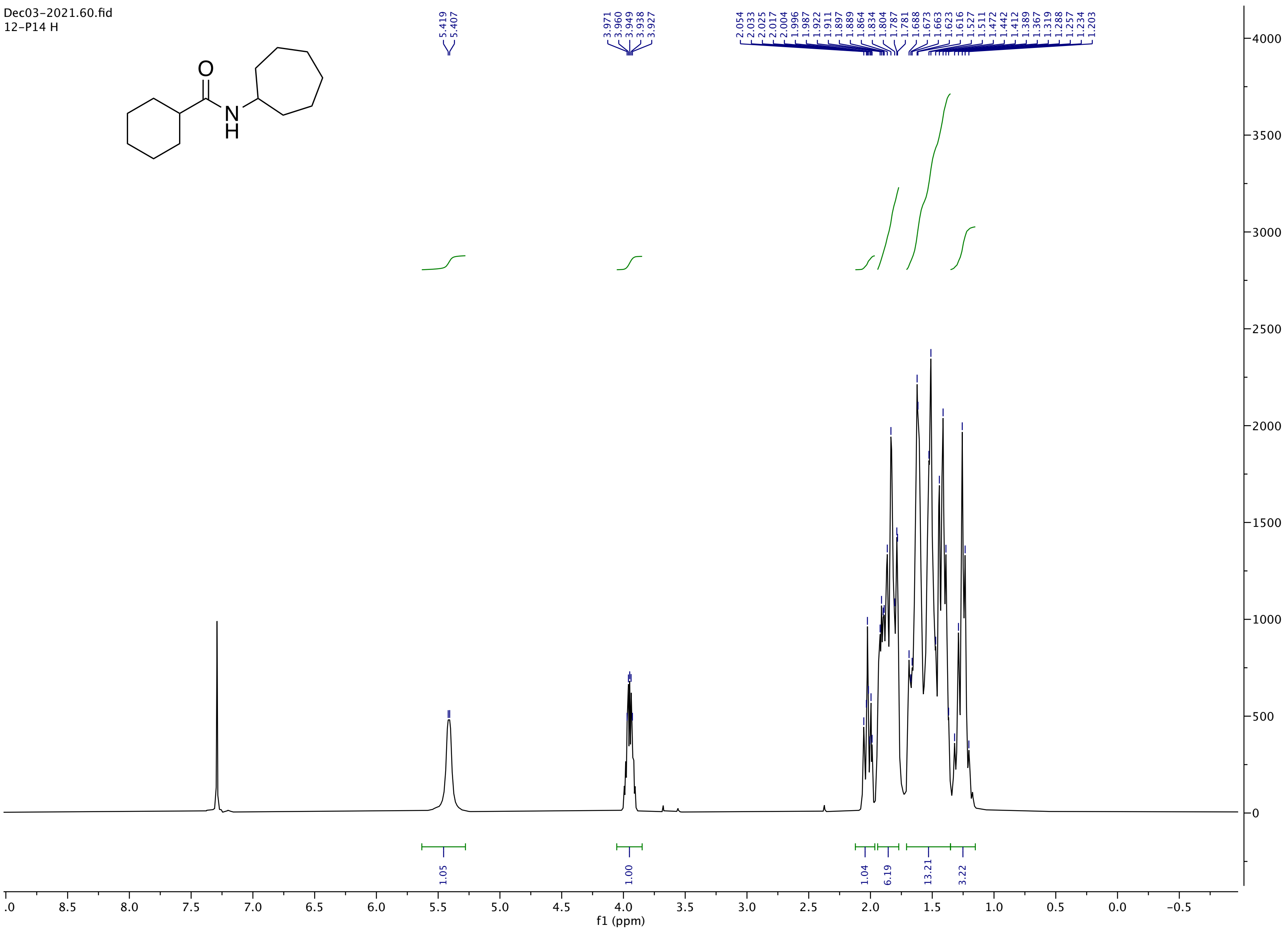

^{13}C NMR spectrum of *N*-cycloheptylcyclohexanecarboxamide (**14**) (101 MHz, $CDCl_3$)

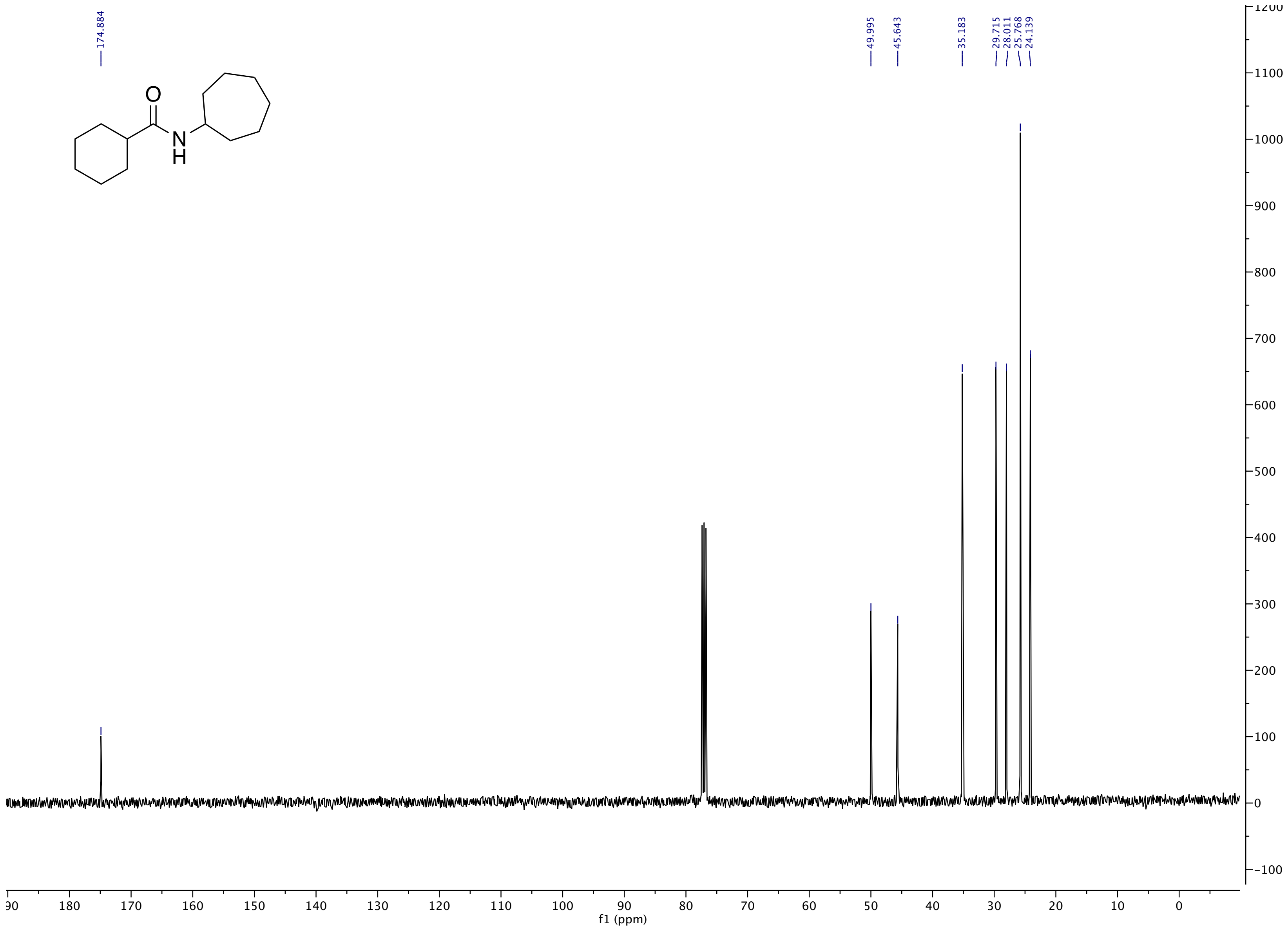

^{1}H NMR spectrum of (*S*)-*N*-(1-phenylethyl)pivalamide (**15**) (400 MHz, $CDCl_3$)

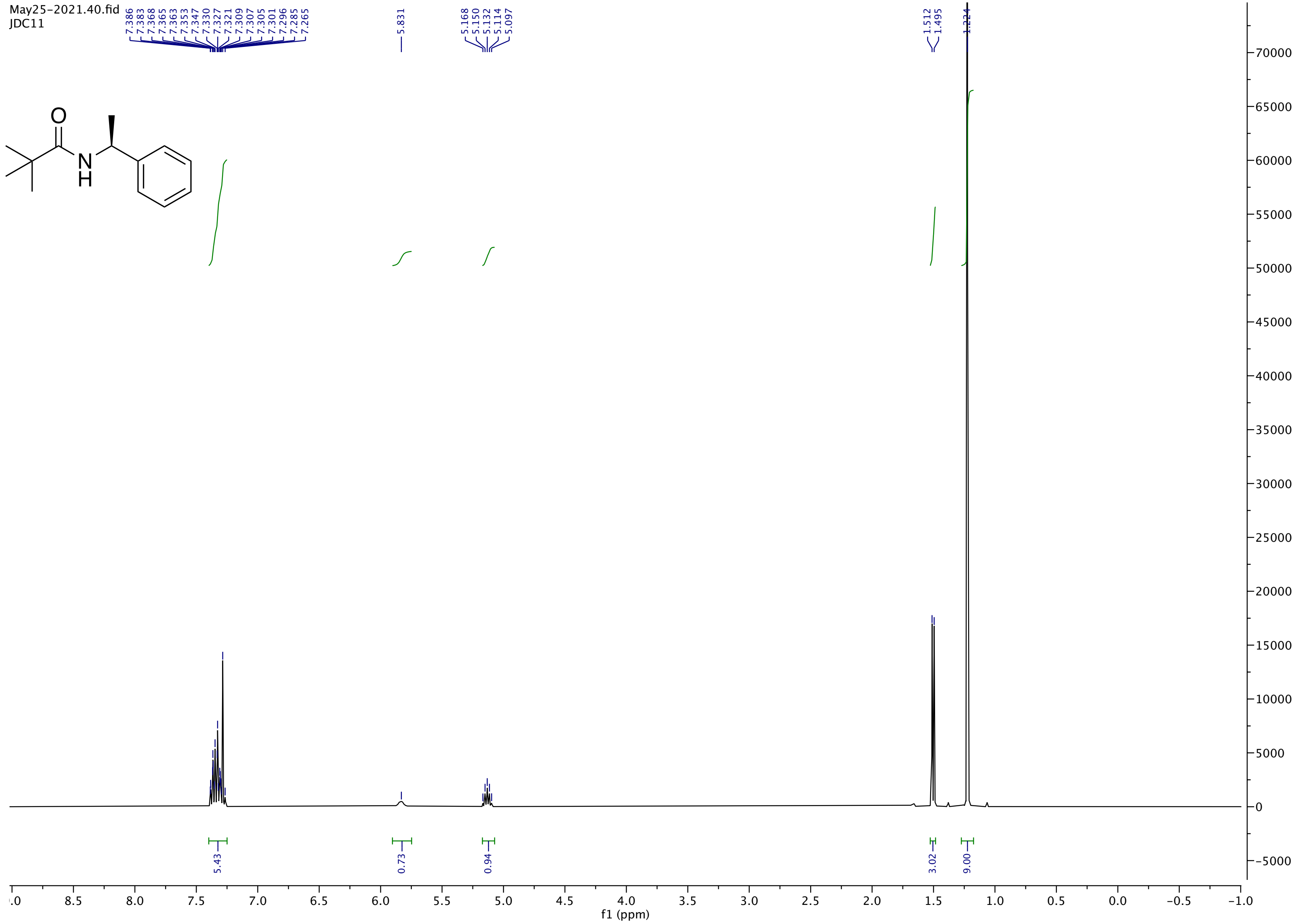

^{13}C NMR spectrum of (*S*)-*N*-(1-phenylethyl)pivalamide (**15**) (101 MHz, $CDCl_3$)

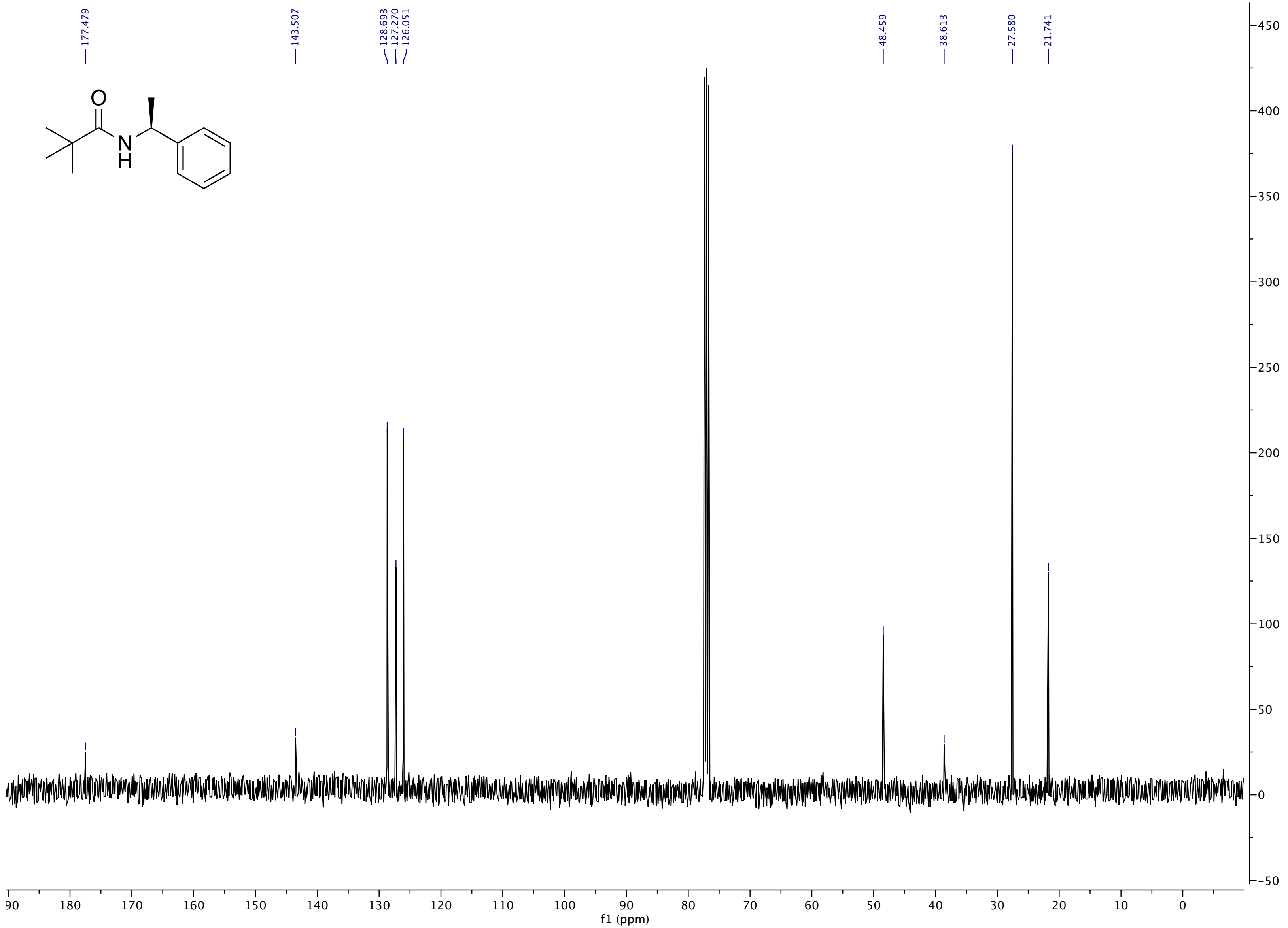

^{1}H NMR spectrum of *N*-hexyl-4-iodobenzamide (**16**) (400 MHz, $CDCl_3$)

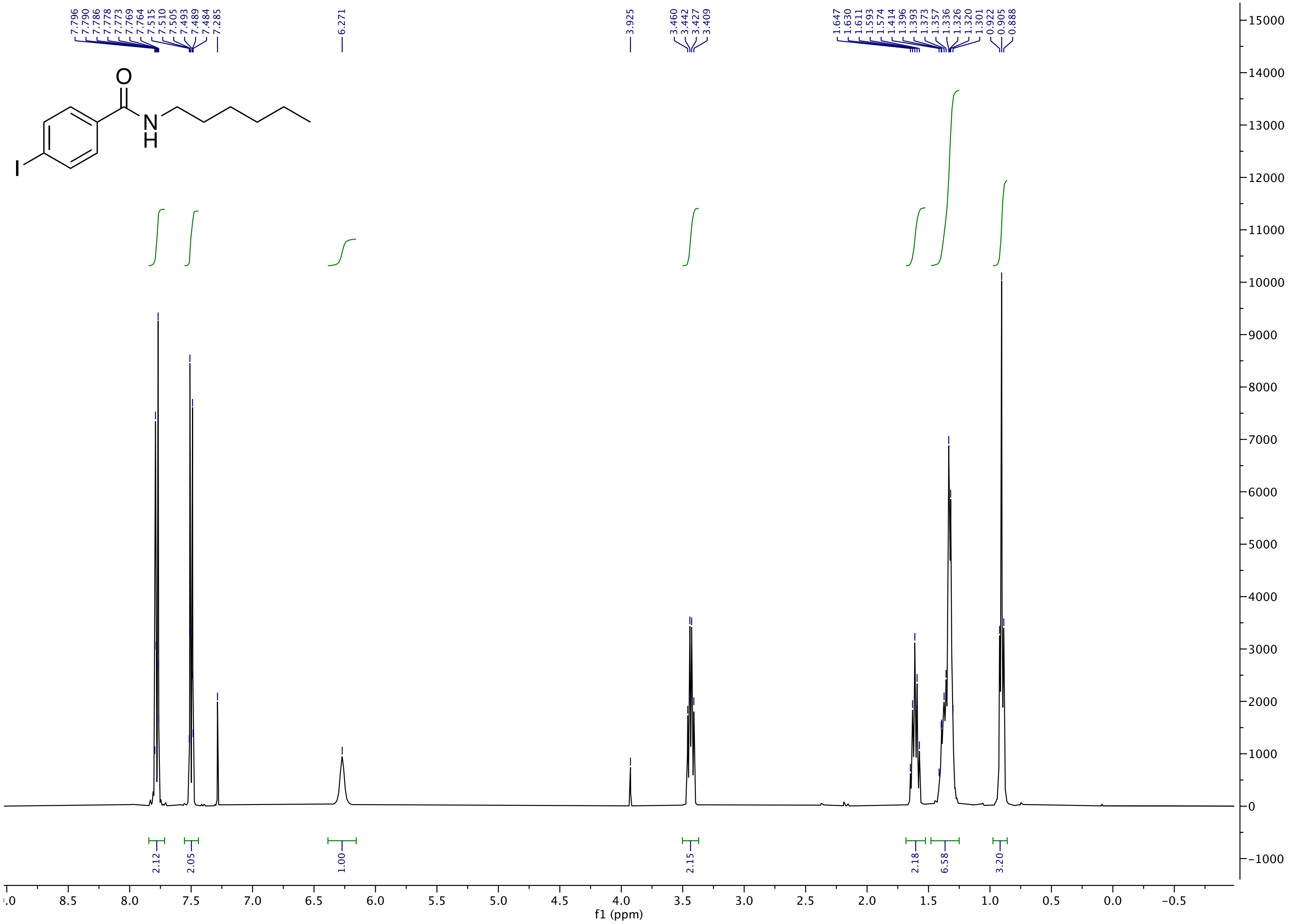

^{13}C NMR spectrum of *N*-hexyl-4-iodobenzamide (**16**) (101 MHz, $CDCl_3$)

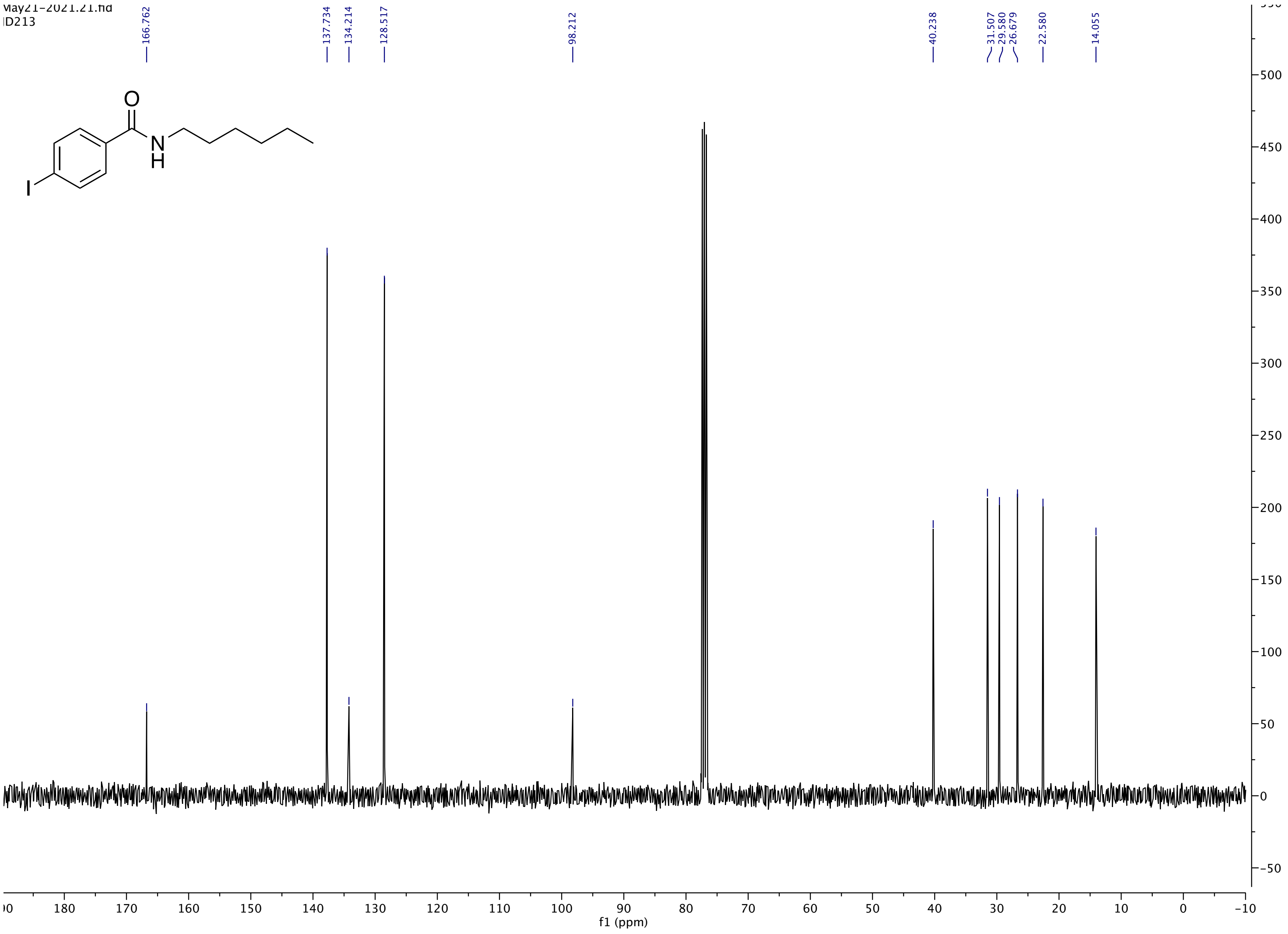

^{1}H NMR spectrum of 2-chloro-*N*-(4-methylbenzyl)benzamide (**17**) (400 MHz, $CDCl_3$)

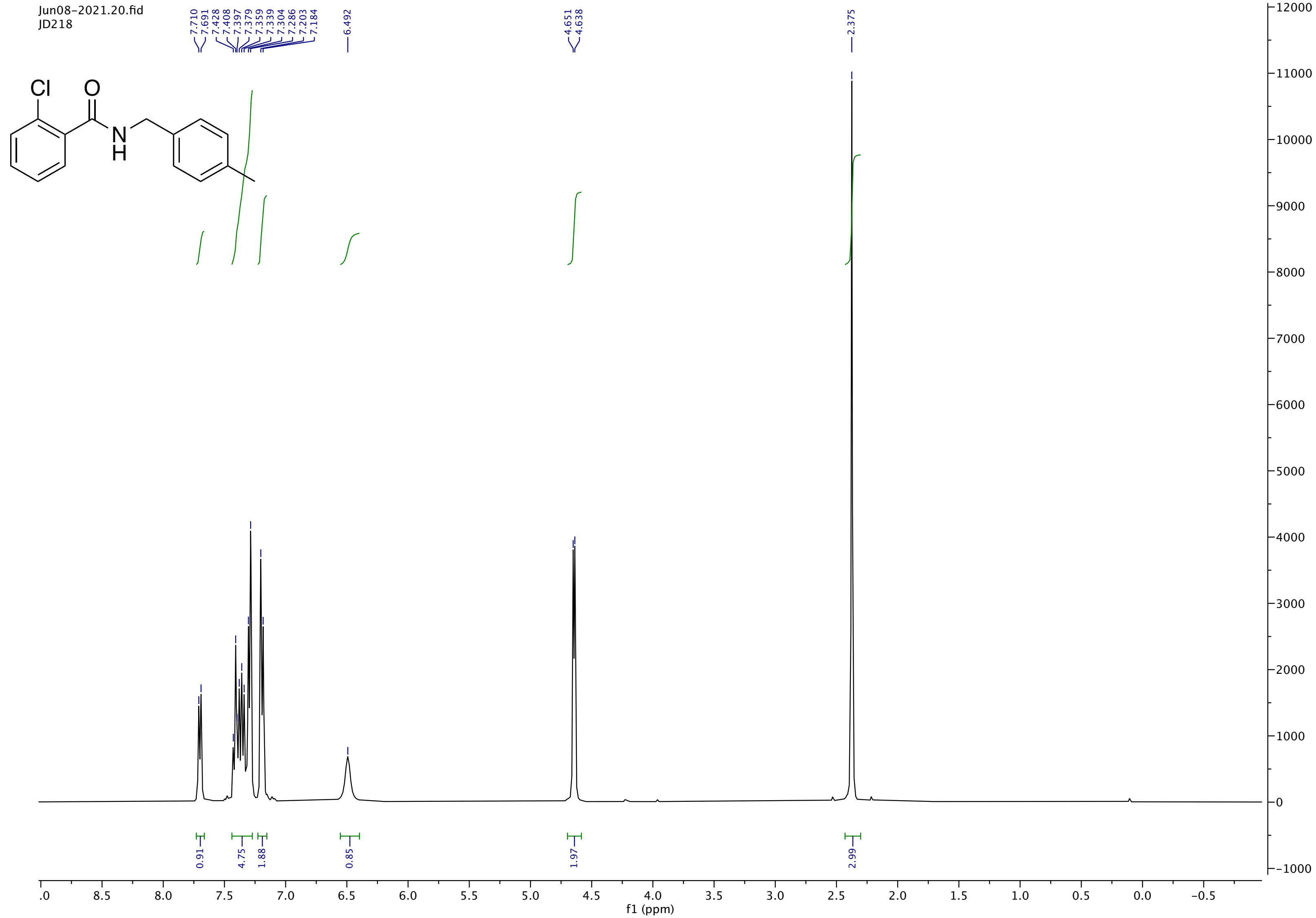

^{13}C NMR spectrum 2-chloro-*N*-(4-methylbenzyl)benzamide (**17**) (101 MHz, $CDCl_3$)

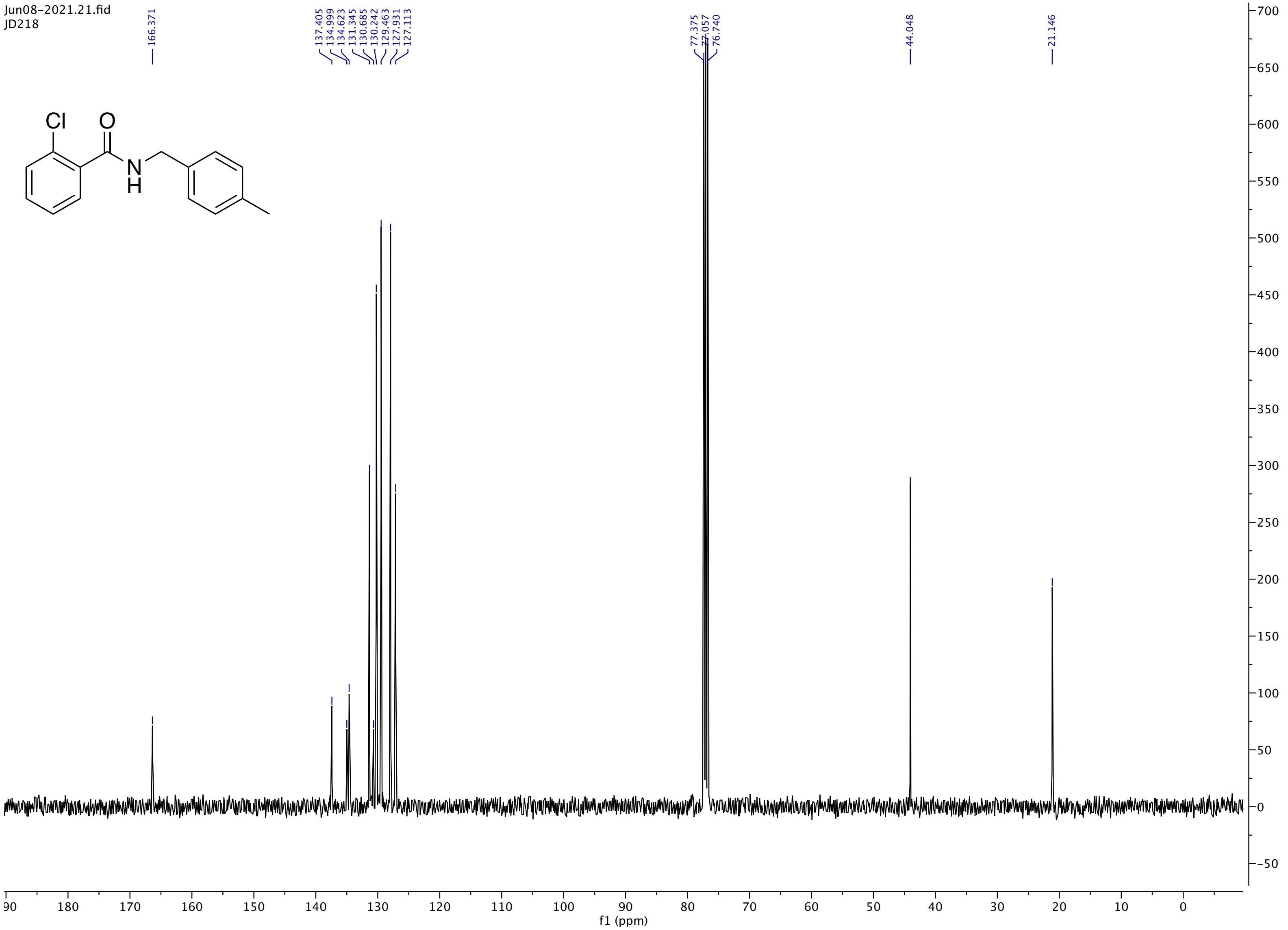

^{1}H NMR spectrum *N*-(4-methylbenzyl)hex-5-ynamide (**18**) (400 MHz, $CDCl_3$)

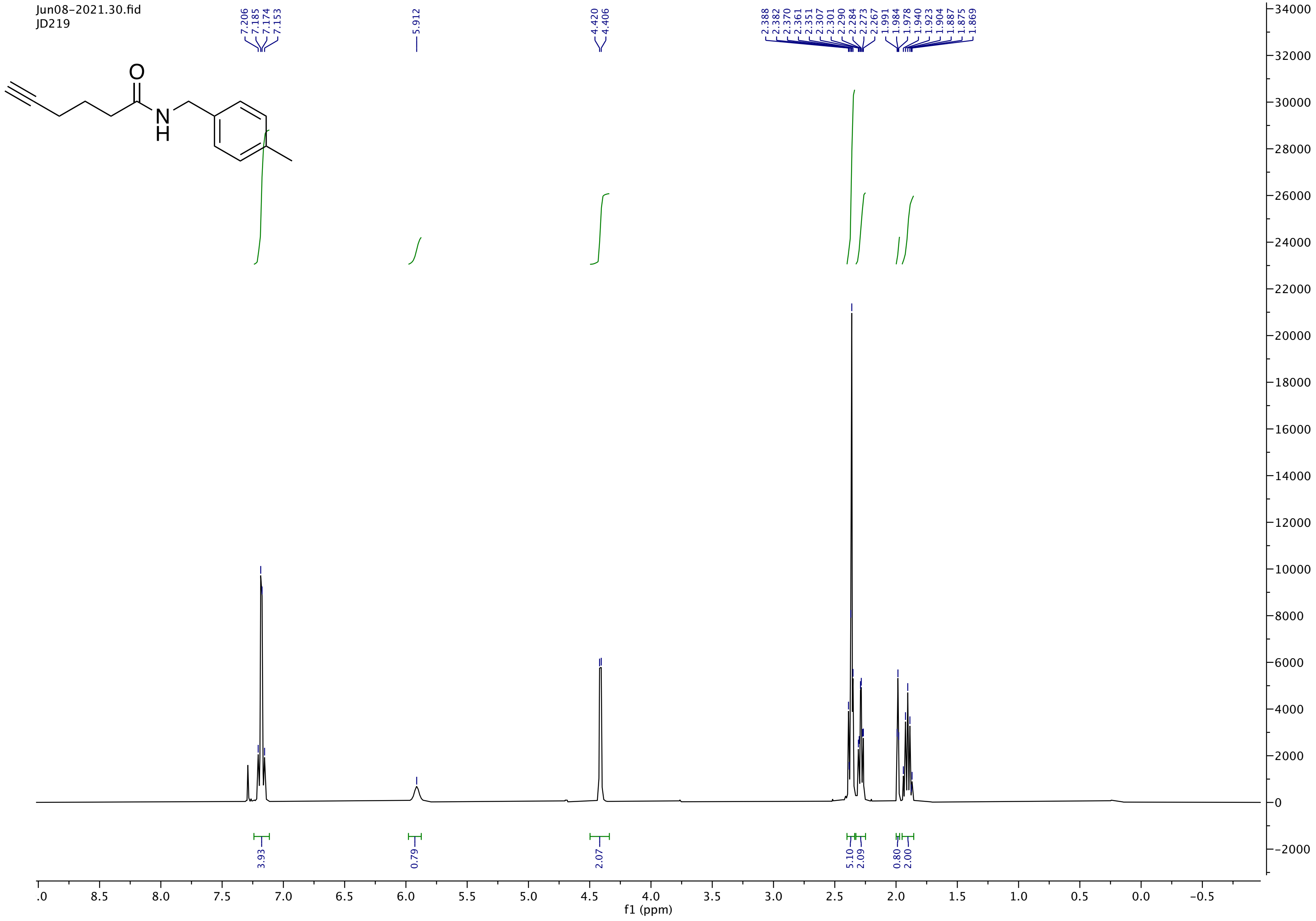

^{13}C NMR spectrum *N*-(4-methylbenzyl)hex-5-ynamide (**18**) (101 MHz, $CDCl_3$)

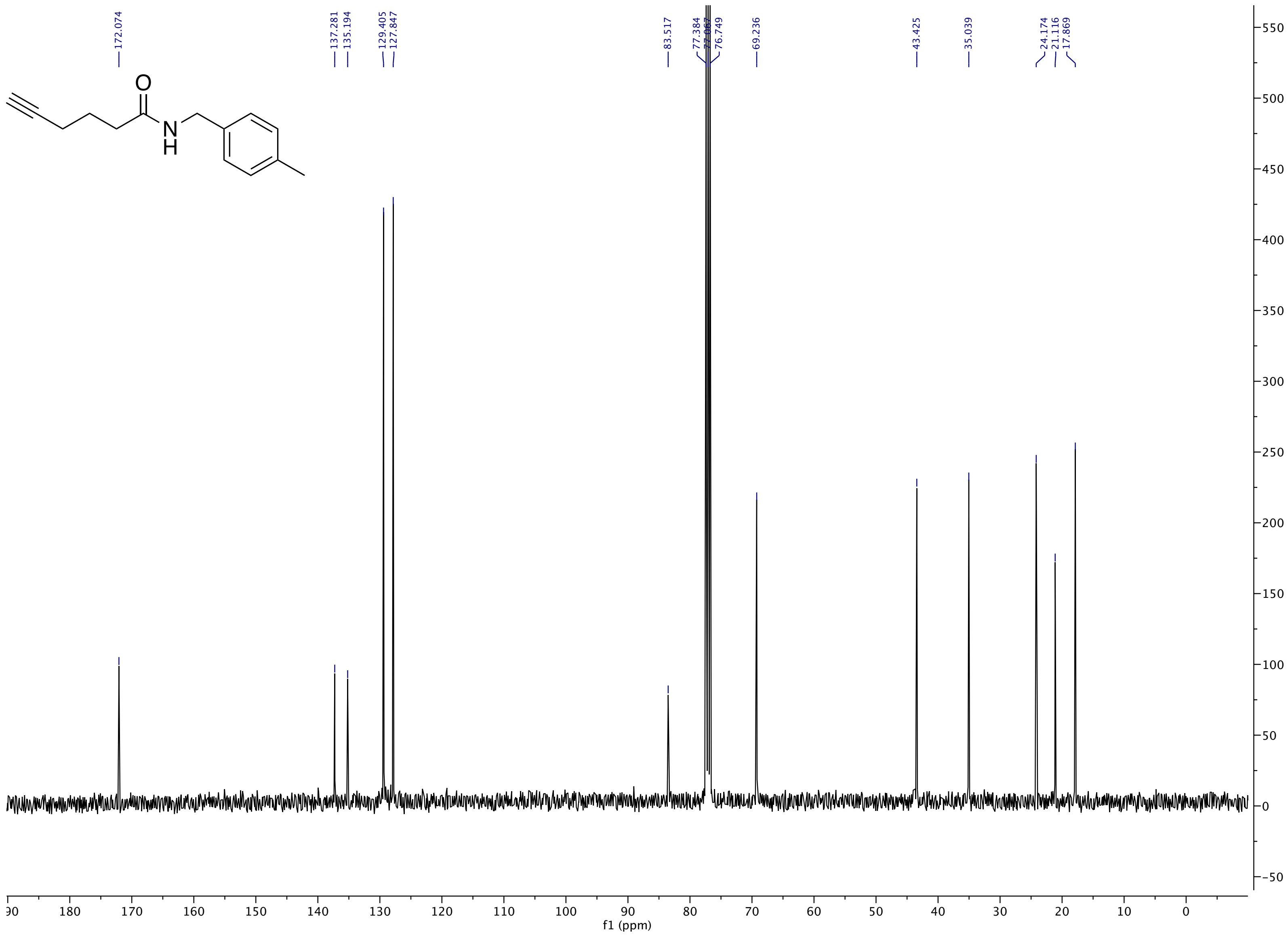

^{1}H NMR spectrum *N*-(4-methylbenzyl)cinnamamide (**19**) (400 MHz, $CDCl_3$)

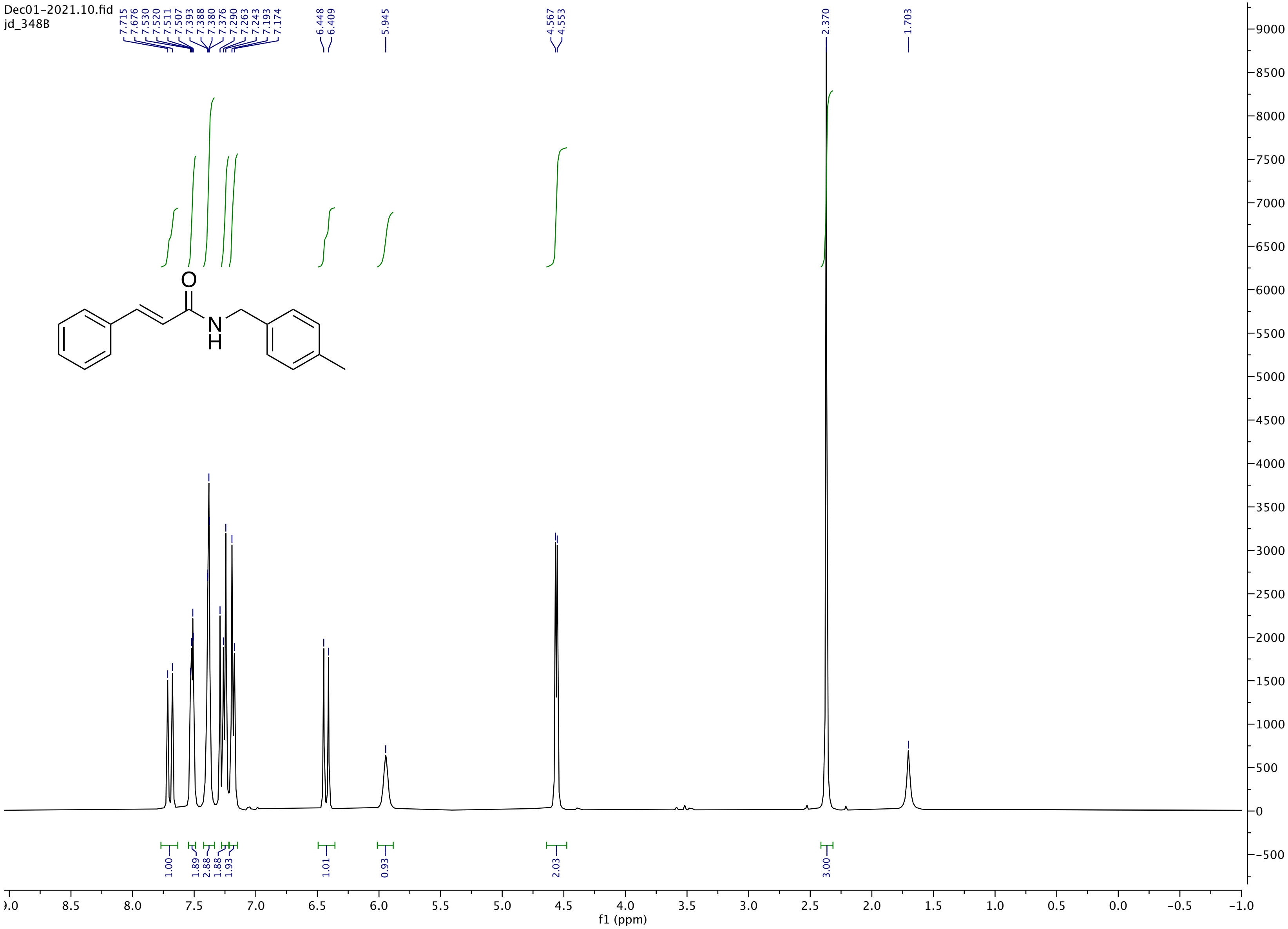

^{13}C NMR spectrum *N*-(4-methylbenzyl)cinnamamide (**19**) (101 MHz, $CDCl_3$)

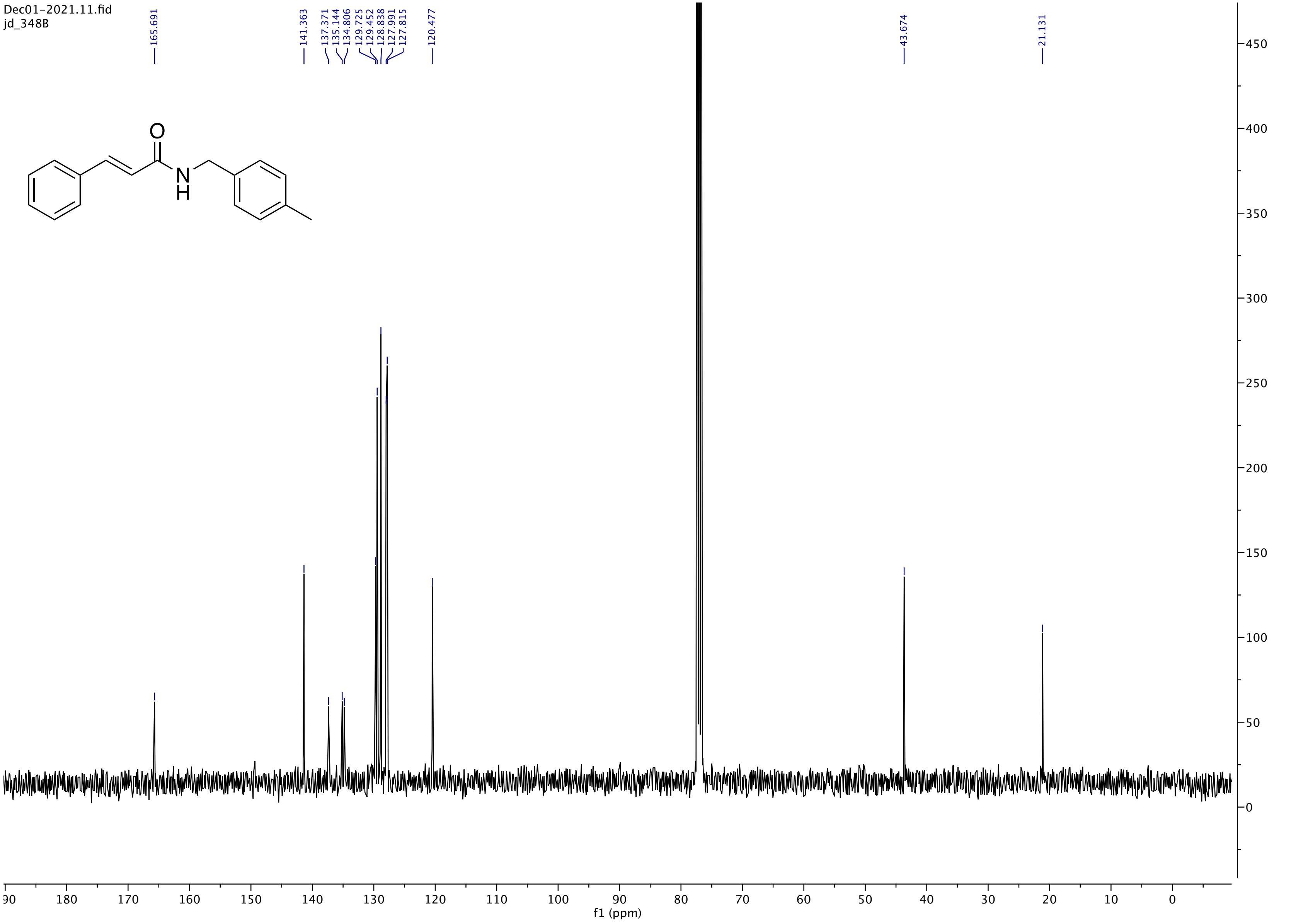

^{1}H NMR spectrum *N*-octyloctanamide (**20**) (400 MHz, $CDCl_3$)

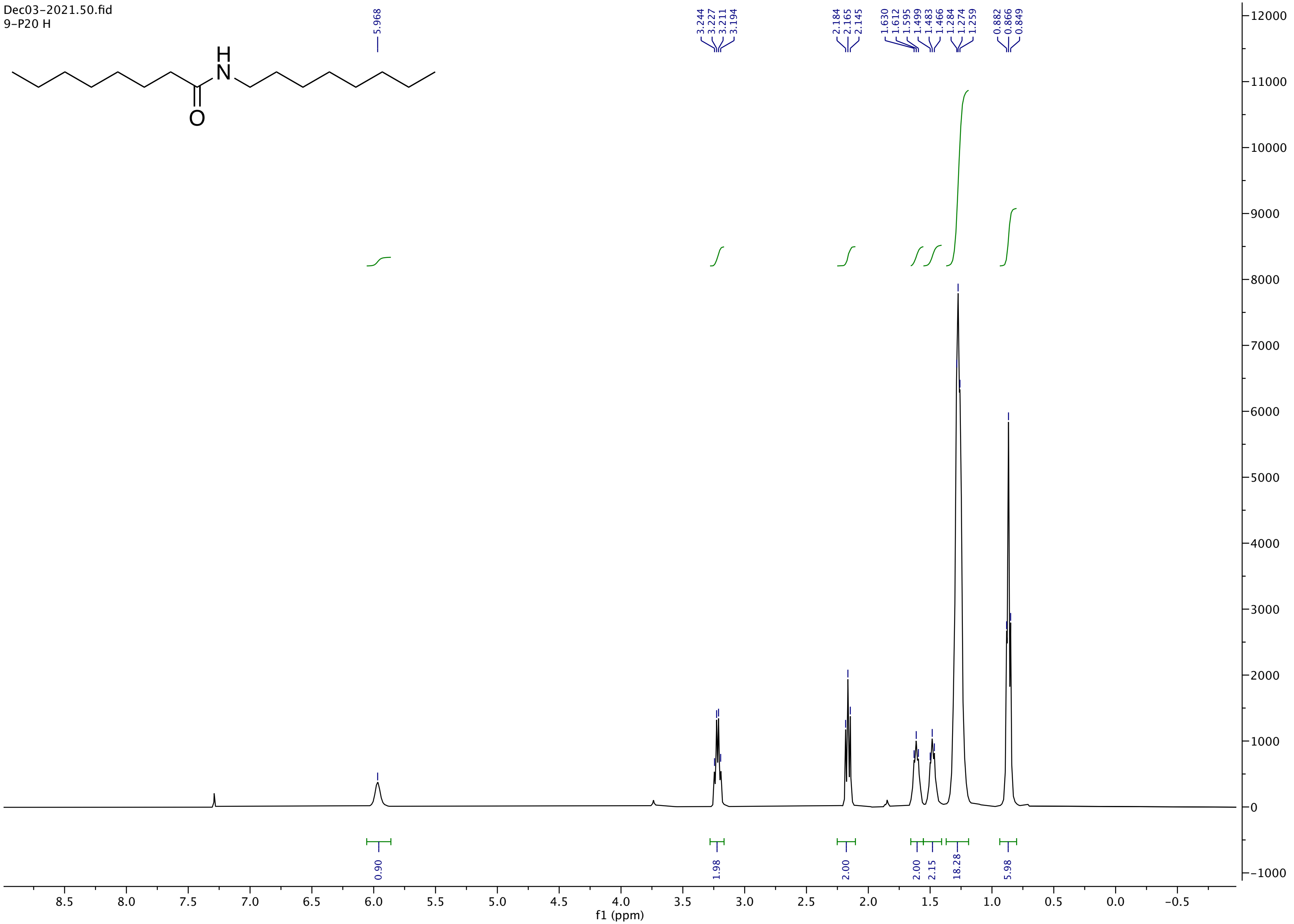

^{13}C NMR spectrum *N*-octyloctanamide (**20**) (101 MHz, $CDCl_3$)

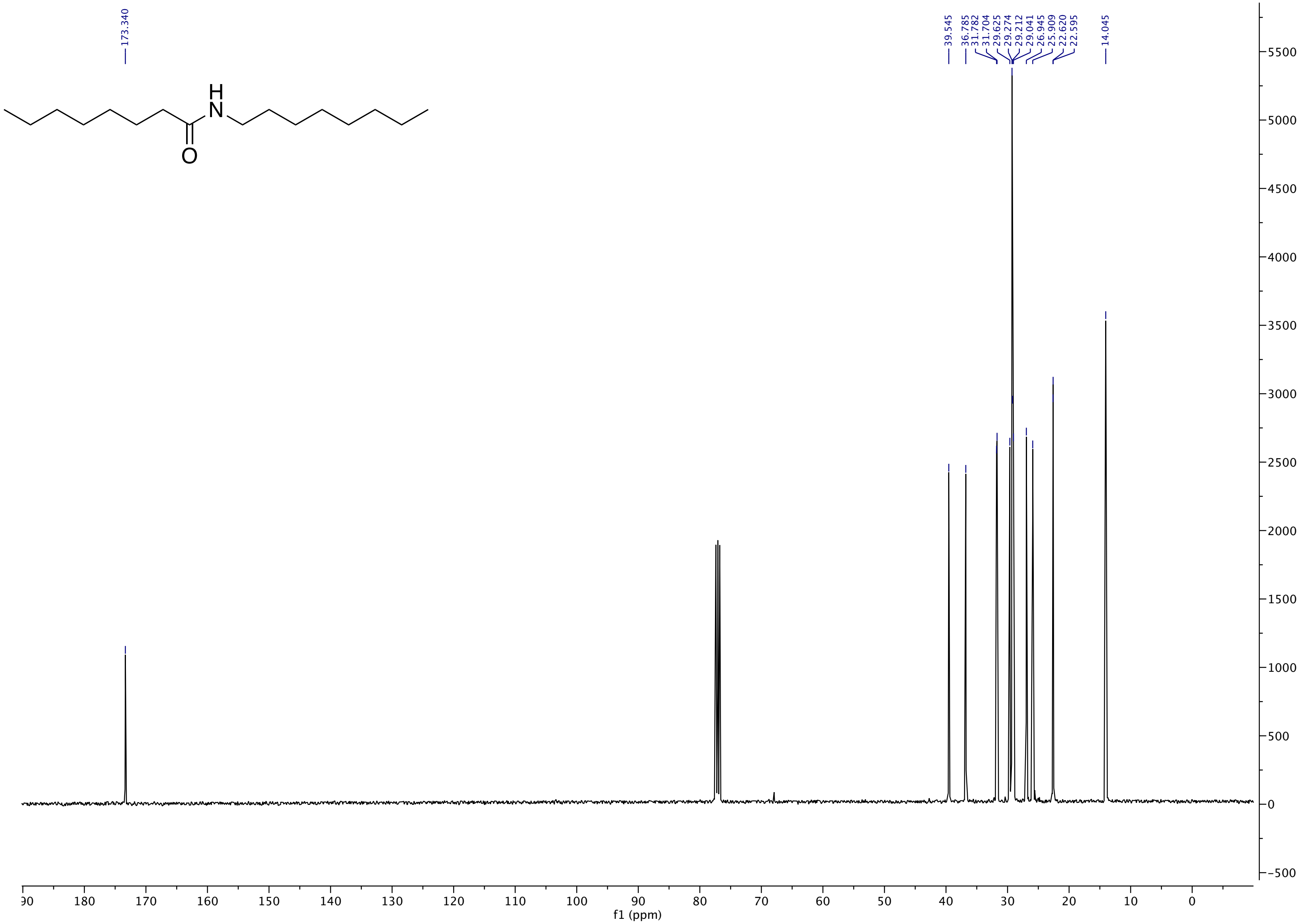

^{1}H NMR spectrum *N*-(4-methylbenzyl)nicotinamide (**21**) (400 MHz, $CDCl_3$)

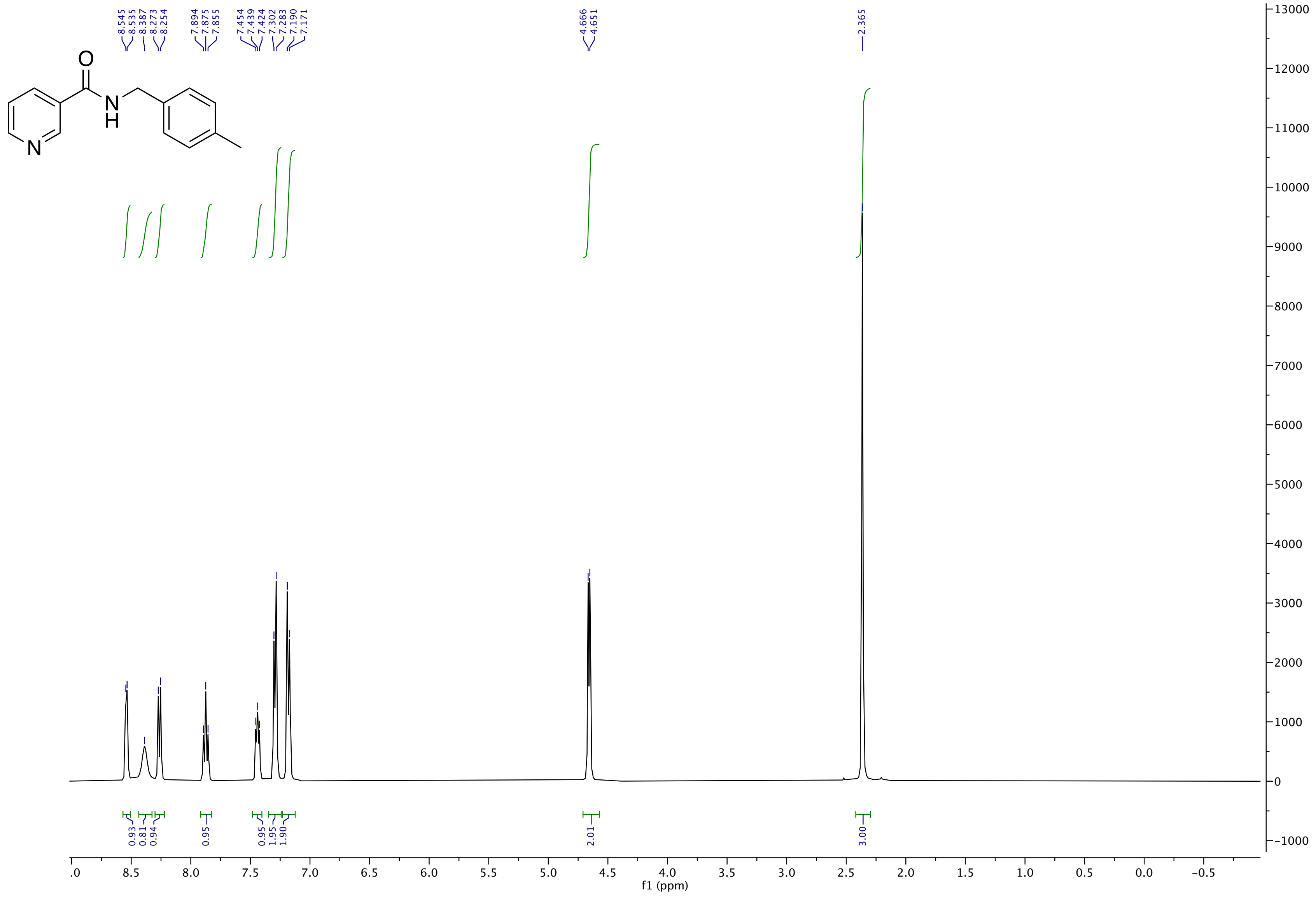

^{13}C NMR spectrum *N*-(4-methylbenzyl)nicotinamide (**21**) (101 MHz, $CDCl_3$)

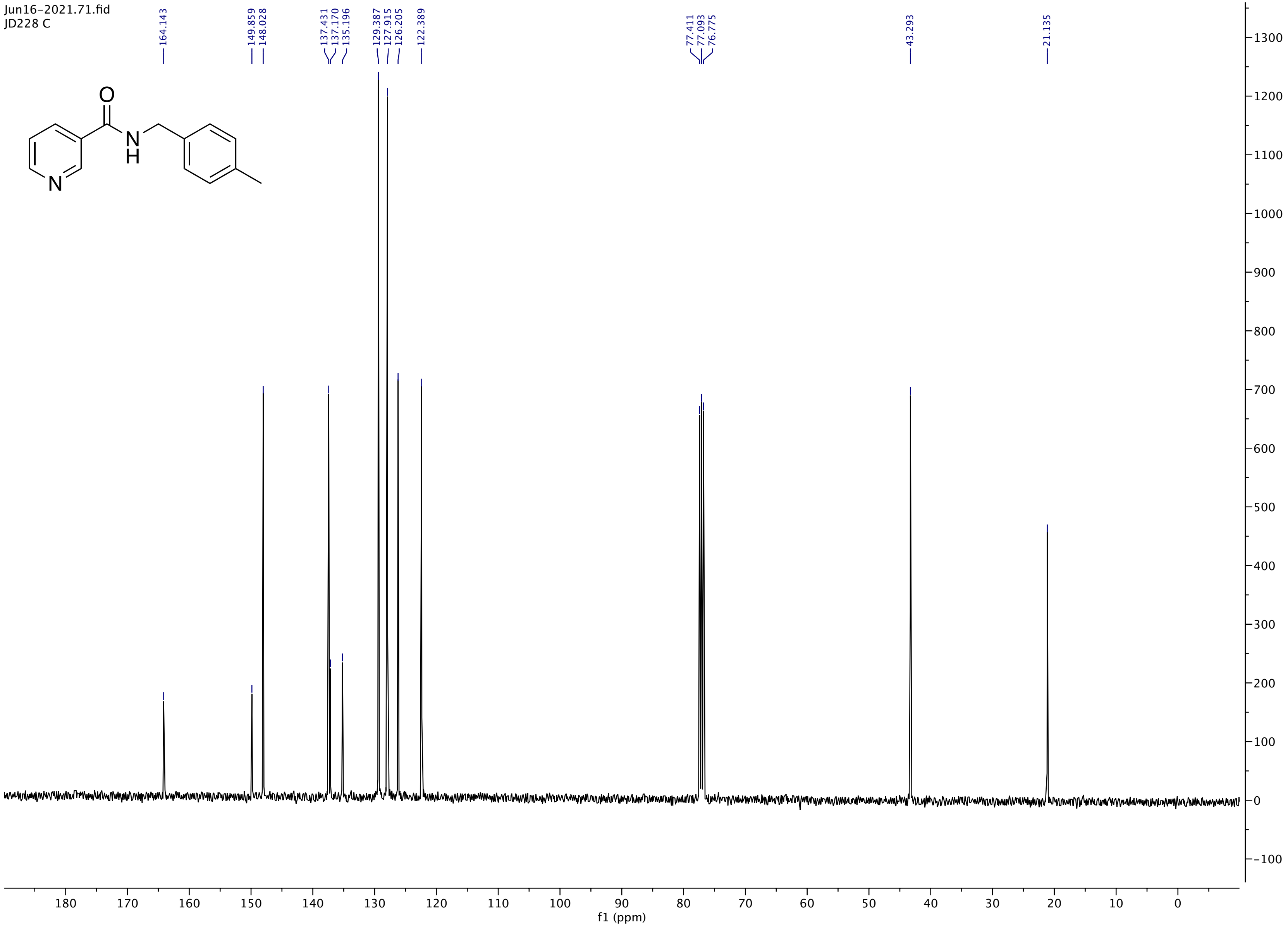

^{1}H NMR Spectrum of Benzyl (*S*)-(1-(benzylamino)-1-oxopropan-2-yl)carbamate (**22**) (400 MHz, $CDCl_3$)

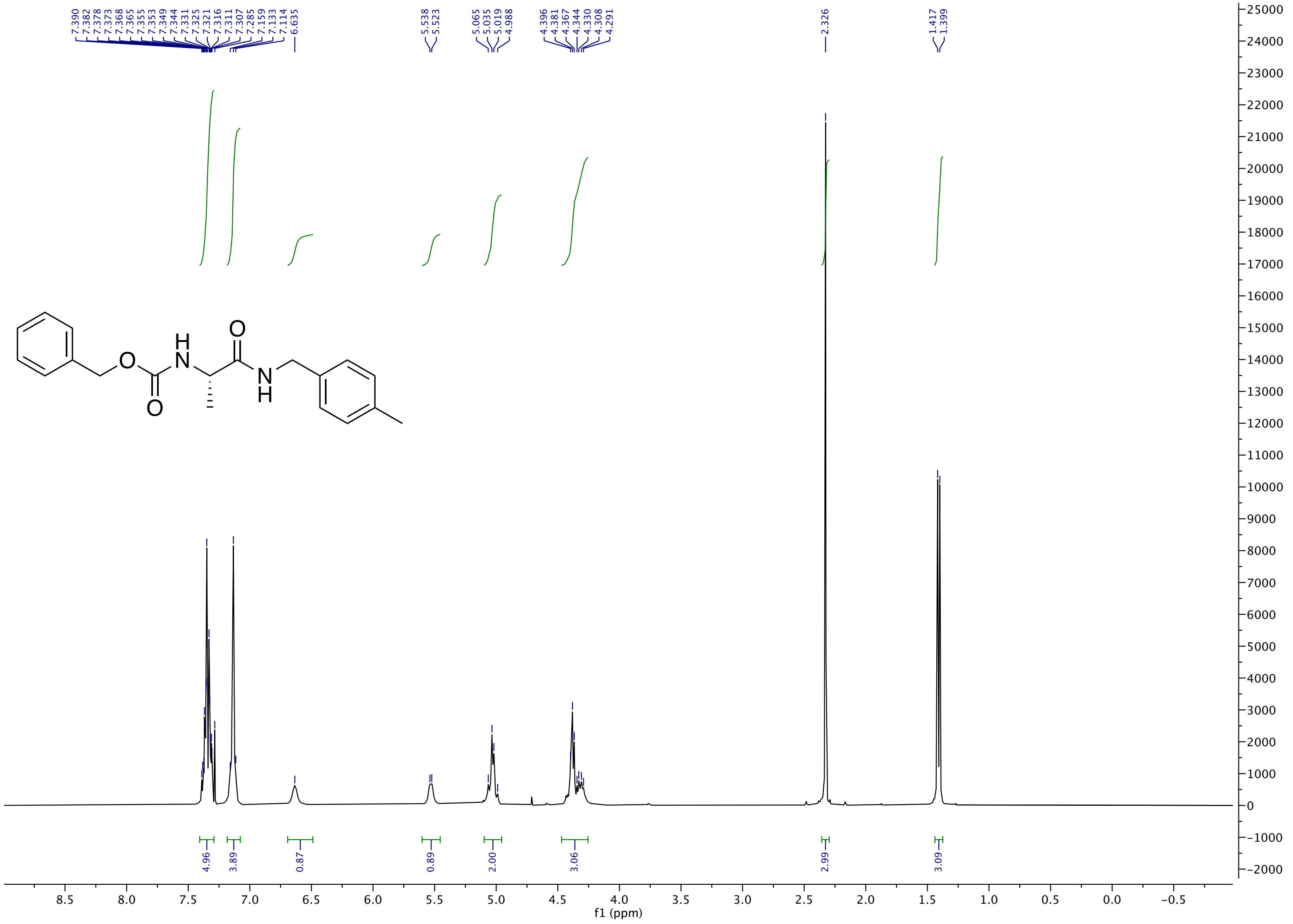

^{13}C NMR Spectrum of Benzyl (*S*)-(1-(benzylamino)-1-oxopropan-2-yl)carbamate (**22**) (101 MHz, $CDCl_3$)

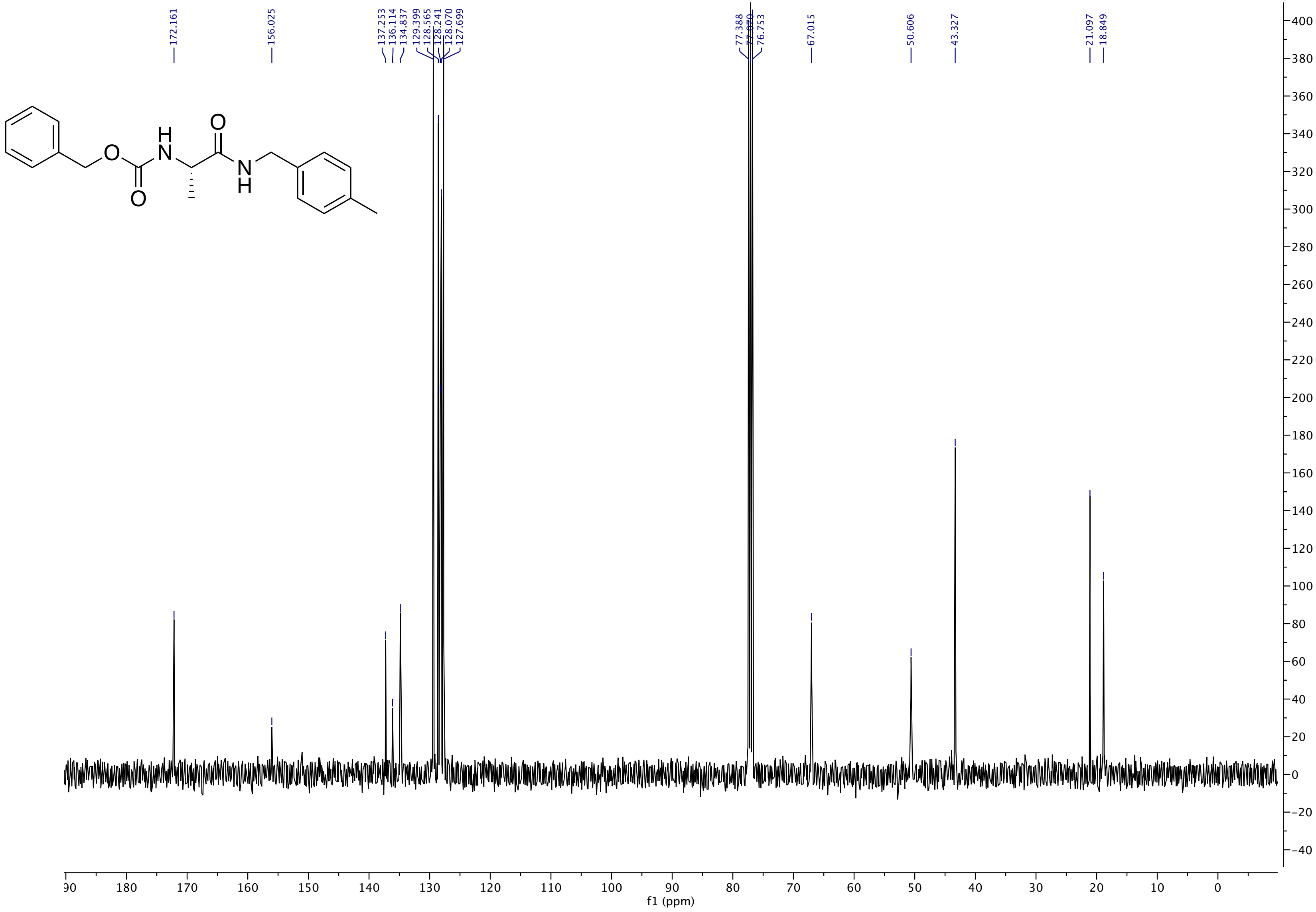

^{1}H NMR of spectrum of *tert*-butyl (*S*)-(1-(benzylamino)-1-oxopropan-2-yl)carbamate (**23**) (400 MHz, $CDCl_3$)

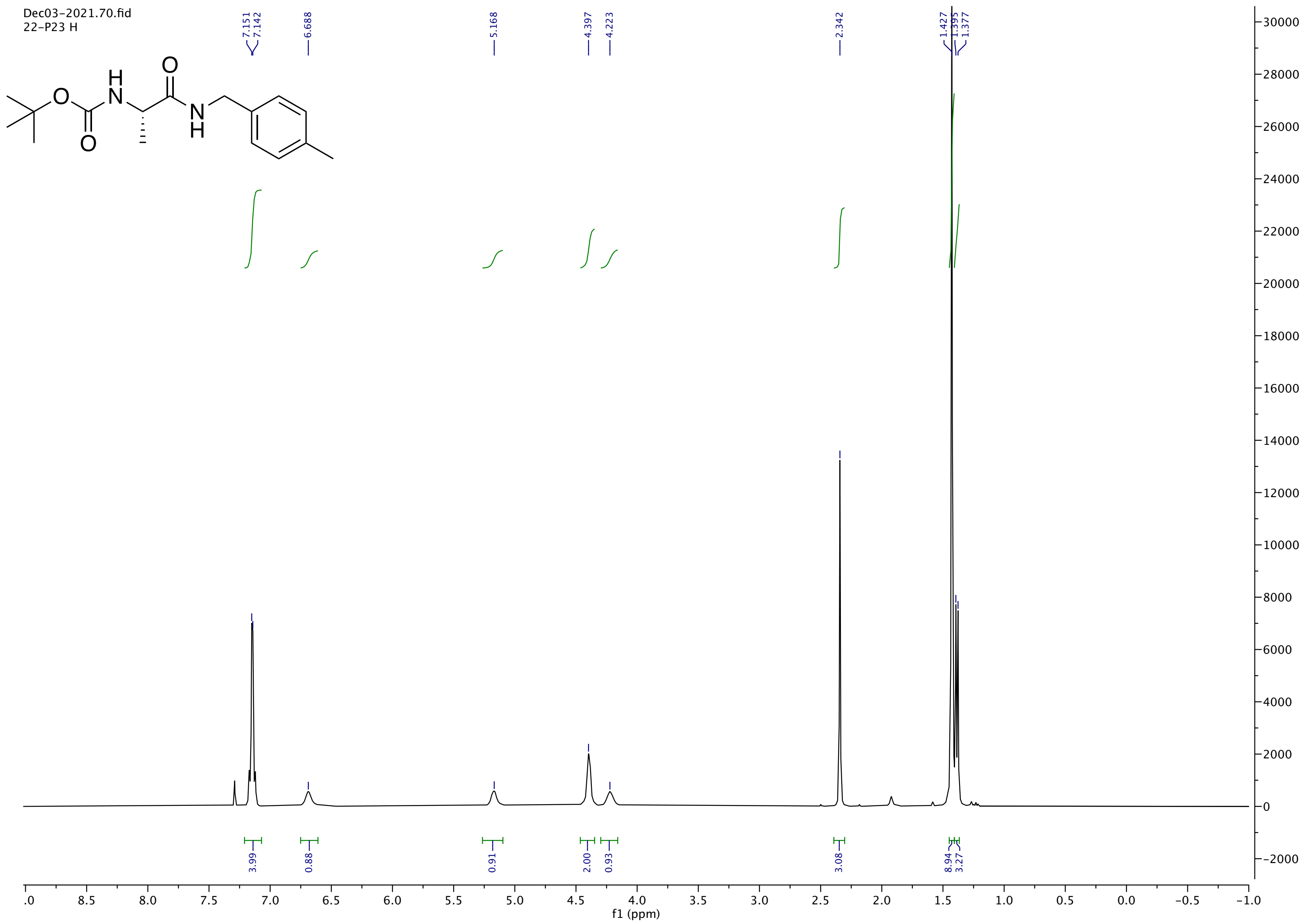

^{13}C NMR of spectrum of *tert*-butyl (*S*)-(1-(benzylamino)-1-oxopropan-2-yl)carbamate (**23**) (101 MHz, $CDCl_3$)

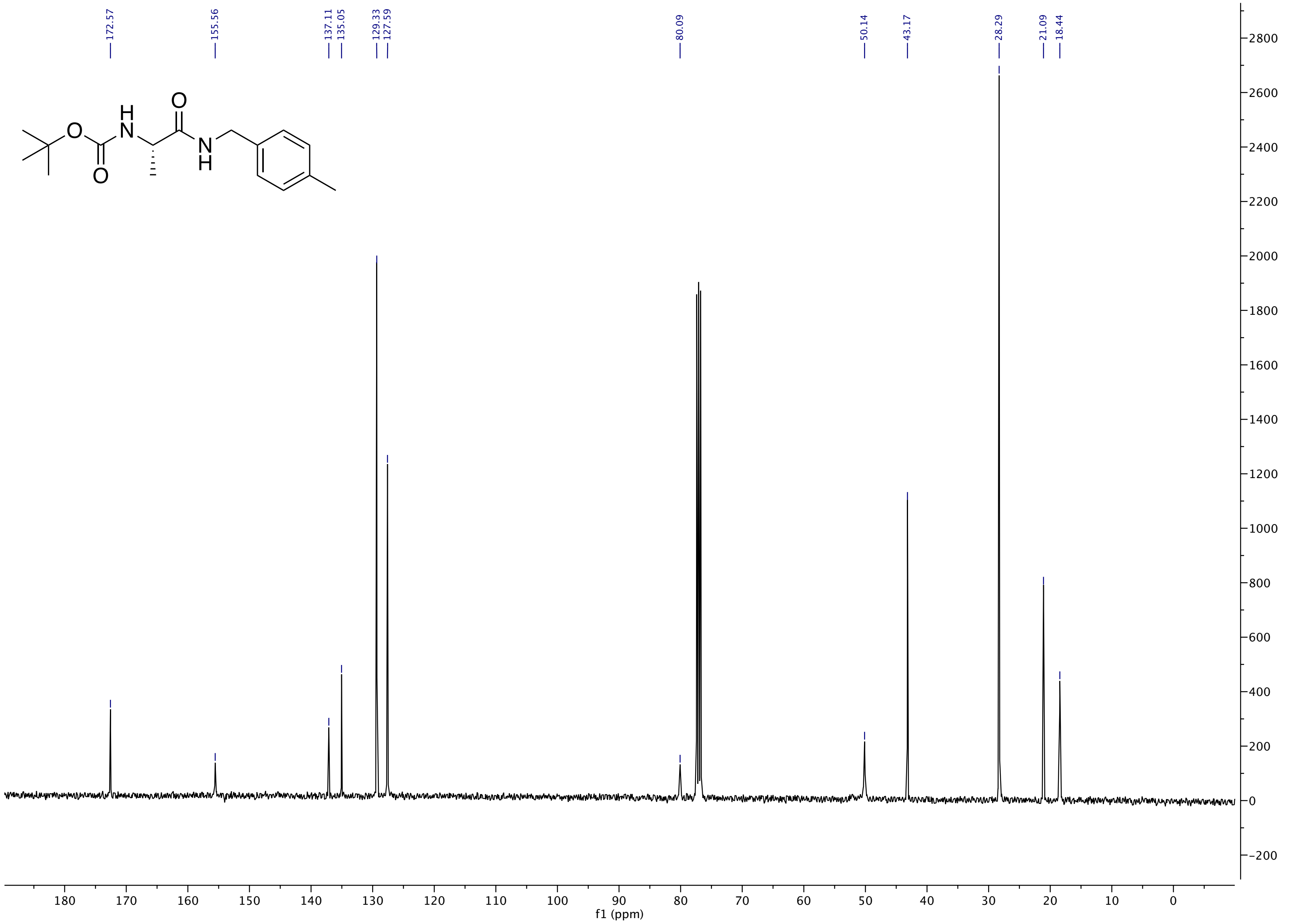

^{1}H NMR spectrum of N-(4-methylbenzyl)ferrocenecarboxamide (**24**) (400 MHz, $CDCl_3$)

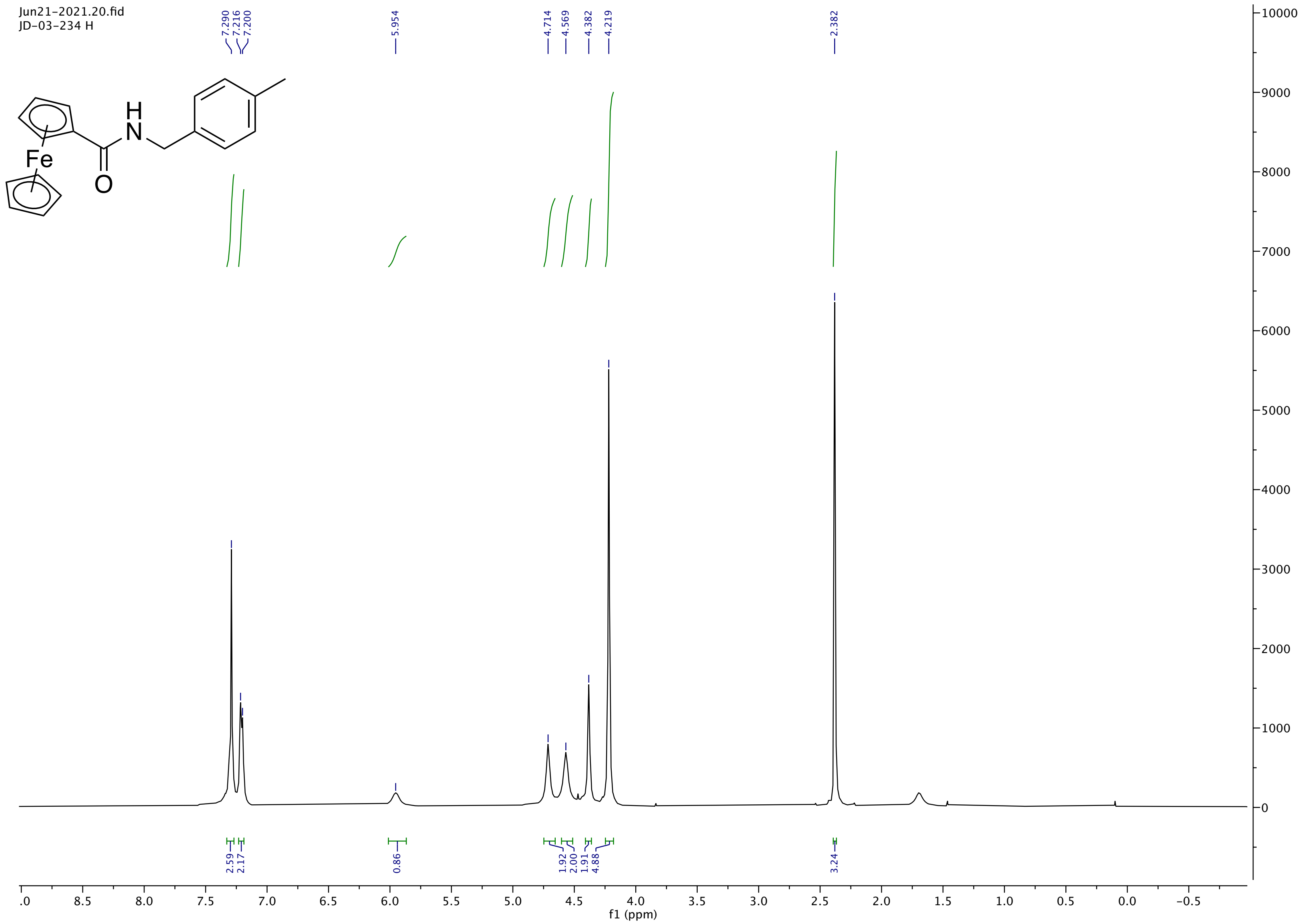

^{13}C NMR spectrum of N-(4-methylbenzyl)ferrocenecarboxamide (**24**) (101 MHz, $CDCl_3$)

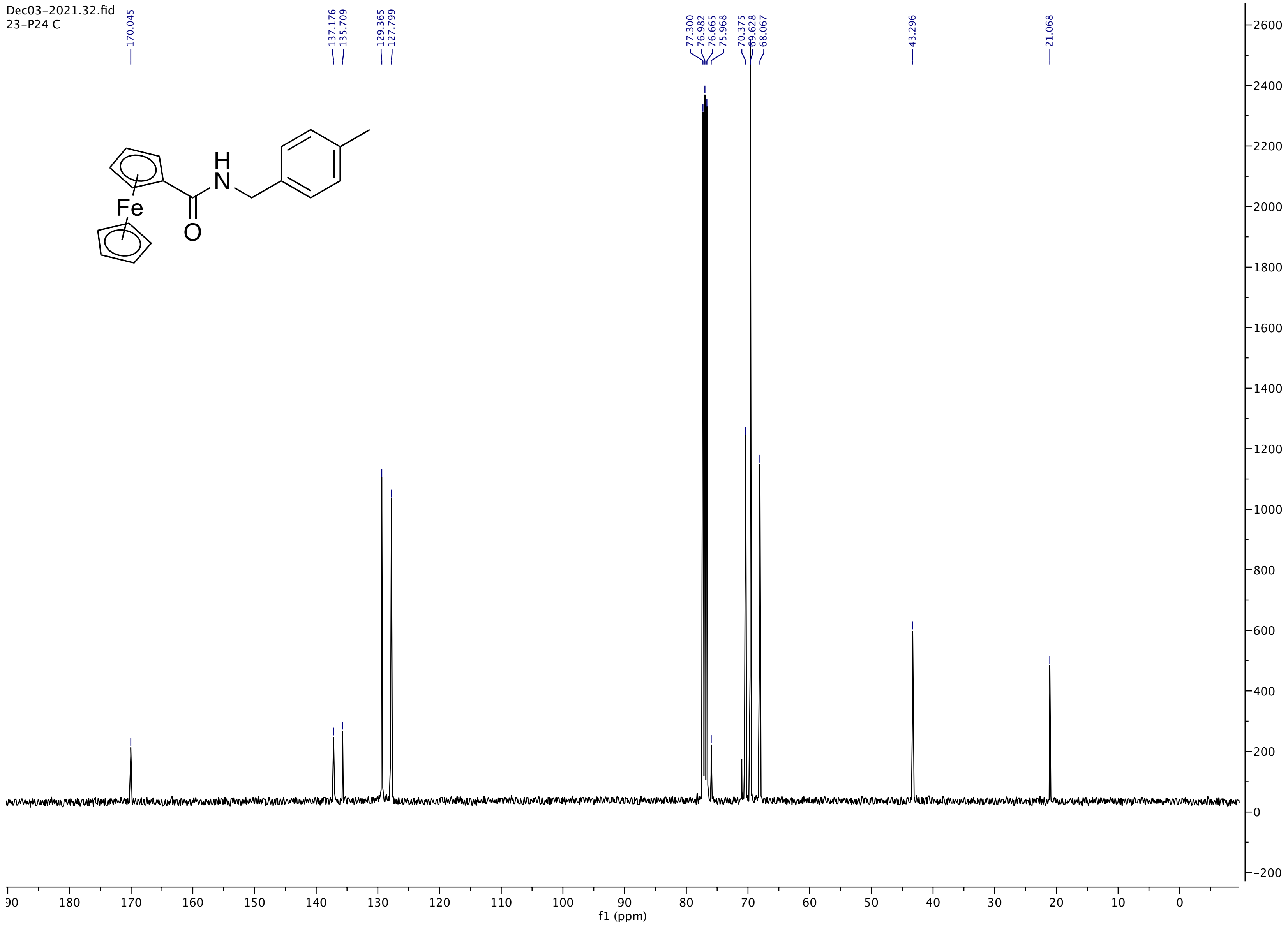

^{1}H NMR spectrum of 2-amino-*N*-(4-methylbenzyl)benzamide (**25**) (400 MHz, $CDCl_3$)

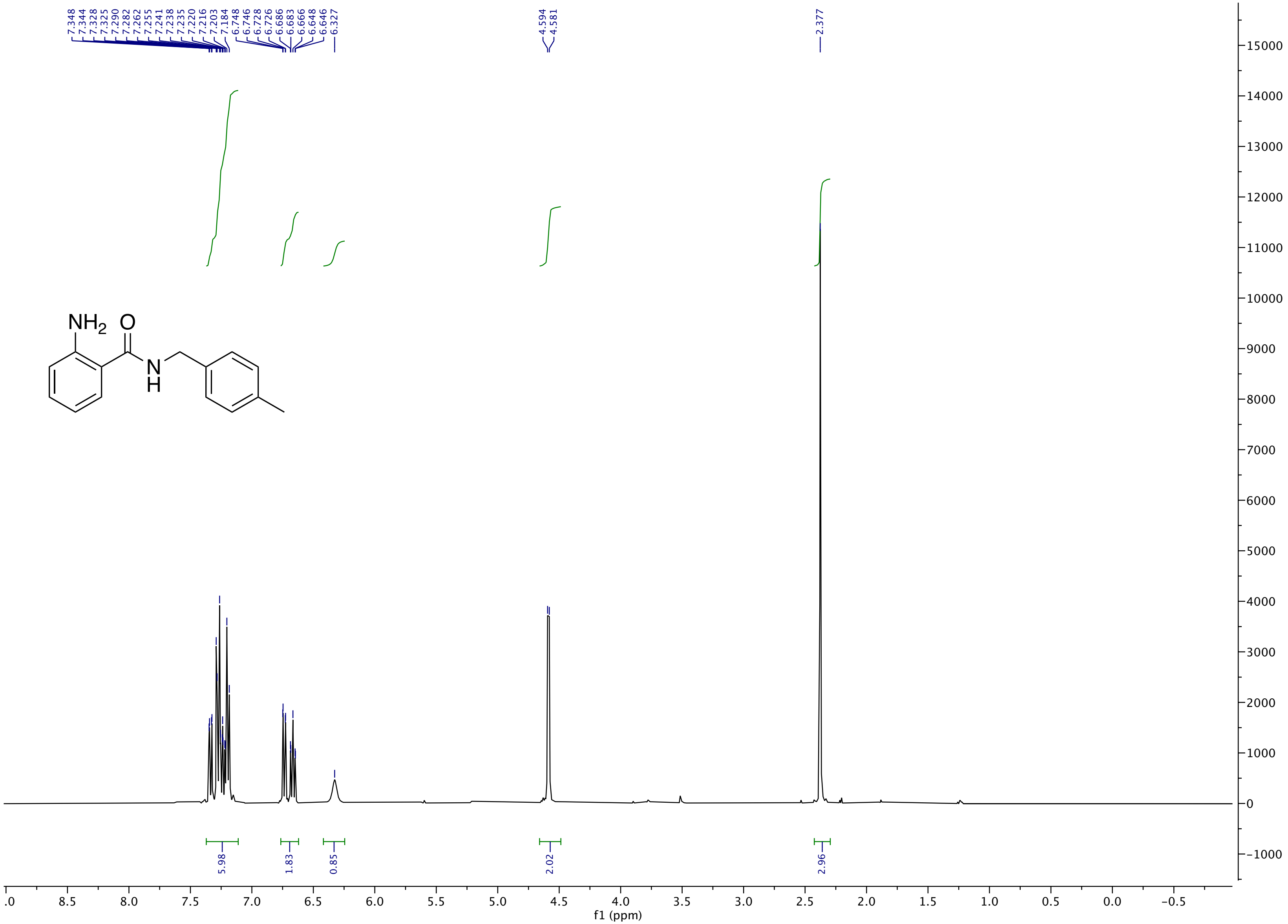

^{13}C NMR spectrum of 2-amino-*N*-(4-methylbenzyl)benzamide (**25**) (101 MHz, $CDCl_3$)

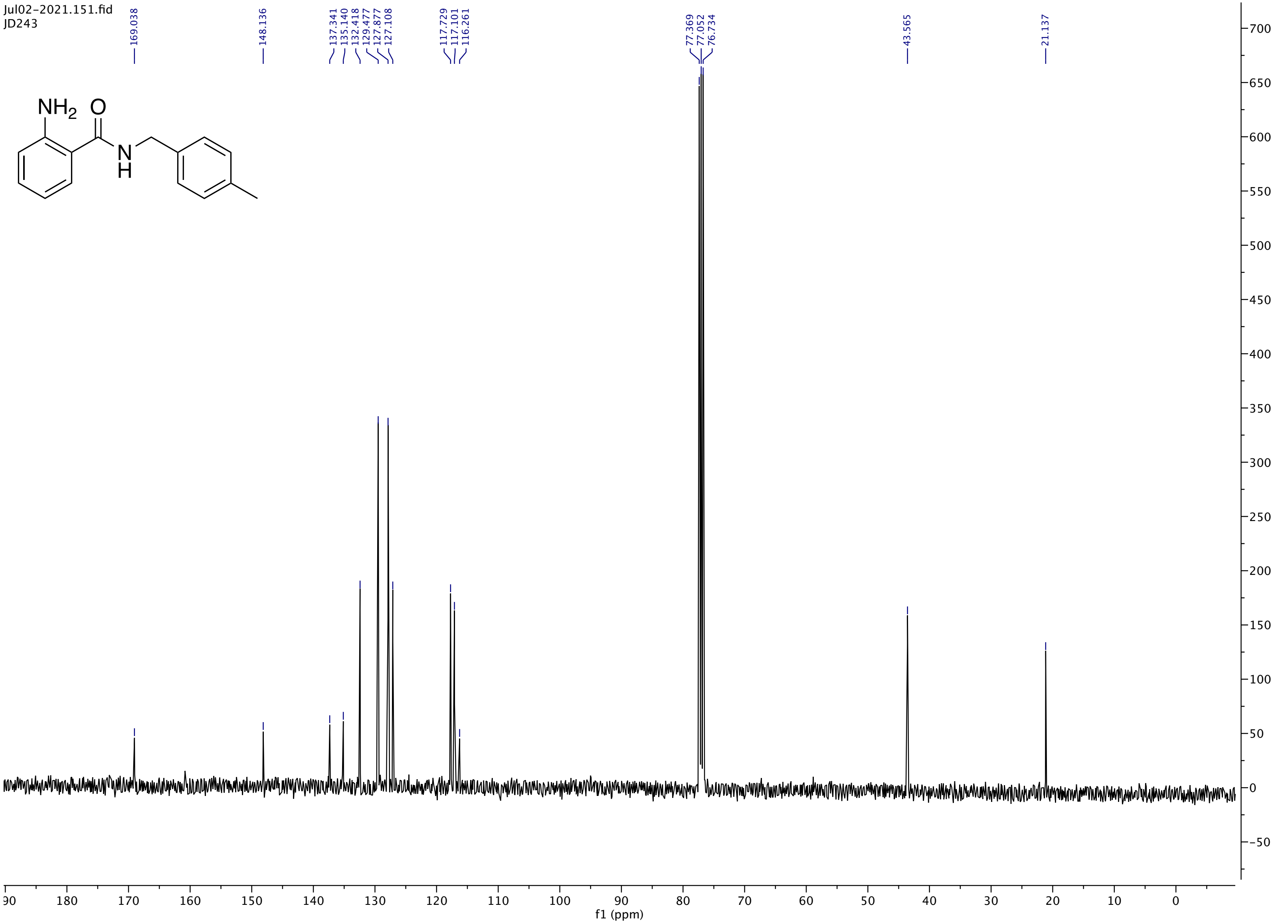

^{1}H NMR spectrum of 3-(4-chlorophenyl)-*N*-phenylpropanamide (**26**) (400 MHz, $CDCl_3$)

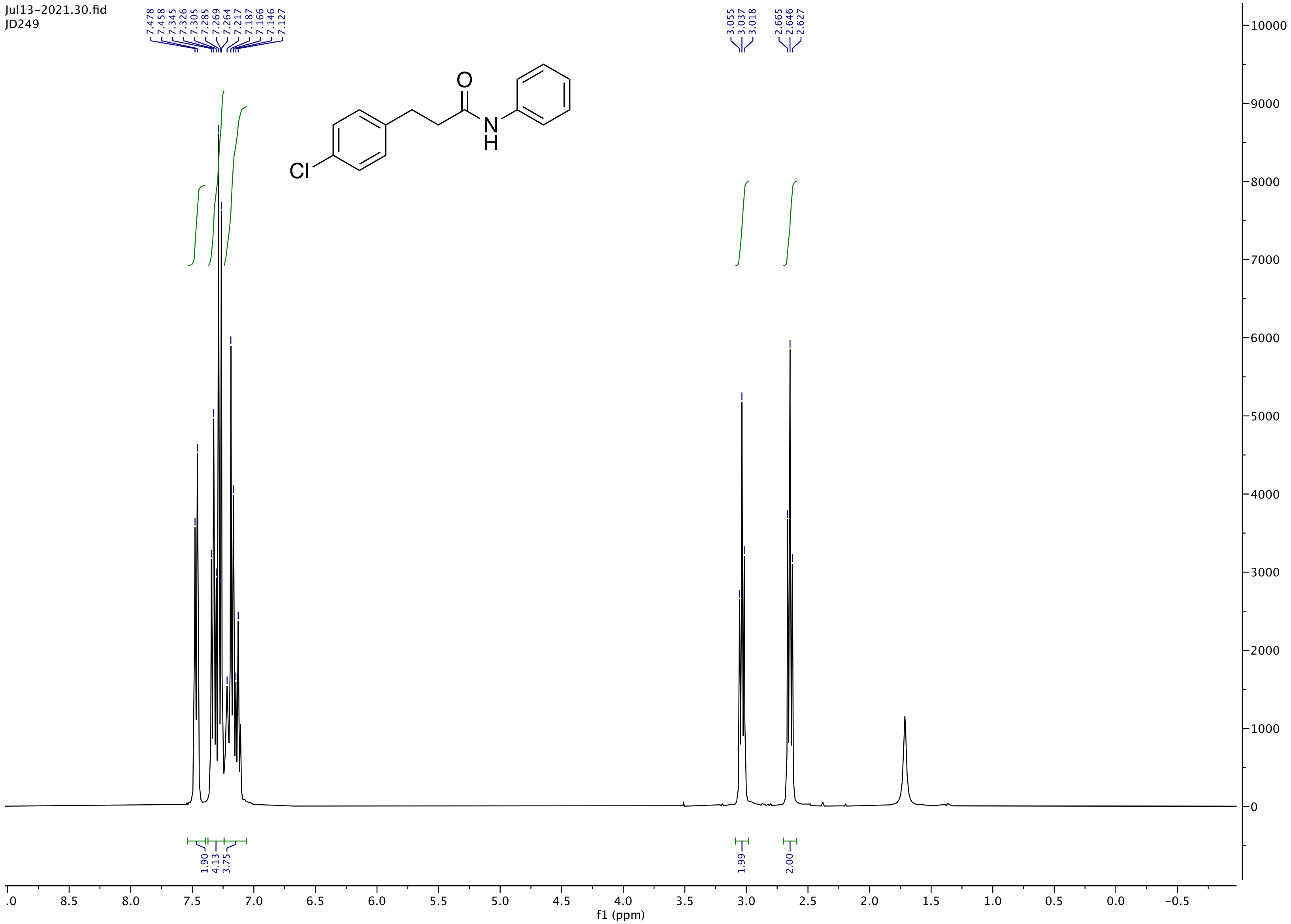

^{13}C NMR spectrum of 3-(4-chlorophenyl)-*N*-phenylpropanamide (**26**) (101 MHz, $CDCl_3$)

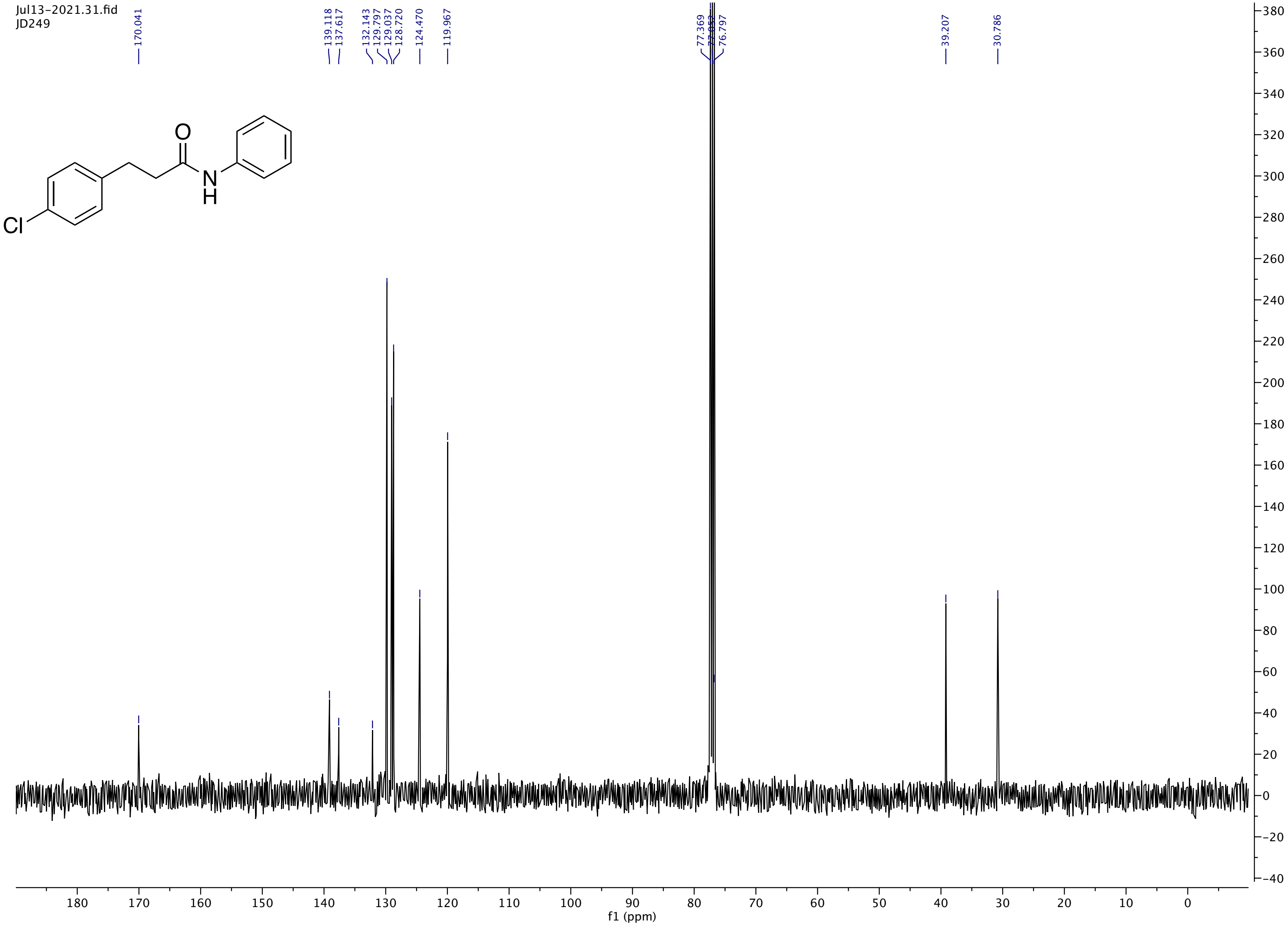

^{1}H NMR spectrum of N^1,N^3-bis(4-methylbenzyl)malonamide (**27**) (400 MHz, $CDCl_3$)

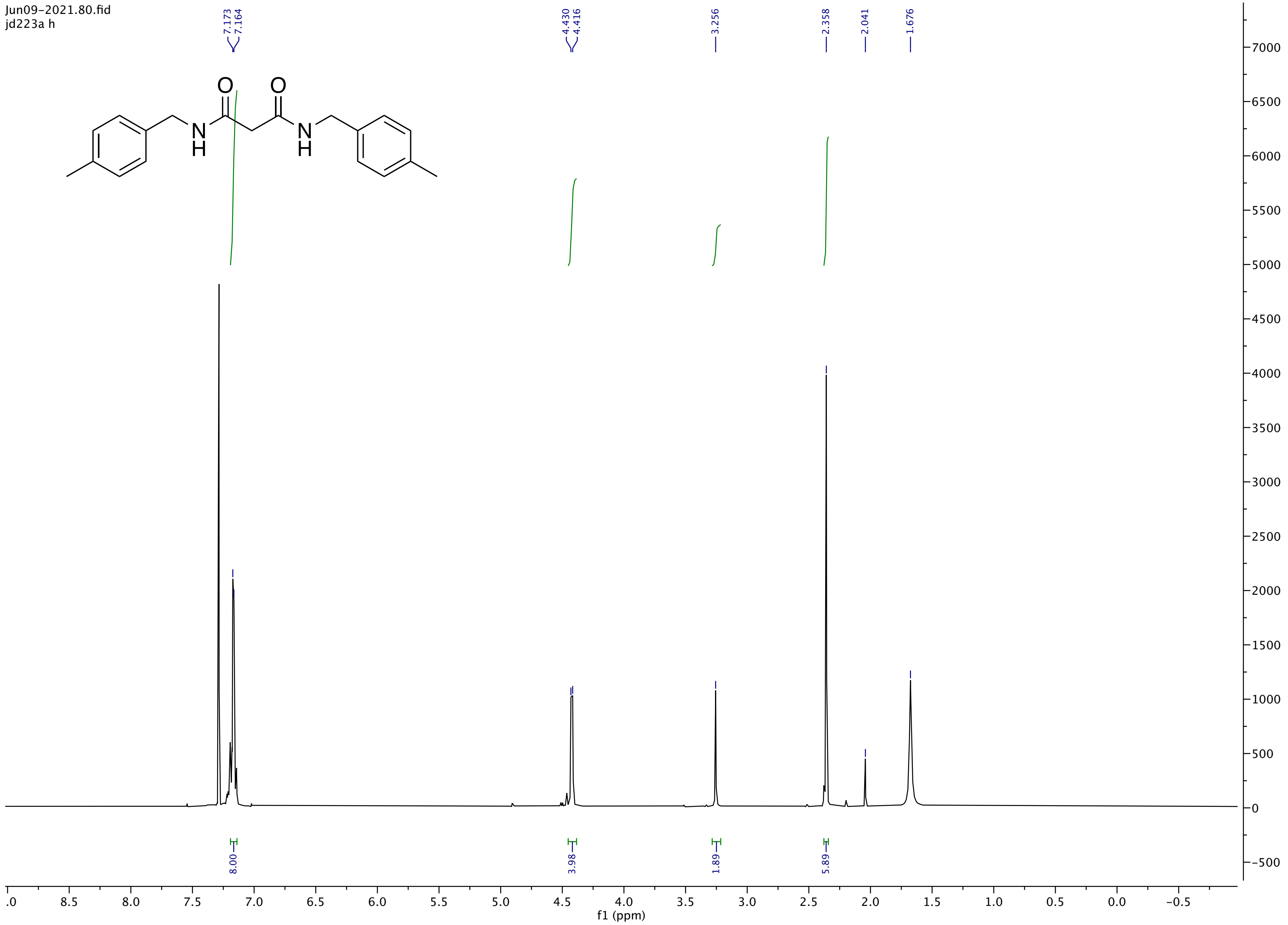

^{13}C NMR spectrum of N^1,N^3-bis(4-methylbenzyl)malonamide (**27**) (101 MHz, $CDCl_3$)

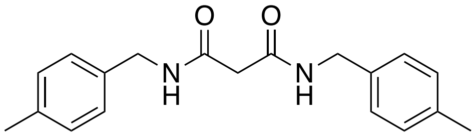

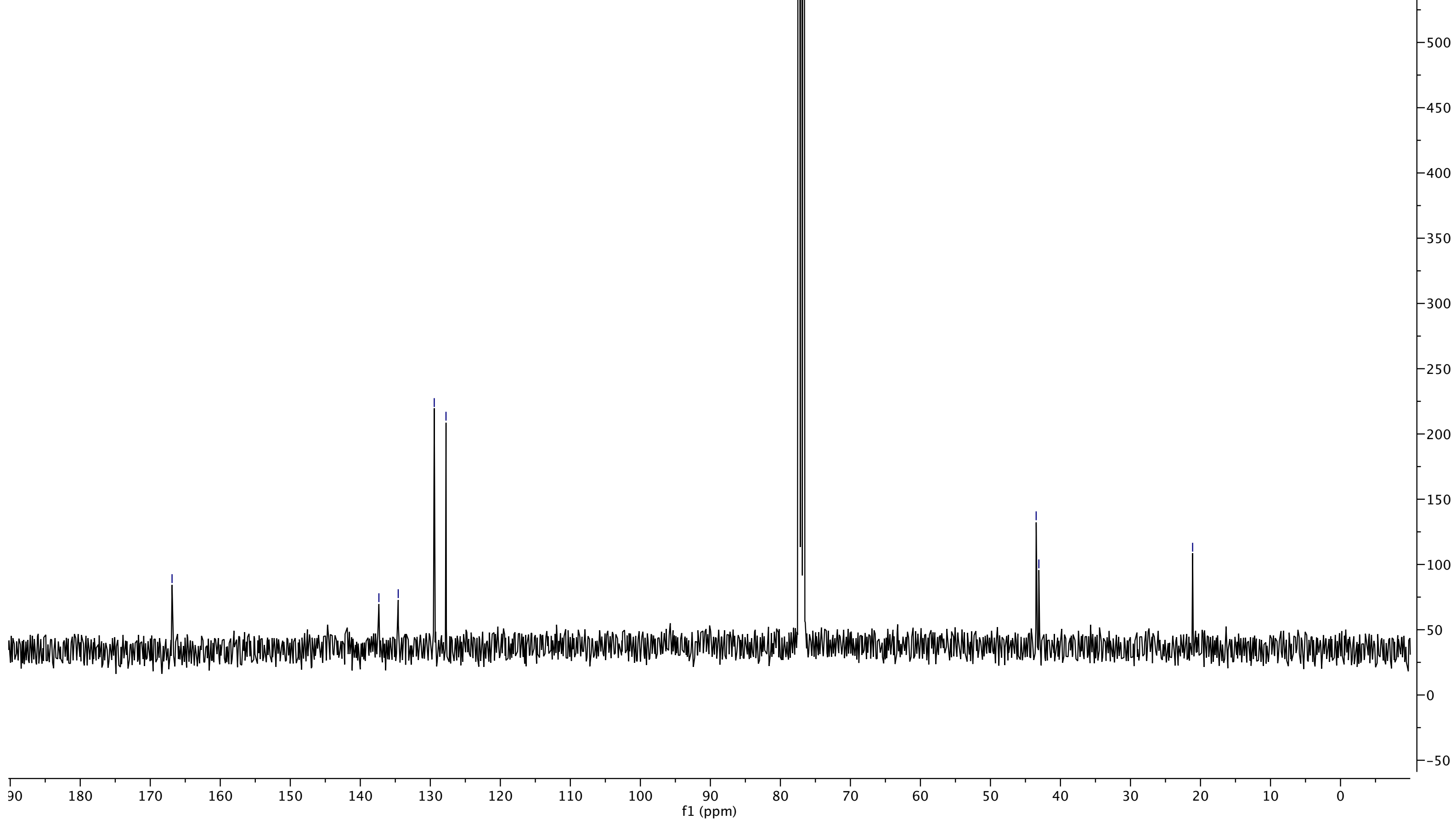

^{1}H NMR spectrum of *N*-methoxy-*N*-methyl-2-phenylacetamide (**28**) (400 MHz, $CDCl_3$)

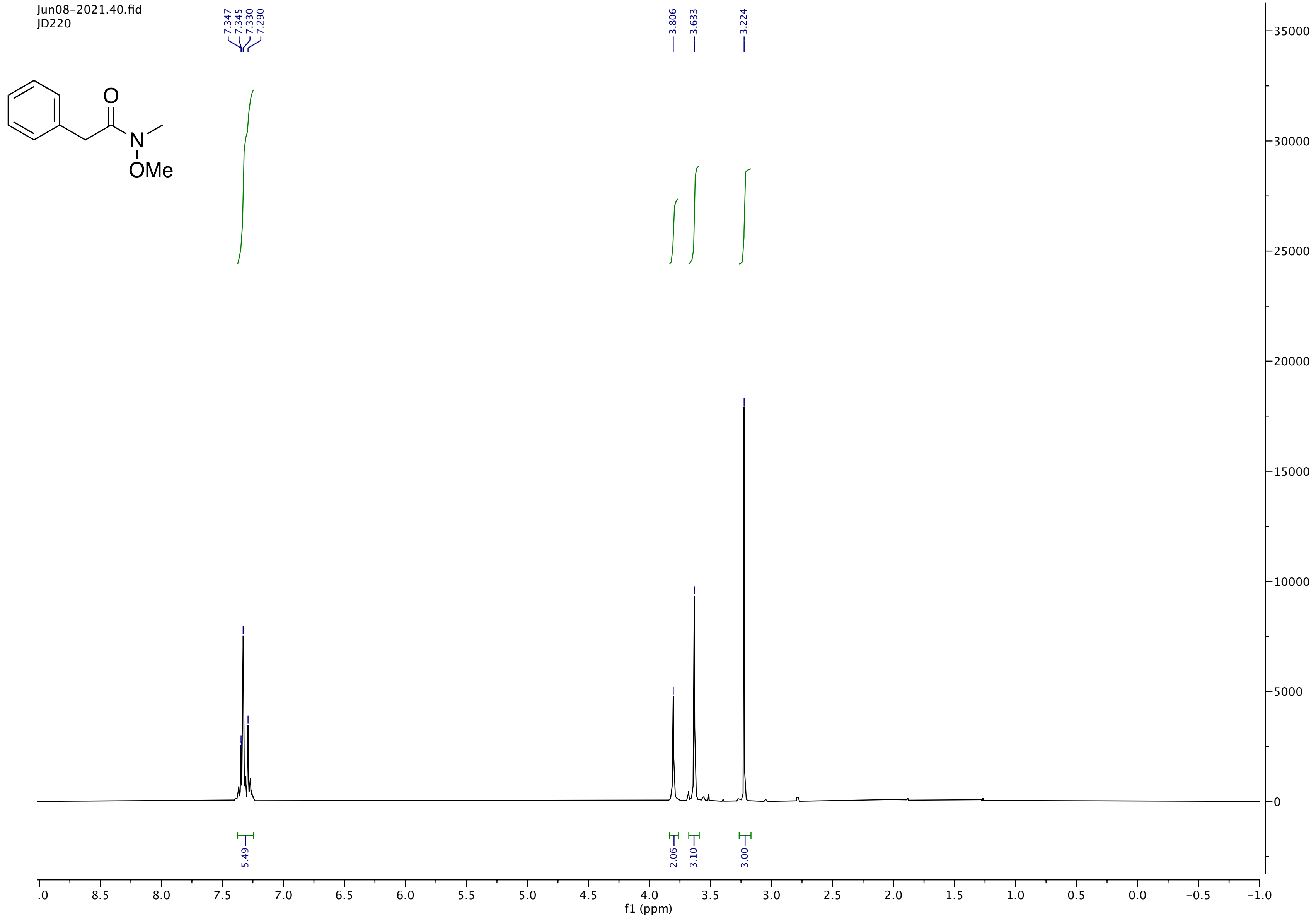

^{13}C NMR spectrum of *N*-methoxy-*N*-methyl-2-phenylacetamide (**28**) (101 MHz, $CDCl_3$)

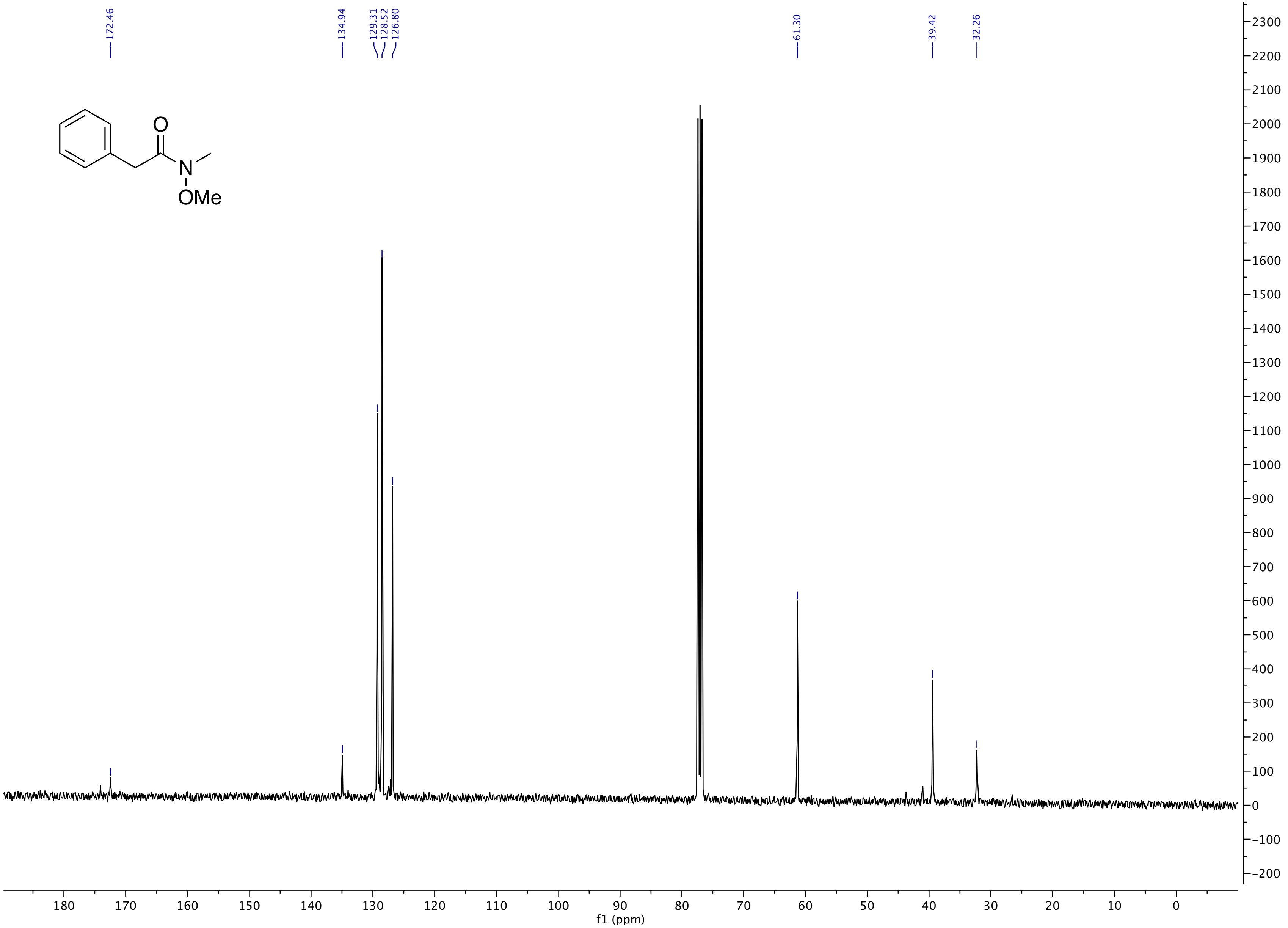

^{1}H NMR spectrum of *N*-hexyl-2-hydroxy-2-phenylacetamide (**29**) (400 MHz, $CDCl_3$)

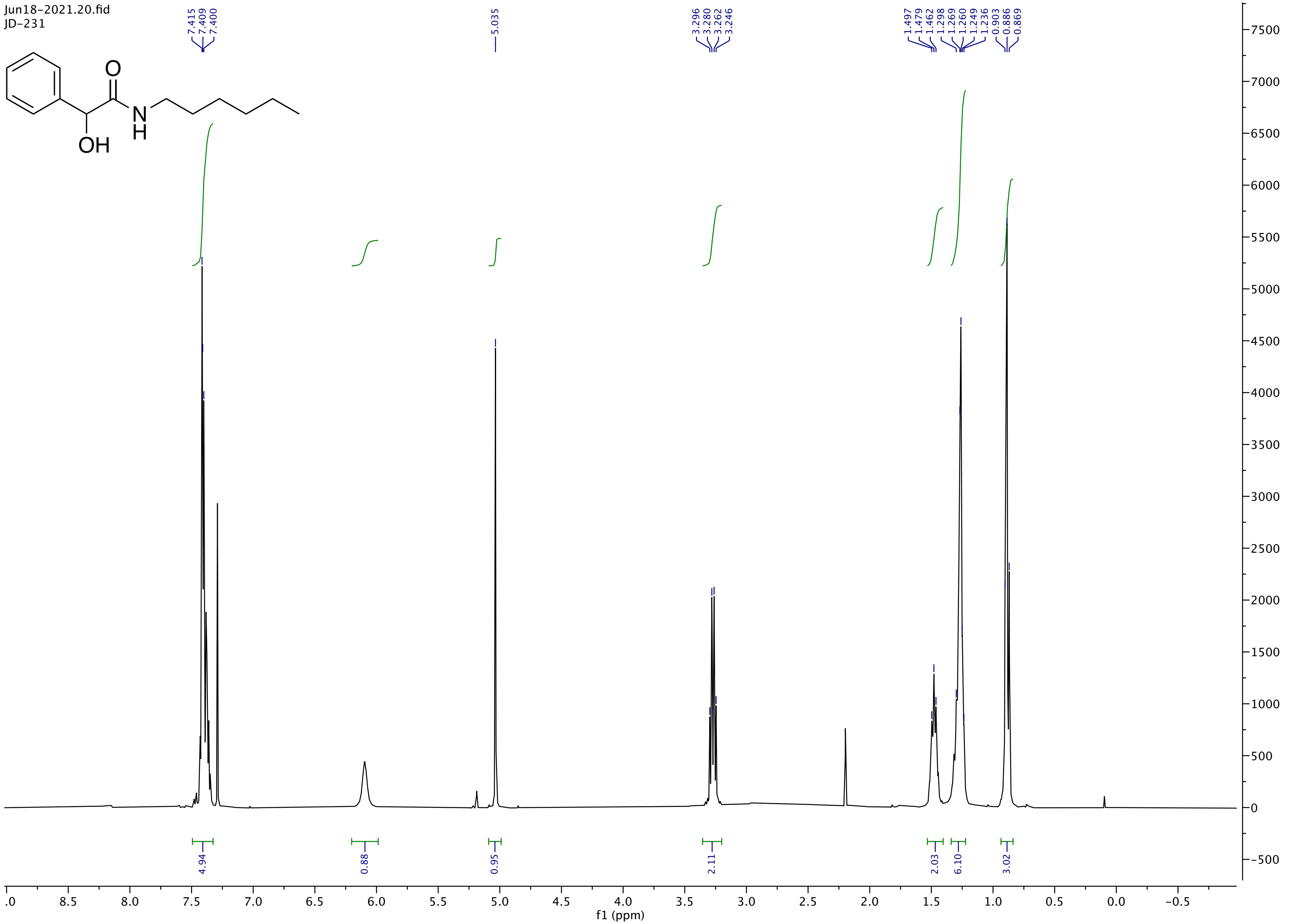

^{13}C NMR spectrum of *N*-hexyl-2-hydroxy-2-phenylacetamide (**29**) (101 MHz, $CDCl_3$)

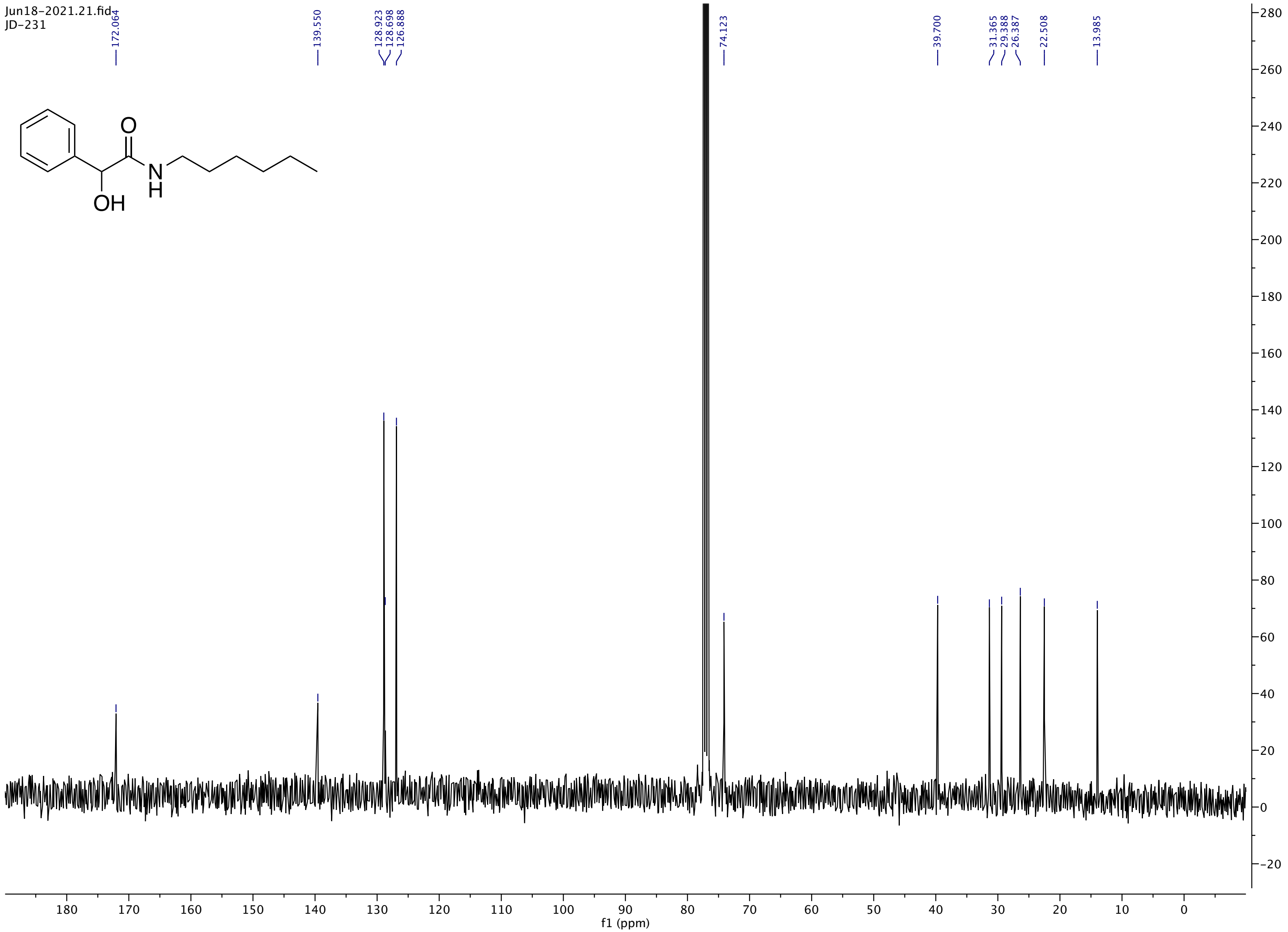

^{1}H NMR spectrum of *N*,*N*-diethyl-2-phenylacetamide (**30**) (400 MHz, $CDCl_3$)

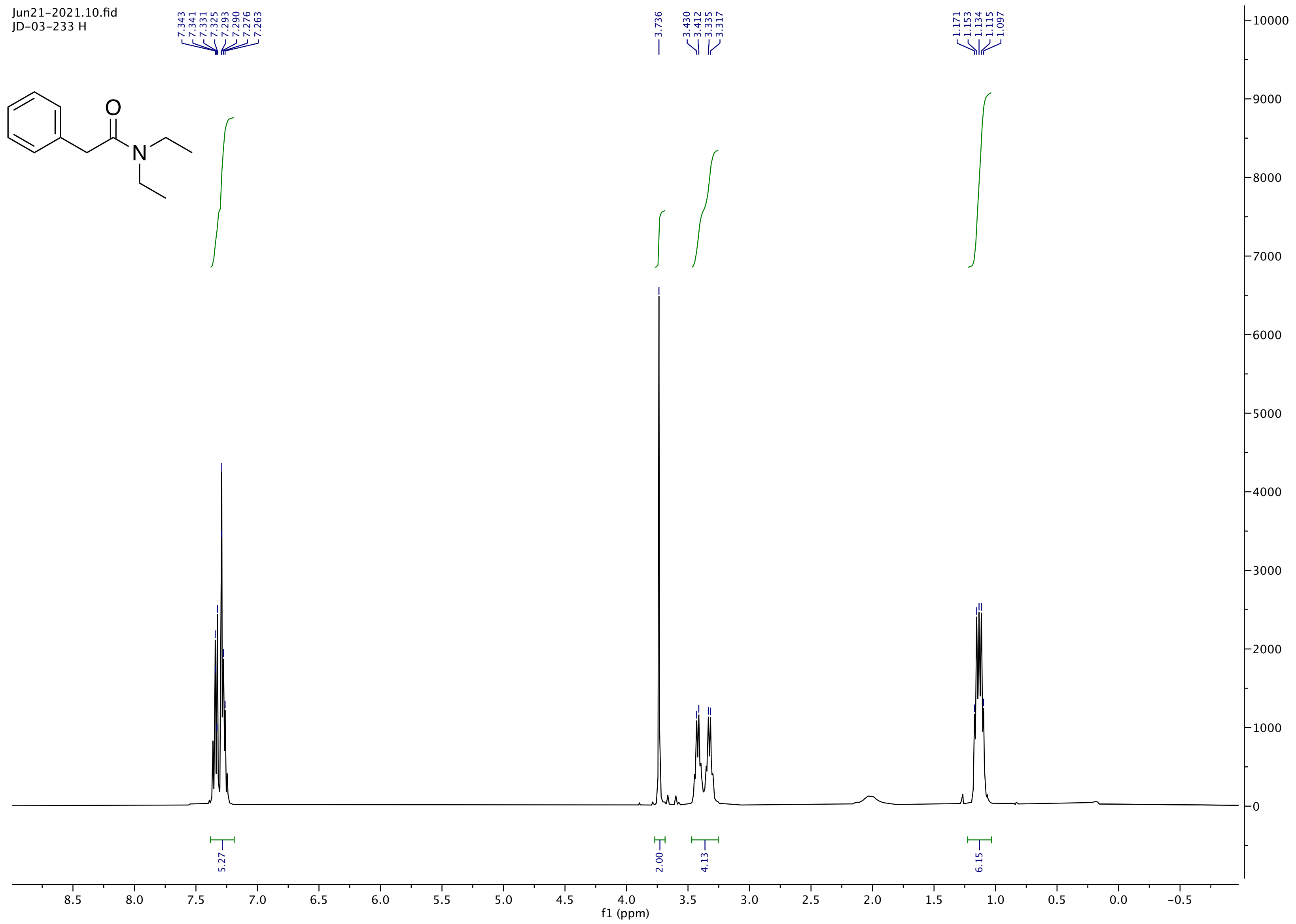

^{13}C NMR spectrum of *N*,*N*-diethyl-2-phenylacetamide (**30**) (101 MHz, $CDCl_3$)

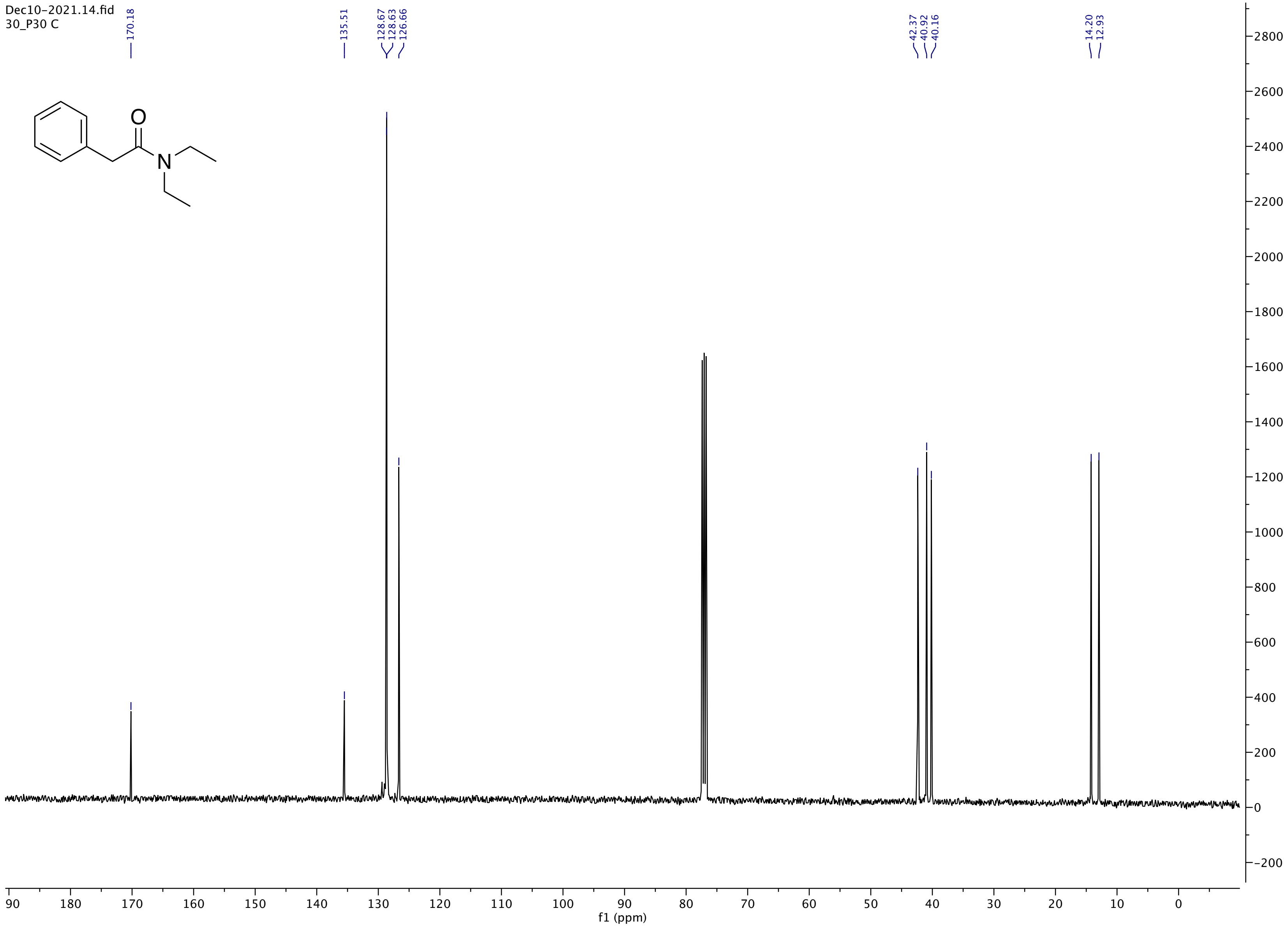

^{1}H NMR spectrum of *N*-phenyl-2-(thiophen-2-yl)acetamide (**31**) (400 MHz, $CDCl_3$)

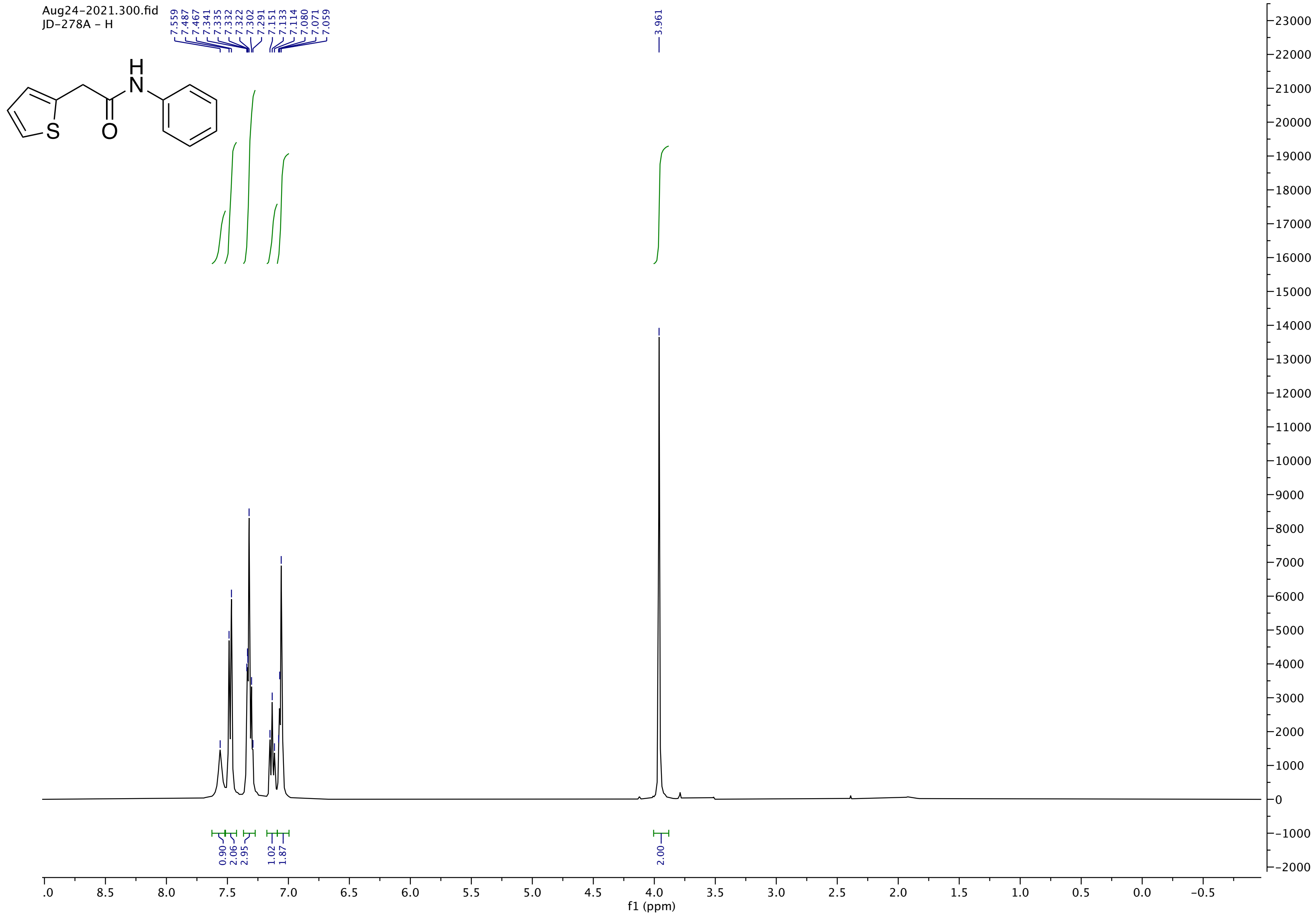

^{13}C NMR spectrum of *N*-phenyl-2-(thiophen-2-yl)acetamide (**31**) (101 MHz, $CDCl_3$)

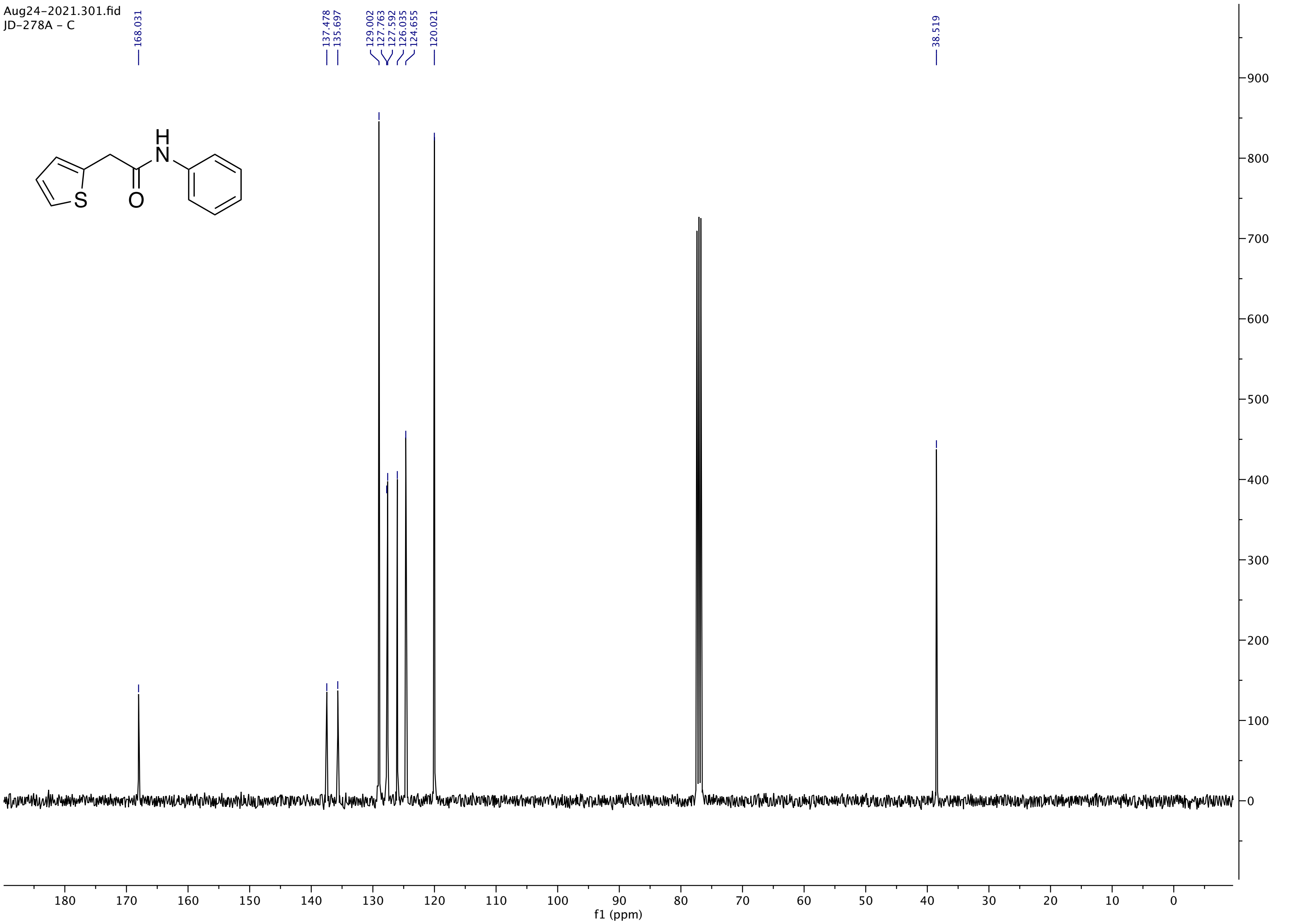

^{1}H NMR spectrum of Benzyl ((*S*)-1-oxo-1-(((*S*)-1-phenylethyl)amino)propan-2-yl)carbamate (**32**) (400 MHz, $CDCl_3$)

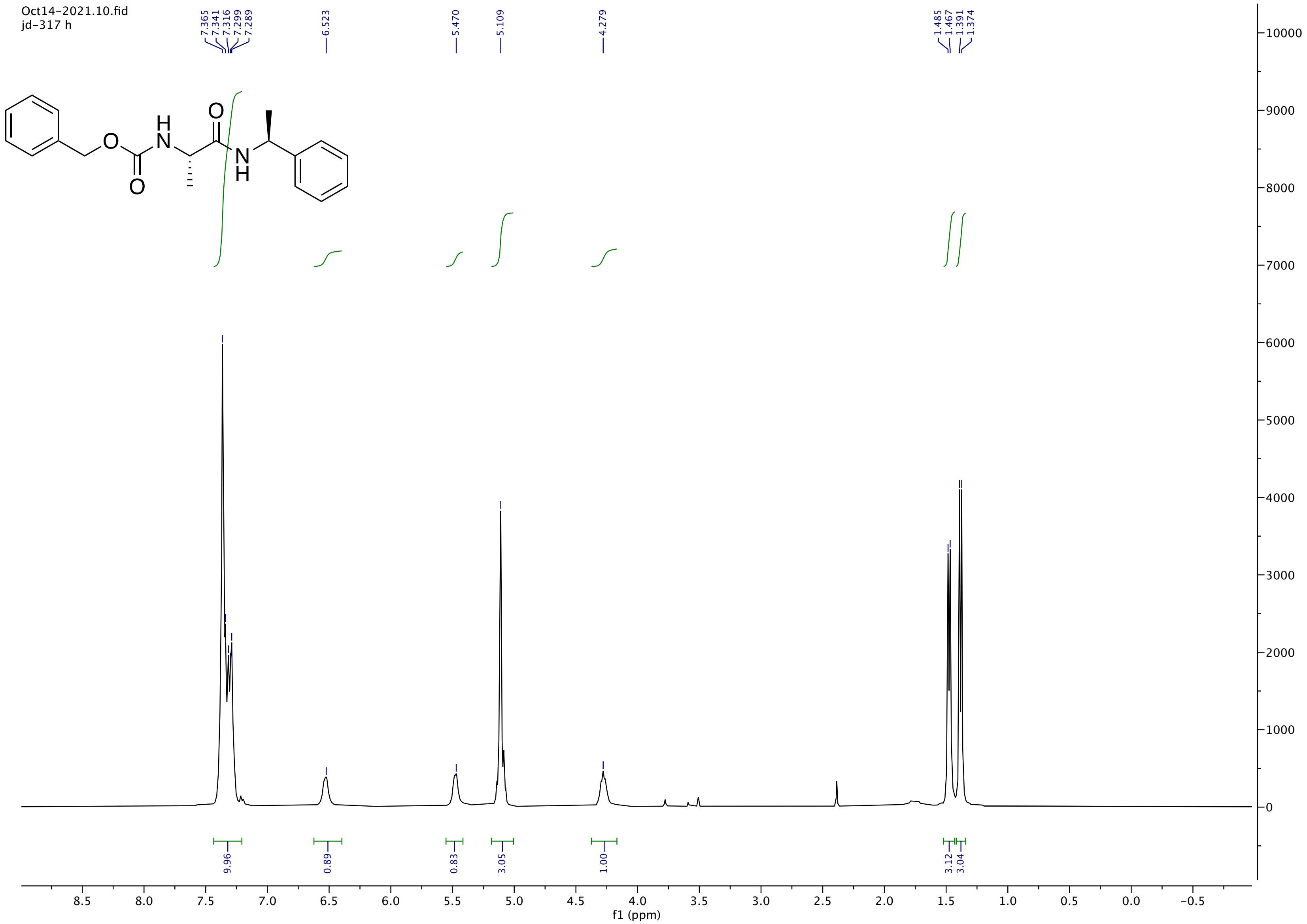

^{13}C NMR spectrum of Benzyl ((*S*)-1-oxo-1-(((*S*)-1-phenylethyl)amino)propan-2-yl)carbamate (**32**) (101 MHz, $CDCl_3$)

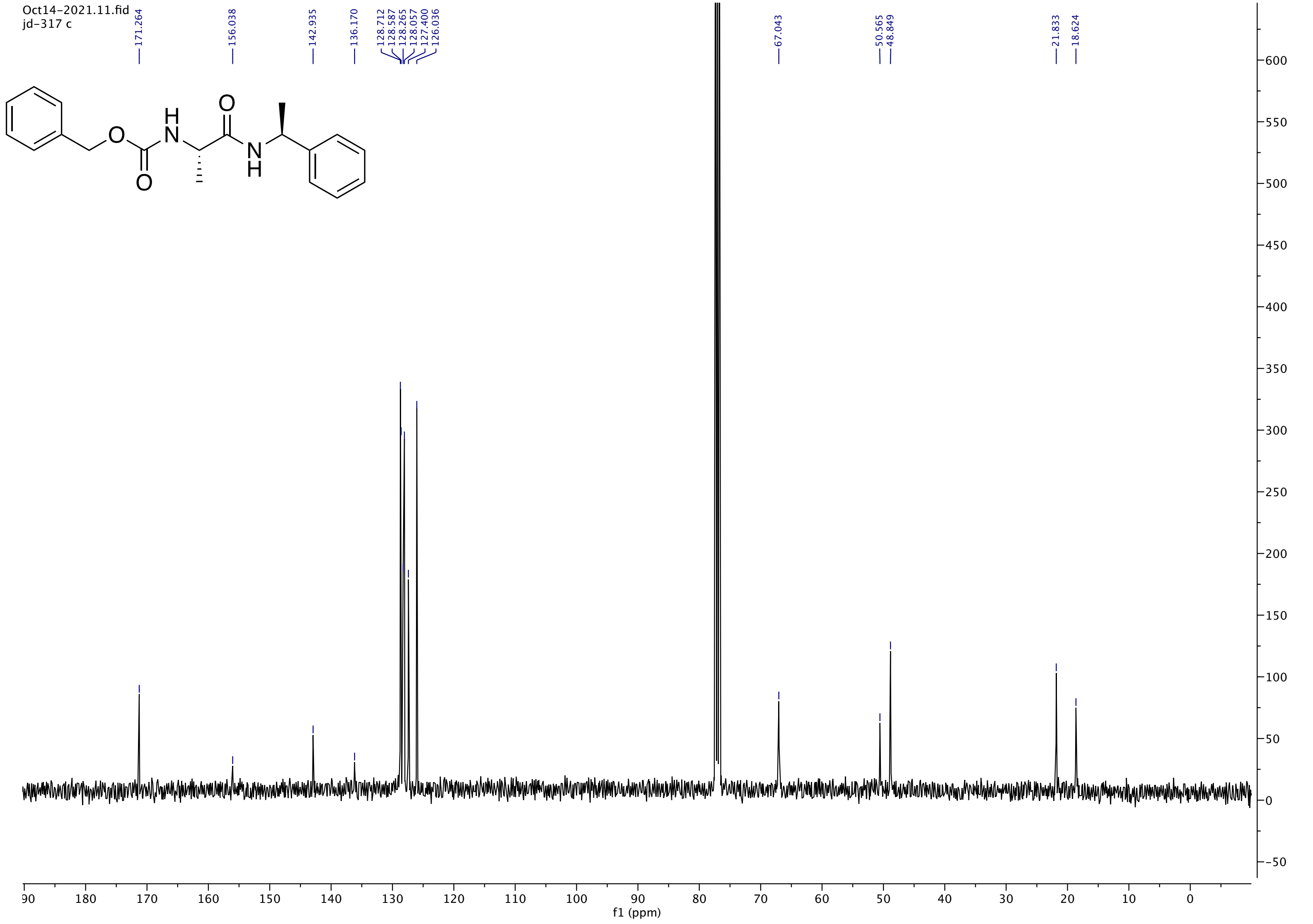

Chiral HPLC determination of enantiomeric purity (top chromatograph) of benzyl (*S*)-(1-(benzylamino)-1-oxopropan-2-yl)carbamate (**22**) by reference to racemic sample (bottom chromatograph).

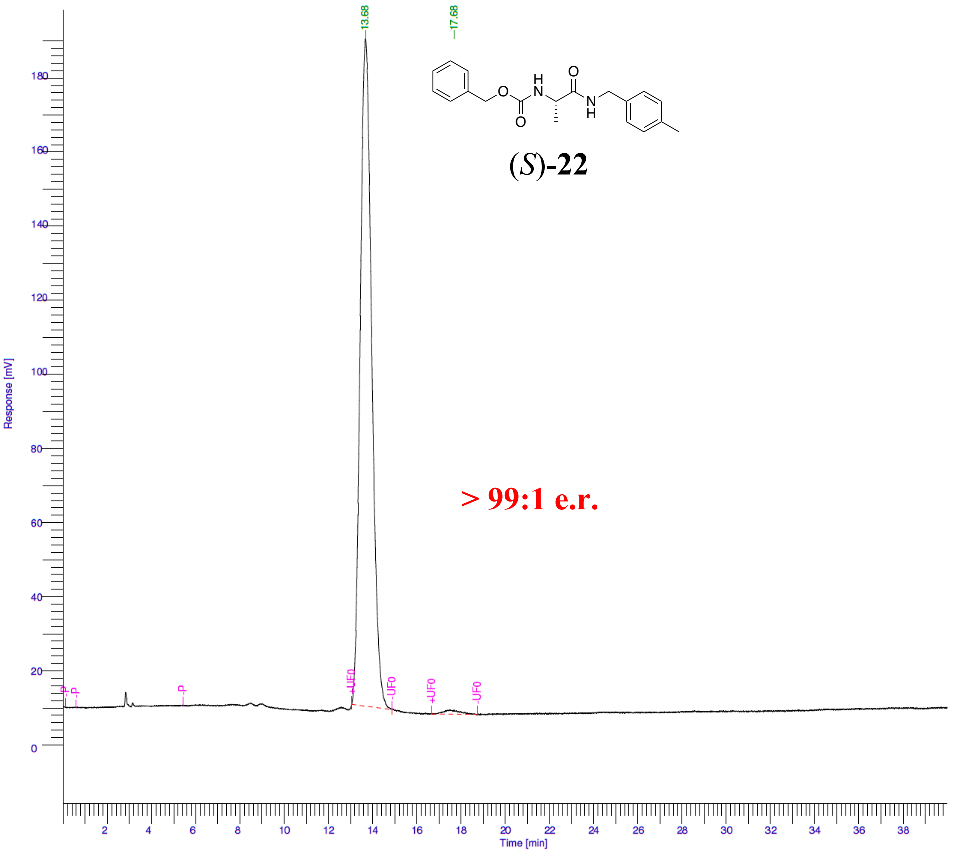

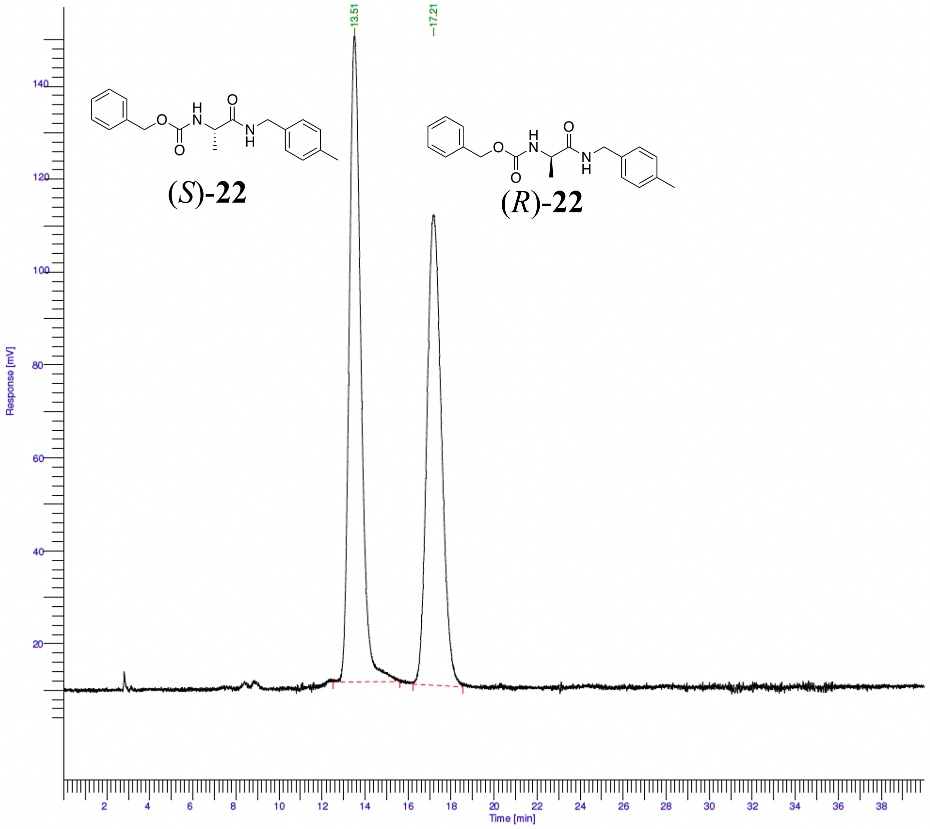

Chiral HPLC determination of enantiomeric purity (top chromatograph) of *tert*-butyl (*S*)-(1-(benzylamino)-1-oxopropan-2-yl)carbamate (**23**) by reference to racemic sample (bottom chromatograph).

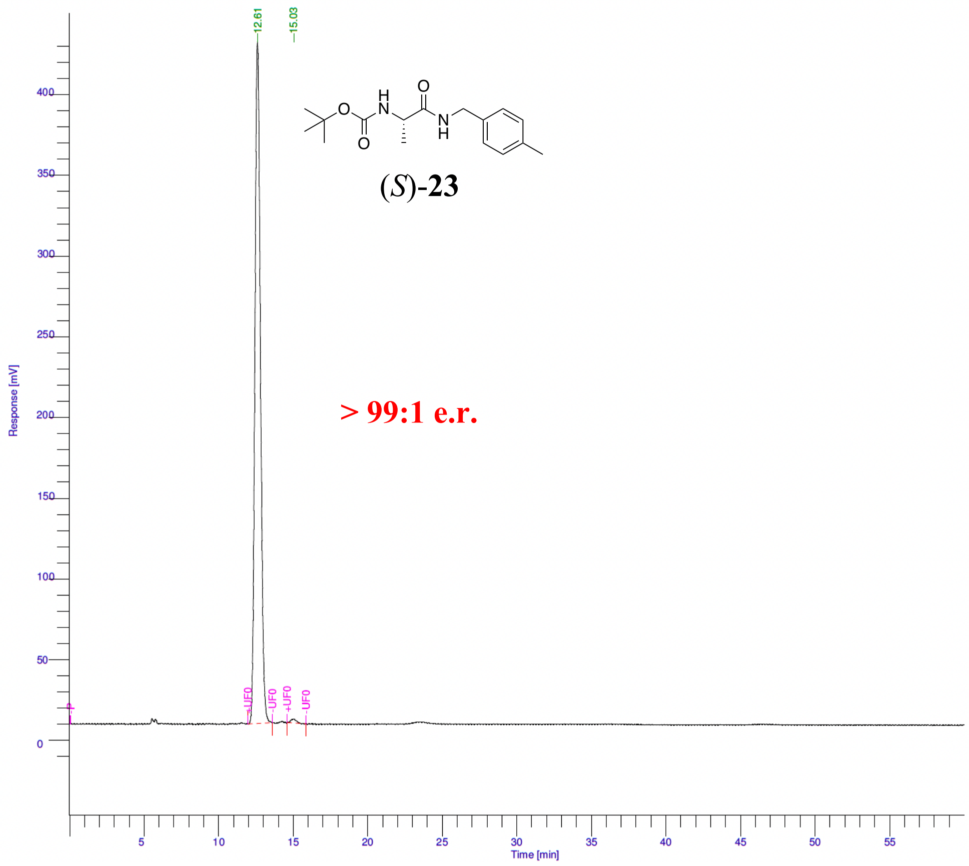

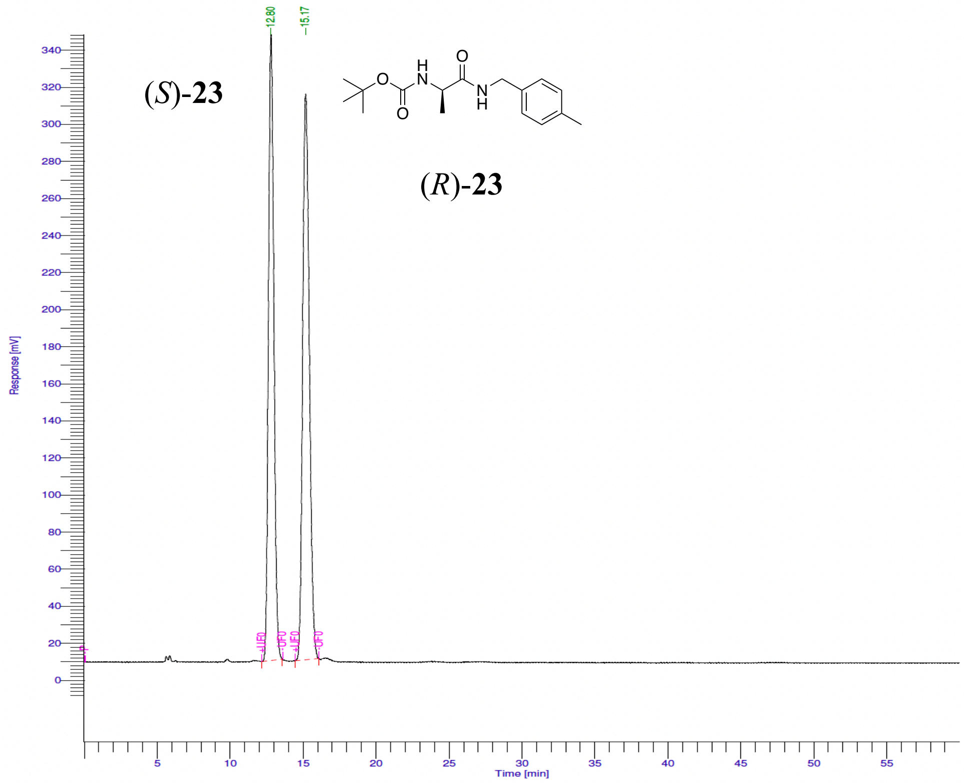

References

(1) Braddock, D. C.; Lickiss, P. D.; Rowley, B. C.; Pugh, D.; Purnomo, T.; Santhakumar, G.; Fussell, S. J. Tetramethyl Orthosilicate (TMOS) as a Reagent for Direct Amidation of Carboxylic Acids. *Org. Lett.* **2018**, *20*, 950–953. https://doi.org/10.1021/acs.orglett.7b03841.

(2) Tang, Y.; Li, M.; Gao, H.; Rao, G.; Mao, Z. Efficient Pd-Catalyzed Hydrodehalogenation of o - Haloanilides in Water. *Synlett* **2020**, *31*, 1121–1125. https://doi.org/10.1055/s-0040-1707116.

(3) Zhu, Y. P.; Sergeyev, S.; Franck, P.; Orru, R. V. A.; Maes, B. U. W. Amine Activation: Synthesis of *N*-(Hetero)Arylamides from Isothioureas and Carboxylic Acids. *Org. Lett.* **2016**, *18*, 4602–4605. https://doi.org/10.1021/acs.orglett.6b02247.

(4) Ramachandran, P. V.; Hamann, H. J. Ammonia-Borane as a Catalyst for the Direct Amidation of Carboxylic Acids. *Org. Lett.* **2021**, *23*, 2938-2942. https://doi.org/10.1021/acs.orglett.1c00591.

(5) Lundberg, H.; Tinnis, F.; Adolfsson, H. Titanium(IV) Isopropoxide as an Efficient Catalyst for Direct Amidation of Nonactivated Carboxylic Acids. *Synlett* **2012**, *23*, 2201–2204. https://doi.org/10.1055/s-0032-1316993.

(6) Forni, J. A.; Micic, N.; Connell, T. U.; Weragoda, G.; Polyzos, A. Tandem Photoredox Catalysis: Enabling Carbonylative Amidation of Aryl and Alkylhalides. *Angew. Chem. Int. Ed.* **2020**, *59*, 18646–18654. https://doi.org/10.1002/anie.202006720.

(7) Iwaniuk, D. P.; Wolf, C. A Versatile and Practical Solvating Agent for Enantioselective Recognition and NMR Analysis of Protected Amines. *J. Org. Chem.* **2010**, *75*, 6724–6727. https://doi.org/10.1021/jo101426a.

(8) Blum, J.; Fisher, A.; Greener, E. The Catalytic Decomposition of Secondary Carboxamides by Transition-Metal Complexes. *Tetrahedron* **1973**, *29*, 1073–1081. https://doi.org/10.1016/0040-4020(73)80064-X.

(9) Barajas, J. G. H.; Méndez, L. Y. V.; Kouznetsov, V. V.; Stashenko, E. E. Efficient Synthesis of New N-Benzyl- or N-(2-Furylmethyl)Cinnamamides Promoted by the “green” Catalyst Boric Acid, and Their Spectral Analysis. *Synthesis* **2008**, 377–382. https://doi.org/10.1055/s-2008-1032039.

(10) Jin, X.; Kataoka, K.; Yatabe, T.; Yamaguchi, K.; Mizuno, N. Supported Gold Nanoparticles for Efficient α-Oxygenation of Secondary and Tertiary Amines into Amides. *Angew. Chem. Int. Ed.* **2016**, *128*, 7328–7333. https://doi.org/10.1002/anie.201602695.

(11) Mirza, B. An Efficient Metal-Free Synthesis of 2-Amino-Substituted-4(3H)-Quinazolinones. *Tetrahedron Lett.* **2016**, *57*, 146–147. https://doi.org/10.1016/j.tetlet.2015.11.085.

(12) Chen, I. L.; Wang, T. C.; Chen, Y. L.; Tzeng, C. C. Aluminium Chloride-Catalyzed Intermolecular vs Intramolecular Friedel-Crafts Reaction of Acrylanilides and 3-Chloropropanamides. *J. Chinese Chem. Soc.* **2000**, *47*, 155–162. https://doi.org/10.1002/jccs.200000018.

(13) Li, J.; Lear, M. J.; Hayashi, Y. Sterically Demanding Oxidative Amidation of α-Substituted Malononitriles with Amines Using O_2. *Angew. Chem. Int. Ed.* **2016**, *128*, 9206–9210. https://doi.org/10.1002/anie.201603399.

(14) Ghosh, S. C.; Li, C. C.; Zeng, H. C.; Ngiam, J. S. Y.; Seayad, A. M.; Chen, A. Mesoporous Niobium Oxide Spheres as an Effective Catalyst for the Transamidation of Primary Amides with Amines. *Adv. Synth. Catal.* **2014**, *356*, 475–484. https://doi.org/10.1002/adsc.201300717.

(15) Yu, W.; Yang, S.; Xiong, F.; Fan, T.; Feng, Y.; Huang, Y.; Fu, J.; Wang, T. Palladium-Catalyzed Carbonylation of Benzylic Ammonium Salts to Amides and Esters: Via C-N Bond Activation. *Org. Biomol. Chem.* **2018**, *16*, 3099–3103. https://doi.org/10.1039/c8ob00488a.

(16) Shao, J.; Huang, X.; Wang, S.; Liu, B.; Xu, B. A Straightforward Synthesis of *N*-Monosubstituted α-Keto Amides via Aerobic Benzylic Oxidation of Amides. *Tetrahedron* **2012**, *68*, 573–579. https://doi.org/10.1016/j.tet.2011.11.005.
